# Research and Clinical Progress of Therapeutic Tumor Vaccines

**DOI:** 10.3390/vaccines13070672

**Published:** 2025-06-23

**Authors:** Chunyan Dong, Zhuang Li, Dejiang Tan, Huimin Sun, Jinghui Liang, Dexian Wei, Yiyang Zheng, Linyu Zhang, Sihan Liu, Yu Zhang, Junzhi Wang, Qing He

**Affiliations:** 1 State Key Laboratory of Drug Regulatory Sciences, National Institutes for Food and Drug Control, Beijing 102629, China; dongchunyan@nifdc.org.cn (C.D.); lizhuang0422@163.com (Z.L.); tandj@nifdc.org.cn (D.T.); sunhm@nifdc.org.cn (H.S.); liangjinghui100@163.com (J.L.); 15195222136@163.com (Y.Z.); zly6802024@163.com (L.Z.); zhangyu2024@nifdc.org.cn (Y.Z.); wangjz@nifdc.org.cn (J.W.); 2 Department of Experimental Pharmacology and Toxicology, School of Pharmaceutical Sciences, Jilin University, Changchun 130021, China; weidx24@mails.jlu.edu.cn; 3 School of Pharmacy, Shenyang Pharmaceutical University, Shenyang 110016, China; 13052799837@163.com

**Keywords:** therapeutic tumor vaccines, clinical progress

## Abstract

Therapeutic cancer vaccines are a new growth point of biomedicine with broad industrial prospects in the post-COVID-19 era. Many large international pharmaceutical companies and emerging biotechnology companies are deploying different tumor therapeutic cancer vaccine projects, focusing on promoting their clinical transformation, and the vaccine industry has strong momentum for development. Such vaccines are also the core engine and pilot site for the development of new vaccine targets, new vectors, new adjuvants, and new technologies, which play a key role in promoting the innovation and development of vaccines. Various therapeutic cancer vaccines, such as viral vector vaccines, bacterial vector vaccines, cell vector vaccines, peptide vaccines, and nucleic acid vaccines, have all been applied in clinical research. With the continuous development of technology, therapeutic cancer vaccines are evolving towards the trends of precise antigens, efficient carriers, diversified adjuvants, and combined applications. For instance, the rapidly advancing mRNA-4157 vaccine is a typical representative that combines personalized antigens with efficient delivery vectors (lipid nanoparticles, LNPs), and it also shows synergistic advantages in melanoma patients treated in combination with immune checkpoint inhibitors. In this article, we will systematically discuss the current research and development status and clinical research progress of various therapeutic cancer vaccines.

## 1. Introduction

Cancer has become a major disease that seriously threatens human health and life worldwide and has always been the core focus of the field of medical research. According to the latest statistics from the International Agency for Research on Cancer (IARC), there were approximately 20 million new cancer cases worldwide in 2022, and nearly 9.7 million people died from cancer [1]. For cancer, traditional treatment methods mainly include surgery, chemotherapy, and radiotherapy. Although they have improved the survival status of patients to a certain extent, their therapeutic effects still have significant limitations. Surgical methods often have difficulty curing metastatic cancers completely. While chemotherapy and radiotherapy kill tumor cells, they also severely damage normal tissues, thereby triggering many adverse reactions and having a significant impact on patients’ lives. In recent years, with the continuous in-depth research on tumor immunology, immunotherapy has gradually emerged and brought new hope for tumor treatment. Such as immune checkpoint inhibitors, cell therapy, tumor vaccines, etc., have become important means of tumor immunotherapy [2,3,4]. Among them, tumor vaccines, as an emerging immunotherapy strategy, have been the focus of much attention in recent years. They specifically recognize and kill tumor cells by activating or enhancing the immune system of cancer patients, thereby achieving the purpose of treating tumors.

**Figure 1:**
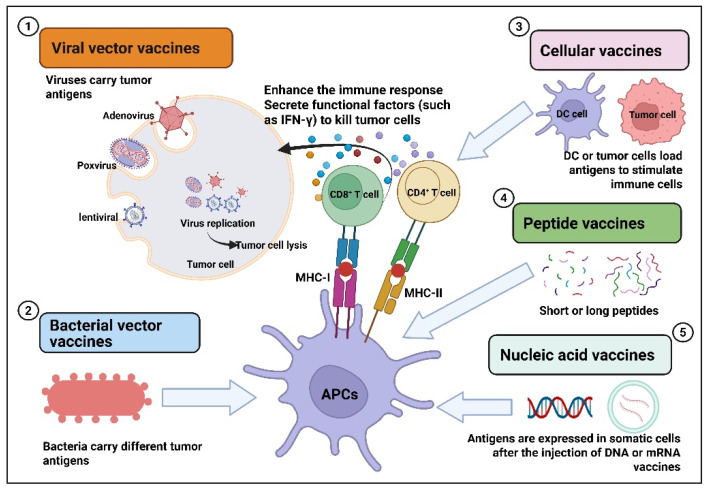
Mechanism of action of therapeutic cancer vaccines.

Tumor vaccines are those that deliver tumor antigens, such as lysed tumor cells, tumor-associated proteins or peptides, RNA or DNA expressing tumor antigens, into the patient’s body to activate the immune response and exert anti-tumor effects [5,6,7,8]. Tumor vaccines are divided into preventive tumor vaccines and therapeutic tumor vaccines. Preventive tumor vaccines are mainly designed against the pathogens that cause tumors, such as the HPV vaccine for preventing cervical cancer [9,10]. Multiple HPV vaccines have been clinically approved globally. For example, the bivalent vaccine (2vHPV): Cervarix^®^, the quadrivalent vaccine (4vHPV): Gardasil^®^, and the nonavalent vaccine (9vHPV): Gardasil9^®^. Therapeutic cancer vaccines are mainly designed for tumor antigens (tumor-associated antigens, specific antigens), stimulating the body to produce specific immune responses to kill tumors. Such vaccines have the characteristics of broad-spectrum and personalization and have important advantages and significance. For therapeutic cancer vaccines, several marketed products have been launched, such as the BCG vaccine (TheraCys^®^), the dendritic cell vaccine Provenge^®^, the oncolytic herpesvirus vaccine (Imlygic^®^), and the peptide vaccine (Cimavax^®^), which are used to treat prostate cancer, melanoma, and renal cell carcinoma. So far, there are still many therapeutic cancer vaccines under research and development worldwide, mainly including viral vector type, bacterial vector type, cell vector type, peptide type, and nucleic acid type. Various therapeutic cancer vaccines combat tumors by directly killing or enhancing the immune response (Figure 1). For viral vector tumor vaccines, they exert anti-tumor effects through multiple pathways. For example, the direct oncolytic effect is that after the virus invades tumor cells, it replicates in large quantities and eventually lyses the tumor cells [11,12,13]. In addition, viral vector vaccines also inhibit tumors by activating immune responses, altering the tumor microenvironment, and disrupting the blood supply to tumors [14,15,16,17,18,19]. Bacteria also have their unique advantages as delivery carriers. For instance, bacterial vaccines loaded with drugs can spontaneously target and colonize tumor tissues after being absorbed [20]. Vaccines based on bacterial vectors present antigens carried by APCs and are recognized by T cells, which activate CD4^+^ and CD8^+^ T cells, thereby enhancing the immune response and inducing apoptosis of tumor cells [20,21,22,23,24]. The mechanism of action of cell vaccines is to introduce tumor antigens or immune-stimulating molecules into the patient’s body through cell vectors, activate the patient’s own immune system, and induce an immune response to achieve the purpose of controlling or eliminating tumors [25,26,27,28,29]. With the development of molecular biology techniques, the tumor antigens recognized by the immune system have been identified. Designing and developing synthetic peptides corresponding to the antigenic epitopes of tumor-reactive lymphocytes has become an important means of treating tumors. Designing peptide-based vaccines to stimulate anti-tumor T-cell responses has many advantages, such as ease of manufacturing and quality control, as well as showing good safety in existing clinical studies [30]. For effective peptide tumor vaccines, their anti-tumor mechanism of action depends on activated CD8^+^ and CD4^+^ T cells [31,32,33]. Nucleic acid vaccines include two types: DNA vaccines and mRNA vaccines. For DNA vaccines, DNA needs to be transported to the cell nucleus for transcription and then translated into the cytoplasm [34]. Vaccines based on mRNA can be directly expressed in the cytoplasm of transfected cells [35]. Nucleic acid vaccine antigens rely on somatic expression and release and then transfer to local APCs for presentation, activating CD8^+^ and/or CD4^+^ T cells [36]. The main indications of various therapeutic cancer vaccines include prostate cancer, lung cancer, glioblastoma, melanoma, breast cancer, liver cancer, etc. [7,37,38,39,40,41,42]. With the continuous advancement of technology, tumor immunotherapy has developed rapidly and has become a research and development hotspot in the field of tumor treatment. This article will focus on systematically discussing the current research and development status of various therapeutic cancer vaccines and the updated related clinical research progress in the past five years, aiming to provide strategies and new ideas for the research and transformation of tumor therapeutic vaccines.

## 2. The Progress of Therapeutic Cancer Vaccines

Therapeutic cancer vaccines mainly use tumor antigens and immune adjuvants to induce specific immune responses to kill tumor cells, and anti-tumor T cells are the effector cells expected to be induced by such vaccines. Hundreds of therapeutic cancer vaccines are currently under clinical evaluation, including viral or bacterial vector vaccines, cellular vaccines, peptide vaccines, and nucleic acid vaccines. Next, we will systematically discuss the research progress of different types of therapeutic tumor vaccines.

### 2.1. Viral and Bacterial Vectors for Therapeutic Cancer Vaccines

#### 2.1.1. Viral Vector Tumor Vaccines

A viral vector is a tool that uses genetic engineering technology to transform viruses and then infects cells to introduce foreign genes into cells and express genes for a long time. Instrumented viral vectors have been widely used in the field of immunotherapy due to their advantages, such as high transfection efficiency, high expression level of exogenous genes, strong targeting, strong killing effect, and strong immune activation ability [43]. Virus vectors mainly include lentivirus, adenoviruses and adeno-associated viruses, poxvirus, herpesvirus, and oncolytic virus. Viral vector-based therapeutic vaccines for tumors have the following advantages: Due to the natural infection ability of viruses, their antigen delivery capacity is significantly superior to that of non-viral vectors (such as naked DNA, RNA, liposomes, etc.) [18,19]. In addition, their strong inherent adjuvant effect enables the vaccine to strongly activate the body’s innate immune response after immunization, thereby effectively initiating subsequent T-cell-mediated anti-tumor immune responses. The immunogenicity of the vector itself is crucial for breaking through the anti-tumor immune-suppressive microenvironment [20]. However, traditional viral vectors also have certain limitations. Firstly, there is a limit to the antigen capacity of the vector [21]. For very complex antigen combinations (such as dozens or hundreds of personalized neoantigens) or large gene fragments, they may not be fully accommodated. This limits the breadth and complexity of the expressed antigens, especially in the application of personalized neoantigen vaccines [22]. Moreover, humans have been exposed to various common viruses (such as adenovirus and varicella-zoster virus) in daily life, and neutralizing antibodies and/or T-cell immune memory against the components of the viral vector already exist in the body [23]. Pre-existing antibodies will rapidly neutralize the injected vector particles, preventing them from infecting target cells and delivering antigens, resulting in a sharp decline or even ineffectiveness of the vaccine’s immunogenicity. Due to the immune system’s memory of the vector, high-titer neutralizing antibodies will be rapidly produced after the first vaccination, making subsequent booster immunizations with the same vector very difficult or ineffective, which is a core challenge. Currently, there are five marketed oncolytic virus products in the world: Rigvir (Latvia), Oncorine (China), IMLYGIC (USA), Adstiladrin (USA), and DELYTACT (Japan), with indications including melanoma, head and neck squamous cell carcinoma, bladder cancer and glioma [44,45,46,47,48]. Next, we will systematically describe the research progress of therapeutic cancer vaccines based on viral vectors (Table 1).

##### Adenoviruses and Adeno-Associated Viruses

Adenovirus vectors can efficiently deliver tumor-associated antigens (TAAs) or tumor-specific neoantigens, inducing a strong T-cell immune response. For example, Ad5-E1A-based adenovirus vector vaccines have shown good antigen delivery in breast and ovarian cancer. Recurrent respiratory papilloma (RRP) is a stubborn neoplastic disease associated with chronic HPV6 or 11 infection, causing severe hoarseness and airway obstruction, and there is no approved therapy [49]. PRGN-2012 is a new type of gorilla adenovirus immunotherapy drug that can enhance specific T-cell immunity against HPV 6/11 [50]. In the Phase 1 clinical trial (NCT04724980), PRGN-2012 was first used to treat severe and invasive RRP in adults and showed good clinical benefits. It was generally safe, and the complete response rate in the highest-dose group reached 50% [50].

Adenovirus vector vaccines against cancer are a strong area of preclinical and clinical research. There are many studies on therapeutic cancer vaccines based on adenovirus vectors that have entered the clinical stage, but most of them are in the clinical Phase 1–2. Adenovirus vector vaccines are mainly used for the treatment of glioblastoma (NCT05686798, NCT05914935, NCT03896568, NCT02026271, NCT02798406), Prostate Cancer (NCT02555397, NCT01931046, NCT00583024, NCT04097002, NCT00583752, NCT04374240), lung cancer (NCT06618391, NCT02879760), melanoma (NCT04217473, NCT03003676, NCT05664139, NCT05222932) and other cancers (Table 1). The adenovirus vector vaccines that have made relatively rapid progress are A and Recombinant Human Adenovirus (H101). H101 is the world’s first approved virus drug and has an anti-tumor effect on liver cancer. In a Phase 4 clinical trial (NCT05124002) [51], the study aimed to further verify the efficacy and safety of H101 combined with the chemotherapy drug HAIC in the treatment of intrahepatic massive cholangiocarcinoma. Previous studies have demonstrated that the progression-free survival (PFS) of HAIC in the treatment of unresectable intrahepatic cholangiocarcinoma is approximately 8 to 10 months, and the one-year progression-free rate is about 40%. The combined treatment of H101 and HAIC is expected to further enhance the therapeutic effect and increase the PFS.

##### Poxvirus

Poxvirus is a double-stranded DNA virus, which can replicate in cells without entering the nucleus and without the risk of gene integration, greatly improving safety [52]. In addition, poxviruses can also insert large foreign genes (25 KB), thus achieving the expression of complex eukaryotic sequences and multiple genes in mammalian cells, ensuring correct post-translational modifications [52]. Because poxviruses have strong immunogenicity and can mask the immune response to the antigens they carry when used as vaccine vectors, subsequently attenuated poxviruses with modified and deleted virulent genes, have been used as vaccine vectors, such as modified vaccinia virus Ankara (MVA) [53]. In a preclinical study, the two prostate cancer-related antigens mPSCA and mSTEAP1 vaccines carried by MVA demonstrated excellent anti-tumor activity in tumor-bearing mouse models [54]. Moreover, carrying both antigens simultaneously had a stronger inhibitory effect on tumors than carrying either mPSCA or mSTEAP1, which demonstrated the advantage of poxviruses carrying multiple antigens simultaneously [54]. JX-594 (Pexa-Vec) is a vaccine based on the varicella virus. In a Phase 1 clinical trial (NCT00629759), JX-594 demonstrated significantly superior complete remission and systemic efficacy for large-volume tumors compared to other similar drugs [55,56]. Reactions at the injection site of JX-594 were observed in the tumor at all doses. However, systemic tumor responses and delivery to distant tumors through the blood require high doses [56]. In a Phase 2 clinical trial (NCT00554372), researchers explored the efficacy of intratumoral injection of high-dose (10^9^ PFU) and low-dose (10^8^ PFU) JX-594 in patients with liver cancer [57]. The results showed that the median overall survival (OS) in the high-dose group reached 14.1 months compared with 6.7 months in the low-dose group [57]. In terms of safety, JX-594 was generally well tolerated at two doses, and no treatment-related deaths were reported [57]. There is still one study of JX-594 entering Phase 3 clinical trials. However, since the clinical benefit of JX-594 plus sorafenib in the treatment of advanced hepatocellular carcinoma (HCC) did not increase and the effect was worse compared with sorafenib alone, the interim analysis failed to reach the primary endpoint and was terminated early. The combined therapy strategy for oncolytic viruses still needs further exploration [58].

##### Other Virus

In addition to adenovirus vectors and poxvirus vectors, vaccines based on other viral vectors have also been applied to tumors (Table 1). In addition to adenovirus vectors and poxvirus vectors, vaccines based on other viral vectors have also been applied to tumors. For example, lentiviral vector vaccines such as Lenti-HPV-07 have been used in clinical studies to treat HPV-associated oropharyngeal squamous cell cancer [59]. In addition, there are also some vaccines based on other viral vectors, such as Vvax001 (Semliki Forest Virus), HSV G207C (herpes simplex virus-1), etc., which are used in clinical studies to treat cervical intraepithelial neoplasia and brain tumors [60,61].

##### Combination Therapy

Therapeutic cancer vaccines delivered by viral vectors are also inhibited by immunosuppressive factors (such as Treg cells and MDSCs) in the tumor microenvironment, which may weaken the therapeutic effect of the vaccine. To improve efficacy, some research has focused on developing strategies that combine viral vaccines with other therapies to address immunosuppression. In a preclinical study, adenovirus vector-delivered tumor neoantigen vaccine combined with anti-PD-1 antibodies significantly enhanced tumor immunogenic, neoantigen-specific CD8^+^ T-cell response and extended overall survival in MC38 tumor-bearing mice [62]. In addition, adenovirus vector-based tumor vaccines in combination with other therapies have been used in clinical trials to treat melanoma (NCT03003676, NCT05664139, NCT05222932) [63,64,65], colon cancer (NCT04166383, NCT06283134) [66,67], glioblastoma (NCT02798406, NCT02026271) [68,69], lung cancer (NCT06125197, NCT06618391, NCT02879760) [70,71,72], pancreatic cancer (NCT03281382, NCT02894944, NCT02705196) [73,74,75], etc. Clinical studies on combined therapy based on other viral vector vaccines are listed in Table 1.

**Table 1:** Clinical study of viral vector vaccines updated in recent 5 years.

Name	Cancer	ROA	Combination Therapy	NCI Number	Phase	Ref
**Adenovirus Vector-Based Therapeutic Cancer Vaccine**						
Ad5-yCD/mutTKSR39rep-ADP	Glioblastoma	i.t.	/	NCT05686798	1	[76]
Recombinant L-IFN adenovirus injection (YSCH-01)	Glioblastoma	Intracapsular	/	NCT05914935	1	[77]
DNX-2401	Glioblastoma	Intra-arterial	/	NCT03896568	1	[78]
Ad-RTS-hIL-12	Glioblastoma	i.t.	Veledimex	NCT02026271	1	[69]
DNX-2401	Glioblastoma	i.t.	Anti-PD-1	NCT02798406	2	[68]
Ad5 peptide transduction domain (PTD)(CgA-E1AmiR122)	Neuroendocrine tumors	Intrahepatic artery	/	NCT02749331	1/2	[79]
NG-641, a tumor-selective transgene-expressing adenoviral vector	Epithelial tumors	i.v.	/	NCT04053283	1	[80]
NG-350A	Epithelial tumor	i.v.	/	NCT03852511	1	[81]
Ad5-yCD/mutTKSR39rep-hIL12	Prostate cancer	i.p.	/	NCT02555397	1	[82]
Ad5-SGE-REIC/Dkk3	Prostate cancer	/	/	NCT01931046	1	[83]
Adenovirus/PSA vaccine	Prostate cancer	s.c.	/	NCT00583024	2	[84]
ORCA-010	Prostate cancer	i.t.	/	NCT04097002	1/2	[85]
Adenovirus/PSA vaccine	Prostate cancer	s.c.	Androgen deprivation therapy	NCT00583752	2	[86]
AdNRGM	Prostate cancer	i.t.	CB1954	NCT04374240	1	[87]
KD01	Cervical cancer	i.t.	/	NCT06552598	1	[88]
Human adenovirus 5 injection (d1-d5)	Cervical cancer	i.t.	Chemotherapy	NCT06455046	2	[89]
Adenoviral-mediated interferon-beta (BG00001)	Pleural malignancies	i.p.	/	NCT00299962	1	[90]
Adenovirus-hIFN-beta	Pleural malignancies	i.p.	/	NCT00066404	1	[91]
Ad5CMV-p53 gene	Lung cancer	/	/	NCT00003649	1	[92]
Ad5 (CEA/MUC1/Brachyury)	Neoplasms Prostate cancer Lung cancer Breast cancer Colon cancer	s.c.	/	NCT03384316	1	[93]
Adenovirus (ColoAd1)	Colon cancer Non-small-cell lung cancer Bladder cancer Resectable renal cell carcinoma	i.t./ i.v.	/	NCT02053220	1	[94]
GVAX	Sarcoma Renal cell carcinoma melanoma	/	/	NCT00258687	1	[95]
Ad/PNP	Head and neck cancer	i.t.	/	NCT03754933	1/2	[96]
Enadenotucirev	Rectal cancer	i.v.	Chemoradiotherapy	NCT03916510	1	[97]
rAd-IFN	Pleural mesothelioma	i.p.	Celecoxib + Gemcitabine	NCT03710876	3	[98]
SCH 721015	Mesothelioma	i.p.	Chemotherapy	NCT01119664	1	[99]
H101	Hepatocellular carcinoma	i.t.	TACE	NCT05872841	2	[100]
H101	Hepatocellular carcinoma	i.t.	Tislelizumab and Lenvatinib	NCT06253598	2	[101]
H101	Hepatocellular carcinoma	Hepatic arterial infusion	/	NCT06685354	2	[102]
H101	Hepatocellular carcinoma	i.t.	Sorafenib	NCT05113290	4	[103]
HAIC of FOLFOX	Hepatocellular carcinoma	Hepatic artery	/	NCT03780049	3	[104]
SynOV1.1	Hepatocellular carcinoma	i.t.	/	NCT04612504	1	[105]
VB-111	Colorectal cancer	i.v.	Anti-PD-1	NCT04166383	2	[66]
BioTTT001	Colorectal cancer	/	Anti-PD-1+ Regorafenib	NCT06283134	1	[67]
BioTTT001	Gastric cancer	i.p.	SOX+ Anti-PD-1	NCT06283121	2	[106]
Recombinant human adenovirus (H101)	Cholangiocarcinoma	i.t.	FOLFOX	NCT05124002	4	[51]
Adenovirus VCN-01	Retinoblastoma	Intravitreal	/	NCT03284268	Not applicable	[107]
Ad5/3-E2F-d24-hTNFa-IRES-hIL2 (TILT-123)	Ovarian cancer	/	Anti-PD-1	NCT05271318	1	[108]
Ad5CMV-p53 gene	Ovarian cancer	i.p.	/	NCT00003450	1	[109]
Ad5/3-E2F-d24-hTNFa-IRES-hIL2	Melanoma	/	/	NCT04217473	1	[110]
ONCOS-102	Melanoma	i.t.	Cyclophosphamide+ Anti-PD-1	NCT03003676	1	[63]
Recombinant human adenovirus type 5	Melanoma	/	Anti-PD-1+ Nab-paclitaxel	NCT05664139	2	[64]
Ad5/3-E2F-d24-hTNFa-IRES-hIL2	Melanoma Head and neck squamous cell carcinoma	/	Anti-PD-L1	NCT05222932	1	[65]
Recombinant human adenovirus type 5	Lung cancer	i.t.	Chemotherapy + Anti-PD-1	NCT06618391	2	[71]
Ad-MAGEA3	Lung cancer	i.m.	Anti-PD-1	NCT02879760	1/2	[72]
Ad5/3-E2F-d24-hTNFa-IRES-hIL2 (TILT-123)	Lung cancer	/	Anti-PD-1	NCT06125197	1	[70]
NG-641	Epithelial tumor	i.v.	Anti-PD-1	NCT05043714	1	[111]
NG-350A	Epithelial tumor	i.v.	Anti-PD-1	NCT05165433	1	[112]
NG-350A	Rectal cancer	i.v.	Chemoradiotherapy	NCT06459869	1	[113]
Ad5-yCD/mutTKSR39rep-hIL12	Pancreatic cancer	i.t.	Chemotherapy	NCT03281382	1	[73]
Ad5-yCD/mutTKSR39rep-ADP	Pancreatic cancer	/	Chemotherapy	NCT02894944	1	[74]
Adenovirus serotype 5/35 encoding TMZ-CD40L and 4-1BBL (LOAd703)	Pancreatic adenocarcinoma Ovarian cancer Biliary carcinoma Colorectal cancer	i.t.	Chemotherapy	NCT03225989	1/2	[114]
LOAd703	Pancreatic adenocarcinoma Ovarian cancer Biliary carcinoma Colorectal cancer	i.t.	Chemotherapy or gemcitabine	NCT03225989	1/2	[114]
LOAd703	Pancreatic cancer	i.t.	Anti-PD-L1	NCT02705196	1	[75]
Theragene^®^, Ad5-yCD/mutTKSR39rep-ADP	Pancreas cancer	/	Radiation	NCT04739046	2	[115]
Adenoviral p53 (Ad-p53)	Solid tumors	i.t.	Anti-PD-1/Anti-PD-L1	NCT03544723	2	[116]
CAdVEC	Solid tumors	i.t.	HER2-specific autologous CAR-T cells	NCT03740256	1	[117]
YSCH-01	Solid tumors	i.t.	/	NCT05180851	1	[118]
Ad5/3-E2F-d24-hTNFa-IRES-hIL2	Solid tumors	/	/	NCT04695327	1	[119]
AdAPT-001	Solid tumors	i.t.	/	NCT04673942	2	[120]
**Poxvirus Vector-Based Therapeutic Cancer Vaccine**						
PROSTVAC-V/F	Prostate cancer	/	GM-CSF	NCT01322490	3	[121,122]
PROSTVAC-V/F	Prostate cancer	s.c.	Anti-PD-1	NCT02933255	1/2	[123]
TG4050	Ovarian carcinoma	s.c.	/	NCT03839524	1	[124]
TG4050	Head and neck cancer	s.c.	/	NCT04183166	1/2	[125]
**Other Vector-Based Therapeutic Cancer Vaccine**						
Lenti-HPV-07	HPV-associated oropharyngeal squamous cell cancer, cervical cancer	i.m.	/	NCT06319963	1/2	[59]
Nous-209 genetic vaccine	Microsatellite unstable solid tumors	/	Anti-PD-1	NCT04041310	1/2	[126]
Vvax001 therapeutic cancer vaccine	Cervical intraepithelial neoplasia	i.m.	/	NCT06015854	2	[127]
HSV G207	Recurrent supratentorial brain tumors	i.t.	/	NCT02457845	1	[127]

**Abbreviation:** Subcutaneous injection (s.c.); intramuscular injection (i.m.); intravenous injection (i.v.); intertumoral injection (i.t.); intraperitoneal injection (i.p.).

#### 2.1.2. Bacterial Vector Tumor Vaccine

Since bacteria can naturally accumulate on tumors and regulate immune responses, it is believed that bacteria have great potential as carriers for tumor vaccines [128,129,130,131]. Redenti et al. developed a vaccine using the probiotic *Escherichia coli* Nissle 1917 as the tumor neoantigen vector, which significantly enhanced safety and immunogenicity, effectively activated the systemic anti-tumor immune response dominated by T cells, and killed the primary tumor and distant metastases [132]. This system utilizes the properties of living drugs to deliver tumor-specific neoantigens in the optimal environment to induce specific, effective, and long-lasting systemic anti-tumor immunity, such as promoting the activation of dendritic cells, neoantigen-specific T cells, and natural killer cells, as well as significantly reducing tumor-infiltrating immunosuppressive bone marrow cells and regulatory T-cell and B-cell populations [132]. Importantly, vaccines based on bacterial vectors have another advantage in that they can be administered orally. For instance, a preclinical study found that oral administration of the modified Salmonella typhimurium VNP20009 induced a significant anti-cancer effect in B16F10 melmelanoma tumor-bearing mice. Moreover, oral administration has less toxicity and is more reversible compared to intraperitoneal administration. This study indicates that oral administration, as a new approach for bacterial application, has a high degree of safety and efficacy [133].

Nowadays, the bacteria mainly used for preparing tumor vaccines include Salmonella, Listeria, Clostridium, Bifidobacterium, etc. However, many studies are still in the preclinical stage, and few have been translated into clinical practice. ADXS11-001 is an inactivated and attenuated Listeria vector vaccine based on the HPV16 E7 antigen developed by Advaxis. In a Phase 2 clinical study, ADXS11-001 demonstrated good safety and tolerability in patients with cervical cancer [134]. The median overall survival was comparable in the ADXS11-001 group (8.28 months) and the ADXS11-001 + cisplatin group (8.78 months), and the progression-free survival (6.10 months vs. 6.08 months) and the overall response rate (17.1% vs. 14.7%) were also similar [134]. ADXS11-001 was generally well tolerated, and the severity of adverse events was mainly mild to moderate [134]. ADXS11-001 is also being used in a Phase 2 clinical study (NCT02399813) for the treatment of anorectal cancer [135]. Notably, a Phase 3 clinical trial for cervical cancer (NCT02853604) is in a terminated state (for unknown reasons) [136]. There are also some other therapeutic cancer vaccines based on bacterial vectors that have been applied in clinical trials, such as for the treatment of pancreatic cancer (NCT01417000, NCT04589234) [137,138], breast cancer (NCT06631092) [139], and other solid tumors (Table 2).

### 2.2. Cellular Vaccines

#### 2.2.1. Dendritic Cell Vaccine

Dendritic cells are specialized antigen-presenting cells (APCs) that initiate effective tumor-specific immune responses by phagocytosis and processing of tumor antigens to T cells [147,148,149,150]. DC vaccine is obtained by sensitizing DC cells through tumor cell DNA, RNA, tumor cell lysate, tumor antigen protein/polypeptide, and other substances, and then using the powerful presentation function of DC cells to activate the patient’s T-cell immune response to achieve the purpose of tumor control [151]. At present, most DC vaccine products use patients’ autologous peripheral blood monocytes, which are prepared through in vitro expansion and antigen loading [152]. DC-based vaccines have been widely selected for immunotherapy. Currently, four DC vaccine products have been approved worldwide, including Hybricell (Genoa Biotechnologia), CreaVaxPCC (CreaGene), DCVax-Brain (Northwest Biotherp), and APCEDEN (APAC Biotech) for the treatment of melanoma, prostate cancer, kidney cancer, and glioma. In addition, based on the international clinical trial register platform (http://www.clinicaltrials.gov), according to the data shows that many based on DC vaccines have entered clinical trials, as a clinical trial has entered the stage 3 (NCT00045968), shows a good application prospect [153]. Most of the rest are Phase 1–2 clinical studies (Table 3). DC’s vaccines are mainly used in clinical trials to treat liver cancer, lung cancer (NCT02688673, NCT05195619) [154,155], breast cancer (NCT02063724, NCT02061423, NCT06435351, NCT04879888, NCT04105582) [156,157,158,159,160], melanoma (NCT01622933, NCT02301611, NCT01808820, NCT02678741, NCT01876212) [161,162,163,164,165], hematological malignancies (NCT02528682) [166], ovarian carcinoma (NCT05714306) [167], lung cancer (NCT02956551, NCT04147078, NCT03871205, NCT03371485) [168,169,170,171], glioblastoma (NCT03914768, NCT02771301, NCT04888611, NCT02529072, NCT02366728) [172,173,174,175,176], gastric cancer (NCT04567069, NCT04147078) [169,177], hepatocellular carcinoma (NCT04147078) [169], colorectal cancer (NCT04147078, NCT06545630, NCT03730948, NCT01885702) [169,178,179,180], and so on.

However, the clinical efficacy of DC vaccines is very limited, and recently, efforts have been made to develop new strategies to enhance the efficacy of DC vaccines. DC vaccine is developing towards individuation and precision, combination with other therapies, and integration with new technologies. In personalized and precise treatment, tumor-specific neoantigens with high immunogenicity can be predicted and screened according to the genetic information of patients’ tumor tissues so as to customize DC vaccines that are more in line with patients’ own characteristics, improve efficacy, and reduce side effects. In 2015, the first personalized neoantigen DC vaccine was tested in Phase 1 clinical trials (NCT00683670) [181]. They selected seven neoantigens from melanoma patients, loaded them into DC isolated from PBMC, and injected them intravenously three times to enhance the T-cell immune response. All three patients treated survived, and no adverse reactions were observed, demonstrating the safety and feasibility of the personalized neoantigen DC vaccine. Another personalized neoantigen DC vaccine trial was conducted in patients with advanced non-small-cell lung cancer (NCT02956551) [182]. Similarly, loading patients’ personalized neoantigens into DC isolated from the PBMC showed an overall 25% objective response rate and 75% disease control rate, with only mild and transient side effects observed. In addition, there are several other neoantigen DC vaccines for the treatment of ovarian cancer [183], breast cancer (NCT04879888, NCT04105582) [159,160], lung cancer (NCT04078269, NCT02956551, NCT03871205, NCT03205930) [168,170,184,185], liver cancer (NCT03674073) [186], and so on. As technology continues to advance, DC vaccines will focus more on individualized and precise strategies, with the deepening of research on the combined application of DC vaccines with immune checkpoint inhibitors, chemotherapy, and radiotherapy. Combination therapy will become the main trend of DC vaccine development. For example, a trial showed that the pp65 pulse DC vaccine combined with the chemotherapy drug temozolomide for glioma significantly extended overall survival (41.1 months) [187]. In another trial, an autologous EPHA2-targeted CAR-DC vaccine loaded with TP53 mutant peptide (TP53-EPHA-2-CAR-DC) combined with an anti-PD-1 antibody/anti-CTLA4 antibody is used in patients with locally advanced/metastatic solid tumors or relapsed/refractory lymphoma (NCT05631886) [188]. DC vaccine combined with immune checkpoint inhibitors can enhance the immune response of T cells. When combined with chemotherapy, more tumor antigens are released by the killing effect of chemotherapy drugs on tumor cells, and the DC vaccine can reactivate immune cells and improve the clearance effect of tumor cells. Based on the advantages of combination therapy, the synergies of DC vaccine and more therapies will continue to be explored and optimized to form better treatment options to overcome the limitations of tumor efficacy. In addition, with the development of nanotechnology, gene editing technology, cell engineering technology, etc., DC vaccines are also deeply integrated into these new technologies. For example, Mao et al. successfully delivered Cas9 mRNA and sgRNA to DC cells using LNP, achieving effective gene editing on DC cells [189]. By gene editing, the PD-L1 of DC cells was effectively knocked out, the activation and maturation of DC cells were enhanced, and the anti-tumor immune response mediated by T cells was improved, which significantly inhibited the growth of colon cancer in the tumor-bearing mouse model [189]. Another study showed that DC vaccines loaded with CircRNA encoding tumor antigens (FAPα and survivin) induced a stronger CD8^+^ T-cell response [190]. Moreover, its combination with gemcitabine significantly inhibited Panc02 tumor growth (89% inhibition rate) and extended survival in mice [190]. A more efficient antigen delivery vector based on nanotechnology was developed to improve the efficiency of antigen uptake and presentation by DC cells. And DC cells were modified by gene editing technology to enhance their immune activation ability.

Although DC vaccines show great potential in cancer immunotherapy, there are still challenges in preparation techniques, individual differences, off-target effects, delivery efficiency, and immunosuppressive microenvironments. However, with the advancement of technology, the continuous development of new cell separation and preparation technology, gene editing technology, efficient delivery systems, etc., will make the DC vaccine expected to become an important breakthrough in cancer immunotherapy.

**Table 3:** Clinical study of DC-based vaccines updated in recent 5 years.

Name	Cancer	ROA	Combination Therapy	NCI Number	Phase	Ref
Autologous dendritic cells pulsed with tumor lysate antigen	Glioblastoma	i.d.	/	NCT00045968	3	[153]
Autologous AdHER2-transduced dendritic cell vaccine	Breast cancer	i.d.	/	NCT01730118	1	[191]
Placental or tumor-derived heat shock protein gp96-induced DCs	Solid tumors	s.c. i.t.	/	NCT06477614	1	[192]
Autologous EphA2-targeting CAR-DC vaccine loaded with KRAS mutant peptide	Solid tumors	i.v.	Abraxane Cyclophosphamide Anti-PD-1 Anti-CTLA4	NCT05631899	1	[193]
Autologous EphA2-targeting CAR-DC vaccine loaded with TP53 mutant peptide	Solid tumors Lymphomas	i.v.	Abraxane Cyclophosphamide Anti-PD-1 Anti-CTLA4	NCT05631886	1	[188]
Immune-modified DC	Multiple myeloma Plasmacytoma	/	/	NCT06435910	1	[194]
Tumor antigen-pulsed DC	Esophageal squamous cell carcinoma	s.c.	/	NCT05317325	1	[195]
DC loaded with autologous tumor homogenate	Glioblastoma	i.d.	Temozolomide	NCT04523688	2	[196]
Autologous genetic-modification-free DC cells will be loaded with multiple tumor neoantigen peptides	Glioblastoma	s.c.	/	NCT06253234	1	[197]
Tumor antigen-sensitized DC	Melanoma Bladder cancer Colorectal cancer	s.c.	/	NCT05235607	1	[198]
Tumor neoantigen peptide vaccine/neoantigen-based DC	Advanced malignant solid tumors	s.c.	/	NCT05749627	Not applicable	[199]
Autologous DC loaded with patient-specific peptides or tumor lysates	Ovarian carcinoma	/	Cyclophosphamide	NCT05714306	1/2	[167]
Dendritic cell with tumor-associated antigen and patient-specific neoantigens	Ovarian cancer	/	/	NCT05270720	1	[200]
Tumor antigen-sensitized DC vaccine	Colorectal cancer	s.c.	/	NCT06545630	1	[178]
DC vaccines loaded with HPV 16/18 E6/E7 epitopes	Cervical intraepithelial neoplasia	/	/	NCT03870113	1	[201]
Anti-HER2/HER3 dendritic cell vaccine	Breast cancer	i.d.	Anti-PD-1	NCT04348747	2	[202]
Autologous dendritic cell-adenovirus p53 vaccine	Breast cancer	s.c.	/	NCT00082641	1/2	[203]
Total tumor RNA-pulsed DCs	Medulloblastoma	i.d.	Td vaccine autologous HSCs Anti-PD-1	NCT06514898	1	[204]
Immune-modified dendritic cells fused with leukemic cells (DCvac)	B-cell acute lymphoblastic leukemia	/	/	NCT05262673	1	[205]
Autologous dendritic cell	Prostate cancer	s.c.	/	NCT05533203	1	[206]
Immune-modified dendritic cell vaccine (DCvac)	T-cell acute lymphoblastic leukemia	/	/	NCT05277753	1	[207]
Peptide-pulsed autologous dendritic cell	Breast cancer	i.d.	/	NCT06195618	1	[208]
HER2-pulsed dendritic cell vaccine	HER2-positive breast cancer	i.d.	Anti-her2 Anti-PD-1 T-cell therapy	NCT05378464	1	[209]
Dendritic cell vaccine loaded with circular RNA encoding cryptic peptide	HER2-negative advanced breast cancer	i.d.	Anti-PD-1	NCT06530082	1	[210]
MIDRIX4-lung autologous DC vaccine	Non-small-cell lung cancer	i.v.	Antigen-specific DTH	NCT04082182	1	[211]
Autologous dendritic cell (ADC) vaccine	Small-cell lung cancer	i.d.	Carboplatin ADC vaccine	NCT04487756	1/2	[212]
TTRNA-DC vaccines with GM-CSF	Medulloblastoma	i.d.	Td vaccine autologous HSCs Anti-PD-1	NCT06514898	1	[204]
Tumor lysate-loaded autologous DC vaccine	Colorectal cancer	i.d.	/	NCT06522919	2	[213]
Autologous dendritic cell vaccine loaded with personalized peptides (PEP)	Pancreatic adenocarcinoma	s.c.	/	NCT04627246	1	[214]
HER-2-pulsed DC1	HER2-positive breast cancer	/	Anti-HER2 Anti-PD-1 Paclitaxel	NCT05325632	2	[215]
Allogeneic dendritic cell vaccine (DCP-001)	Ovarian cancer	/	/	NCT04739527	1	[216]
Autologous DC loaded with autologous tumor homogenate	Mesothelioma	i.d.	Anti-PD-1 Interleukin-2	NCT03546426	1	[217]
HER2 targeting autologous dendritic cell (AdHER2DC) vaccine	Endometrial cancer	i.d.	Anti-PD-1 N-803 Lenvatinib	NCT06253494	1/2	[218]
Autologous dendritic cell (DC) vaccine	Liver cancer	i.m.	Anti-PD-L1 Anti-VEGF RT Pneumococcal vaccine	NCT03942328	1/2	[219]
Multiple signals-loaded dendritic cells vaccine	Hepatocellular carcinoma	i.v.	Cyclophosphamide	NCT04317248	2	[220]
Autologous DCs pulsed with mutated peptides	Colorectal cancer	i.v.	/	NCT03730948	1	[179]
Autologous tumor blood vessel antigen (TBVA)-dendritic cell vaccine	Kidney cancer	i.d.	Cabozantinib	NCT05127824	2	[221]
Autologous DCs pulsed with genetically modified tumor cells or tumor-related antigens including neoantigens	Glioblastoma	i.d.	/	NCT03914768	1	[172]
CCL21	Non-small-cell lung cancer	i.m.	Anti-PD-1	NCT03546361	1	[222]
HER2-sensitized DC	Breast cancer	i.d.	/	NCT03630809	2	[223]
DC/multiple myeloma (MM) Fusion vaccine	Multiple myeloma	/	Anti-PD-1	NCT03782064	2	[224]
PDC*lung01	Non-small-cell lung cancer	s.c. i.v.	Anti-PD-1 Antifolate agents	NCT03970746	1/2	[225]
MG-7 antigen	Gastric cancer	s.c.	Anti-PD-1	NCT04567069	1/2	[177]
Autologous tumor lysate-pulsed dendritic cell vaccination	Glioblastoma	i.d.	Anti-PD-1 Poly-ICLC	NCT04201873	1	[226]
Tumor antigen-sensitized DC vaccine	Esophagus cancer	s.c.	/	NCT05023928	1	[227]
DC loaded with tri-antigens (WT1/TERT/survivin)	Acute myeloid leukemia	/	/	NCT05000801	Not applicable	[228]
DCs pulsed with GSC antigens (GSC-DCV)	Recurrent glioblastoma	s.c.	Anti-PD-1	NCT04888611	2	[174]
DC vaccine loaded with personalized peptides	Non-small-cell lung cancer	s.c.	Cyclophosphamide	NCT05195619	1	[155]
Neoantigen-loaded DC	Lung cancer	s.c.	/	NCT06329908	1	[229]
Autologous DCs loaded with multiple tumor neoantigen peptides	Glioblastoma multiforme of brain	i.d	Temozolomide	NCT04968366	1	[230]
Neoantigen	Hepatocellular carcinoma Colorectal cancer	i.d.	Anti-PD-1	NCT04912765	2	[231]
Neoantigen-derived dendritic cell	Refractory Tumor	s.c.	Anti-PD-1 Lenvatinib	NCT05767684	1	[232]
Neoantigen-primed DC	Gastric cancer Hepatocellular carcinoma Non-small-cell lung cancer Colon rectal cancer	s.c.	/	NCT04147078	1	[169]
Neoantigen-loaded DC	Non-small-cell lung cancer	s.c.	/	NCT03871205	1	[170]
Neoantigen dendritic cell	Breast cancer	Inguinal or axillary	Leukapheresis	NCT06435351	1	[158]
Tumor neoantigen-based vaccine FRAME-001	Non-small-cell lung cancer	s.c.	/	NCT04998474	2	[233]
Neoantigen-pulsed dendritic cell	Breast cancer	/	/	NCT04105582	1	[160]
Autologous neoantigen-targeted dendritic cell	Non-small-cell lung cancer	i.v.	Antigen-specific DTH	NCT04078269	1	[184]
Peptide-pulsed dendritic cell	Breast cancer	i.d.	/	NCT04879888	1	[159]
Neoantigen-pulsed dendritic cell	Breast cancer	/	/	NCT04105582	1	[160]
Personalized DC vaccine	Gastric cancer Hepatocellular carcinoma Non-small-cell lung cancer Colon rectal cancer	s.c.	/	NCT04147078	1	[169]
Neoantigen-loaded DC vaccine	Colorectal cancer	/	/	NCT01885702	1/2	[180]

**Abbreviation:** Intradermal injection (i.d.); subcutaneous injection (s.c.); intramuscular injection (i.m.); intravenous injection (i.v.).

#### 2.2.2. Tumor Cell Vaccine

Based on the characteristics of tumor cells carrying all tumor antigen information, the use of tumor cells as vaccines can provide adequate antigen information to the patient’s immune system, eliminating the need to identify the optimal antigen in a specific type of cancer, overcoming the problem of tumor antigen loss, and thus helping to better activate the anti-tumor immune response [234]. The types of tumor cell-based vaccines mainly include autologous tumor cell vaccines and allogeneic tumor cell vaccines.

##### Autologous Tumor Cell Vaccine

Autologous tumor cell vaccines belong to the category of personalized tumor therapeutic vaccines, which are mainly tumor cells obtained from patients, and the tumorigenic ability of tumor cells is removed by irradiation while retaining their immune activity. The treated tumor cells contain tumor-associated antigens, which can activate the patient’s own immune system after being transfused into the patient, prompting the body to produce a specific immune response against tumor cells and achieve the purpose of tumor treatment. It is worth noting that vaccines prepared by directly inactivating tumor cells have poor immunogenicity and very limited efficacy. To address the problem, current strategies are to genetically modify tumor cells and combine them with adjuvants or other therapies to improve the anti-tumor efficacy of vaccines. For example, Chang et al. developed a tumor cell vaccine that overexpresses mesothelin (a new tumor antigen for ovarian cancer), which, in combination with IL-12, significantly increased the proportion of mesothelin-specific T cells and prolonged mouse survival [235]. Currently, more research is on autologous tumor cell vaccines expressing GM-CSF (GVAX). In a variety of mouse tumor models, GVAX has been shown to promote the antigen presentation and activation of DC and has a good curative effect [236,237,238]. GVAX has been used in clinical trials for the treatment of pancreatic cancer (NCT02243371, NCT03153410, NCT00389610) [239,240,241], prostate cancer (NCT00140374) [242], and other tumors (Table 4). In addition, GVAX has also been selected for use in combination with other therapies to improve efficacy in clinical trials. For example, combination with nivolumab and ipilimumab for neuroblastoma (NCT04239040) [243], combination with Cyclophosphamide for Pancreatic Cancer (NCT01417000) [137], and combination with Pembrolizumab for Colorectal Cancer (NCT02981524) [244], and so on (Table 4). In Table 2, we systematically list the updated clinical studies of autologous tumor cell-based vaccines in the past five years.

##### Allogeneic Tumor Cell Vaccines

Allogeneic whole tumor cell vaccines usually contain two or three established human tumor cell lines to overcome the limitations of antigen source, molecular expression, and standardization of production and preparation of autologous tumor cell vaccines [245]. For allogeneic tumor cell vaccines, batch preparation of tumor cell lines or allogeneic cells can be achieved, and their cost is much lower than that of individualized vaccines. Moreover, allogeneic tumor cell vaccines usually carry multiple tumor-associated antigens, increasing the probability of covering more patients. For tumors with low immunogenicity, the immunogenicity of vaccines can be enhanced through genetic modification to demonstrate better therapeutic effects. Like VACCIMEL, a therapeutic cancer vaccine approved in Argentina composed of four allogeneic melanoma cell lines, effectively induces T-cell immune responses against neoantigens, allogeneic antigens, and tumor-associated antigens [246]. In a Phase 2 clinical study (NCT01729663), VACCIMEL demonstrated significant benefits in distant metastasis-free survival (DMFS) in patients with cutaneous melanoma receiving adjuvant therapy [247,248]. VACCIMEL combined with Bacillus Calmette–Guerin (BCG) and recombinant human granulocyte macrophage-colony stimulating factor (rhGM-CSF) adjuvants induced a strong specific immune response to TAA in patients and significantly enhanced the therapeutic effect of the vaccine [247,248,249,250]. Few allogeneic tumor cell therapeutic cancer vaccines have entered clinical research and are basically in the 1–2 stage, mainly used for the treatment of glioblastoma (NCT03360708, NCT04642937, NCT06305910, NCT04388033) [251,252,253,254].

**Table 4:** Clinical study of tumor cells-based vaccines updated in recent 5 years.

Target	Cancer	ROA	Combination Therapy	**NCI Number**	**Phase**	**Ref**
**Autologous tumor cellular vaccine**						
GM-CSF-secreting autologous neuroblastoma cell vaccine (GVAX)	Neuroblastoma	/	Anti-PD-1 Anti-CTLA4	NCT04239040	1	[243]
GVAX pancreas vaccine	Pancreatic cancer	i.d.	Anti-PD-1 CRS-207	NCT02243371	2	[239]
GVAX pancreas vaccine	Pancreatic cancer	i.d.	Anti-PD-1 IMC-CS4	NCT03153410	1	[240]
GVAX pancreas vaccine	Pancreatic cancer	i.d.	/	NCT00389610	2	[241]
GVAX pancreas vaccine	Pancreatic cancer	/	Anti-PD-1	NCT03161379	2	[255]
GVAX pancreas vaccine	Pancreatic cancer	/	Anti-PD-1 Anti-CTL4	NCT03190265	2	[256]
GVAX pancreas vaccine	Pancreatic cancer	i.d.	Cyclophosphamide FOLFIRINOX	NCT01595321	2	[257]
GVAX pancreas vaccine	Pancreatic cancer	/	Cyclophosphamide Anti-PD-1	NCT02648282	2	[258]
GM-CSF-secreting autologous leukemia cell vaccination (GVAX)	Myelodysplastic syndrome Acute myeloid leukemia Chronic myelomonocytic leukemia	i.d.	Chemotherapy	NCT01773395	2	[259]
GM-CSF-secreting leukemia cell vaccinations	Myeloid leukemia	s.c. or i.d.	/	NCT00426205	Not applicable	[260]
Allogeneic myeloma GM-CSF vaccine	Multiple myeloma	i.d.	Lenalidomide Pneumococcal vaccine	NCT03376477	2	[261]
GVAX colon vaccine	Colorectal cancer	i.d.	Anti-PD-1 CY	NCT02981524	2	[244]
Allogeneic colon cancer cell vaccine (GVAX)	Colorectal cancer	i.d.	CY SGI-110	NCT01966289	1	[262]
Colon GVAX	Colorectal cancer	/	CY	NCT00656123	1	[263]
Particle-delivered, allogeneic tumor cell lysate vaccine (PalloV-CC)	Colorectal cancer	i.d.	/	NCT03827967	1	
GVAX prostate cancer vaccine	Prostate cancer	i.d.	CY	NCT01696877	1/2	[264]
Autologous tumor cellular vaccine	Prostate cancer	i.d.		NCT06636682	2	
GVAX	Melanoma Sarcoma/renal cell carcinoma	/	/	NCT00258687	1	[95]
Personalized neoantigen cancer vaccine	Kidney cancer	s.c.		NCT02950766	1	
Autologous breast cancer cells engineered to secrete GM-CSF	Breast cancer	/	/	NCT00317603	1	[265]
Autologous breast cancer cells engineered to secrete GM-CSF	Breast cancer	/	/	NCT00880464	1	[266]
GRT-C901, GRT-R902	Non-small-cell lung cancer Colorectal cancer Gastroesophageal adenocarcinoma Urothelial carcinoma	/	Anti-PD-1 Anti-CTL4	NCT03639714	1/2	[267]
GRT-C901, GRT-R902	Non-small-cell lung cancer Colorectal cancer Gastroesophageal adenocarcinoma Urothelial carcinoma	/	Anti-PD-1 Anti-CTL4	NCT03639714	1/2	[267]
OVM-200	Prostate cancer Lung cancer Ovarian cancer	/	/	NCT05104515	1	[268]
**Allogeneic tumor cell vaccine**						
Therapeutic vaccine (ACIT-1)	Pancreatic cancer Other cancer	/	/	NCT03096093	1/2	[269]
Malignant glioma tumor lysate-pulsed	Glioblastoma	s.c.	Autologous dendritic cell	NCT03360708	1	[251]
Allogeneic tumor lysate vaccine (GBM6-AD)	Glioblastoma	/	CD200AR-L imiquimod	NCT04642937	1	[252]
Allogeneic tumor lysate vaccine (GBM6-AD)	Glioblastoma	/	CD200AR-L imiquimod	NCT06305910	1	[253]
DC/tumor cell fusion vaccine	Glioblastoma	/	Anti-CTLA4	NCT04388033	1/2	[254]
Therapeutic vaccine (ACIT-1)	Pancreatic cancer Other cancer	/	/	NCT03096093	1/2	[269]

**Abbreviation:** Intradermal injection (i.d.); subcutaneous injection (s.c.).

### 2.3. Peptide Vaccines

Peptide tumor vaccine uses synthetic peptide fragments as antigens to stimulate the body to produce an anti-tumor immune response. Peptide vaccines have been paid more and more attention to because they are completely synthetic, with high safety (no complete pathogen), high specificity, flexible design, and low cost [151]. Currently, three peptide vaccines are marketed worldwide, vitespen, EGF-P64K, and racotumomab, for the treatment of glioma, renal cell carcinoma, cervical cancer, and non-small-cell lung cancer. Furthermore, many peptide tumor vaccines are in the clinical trial stage. We summarize the clinical research progress of the updated peptide tumor vaccines in the past five years in Table 5.

Traditional peptide tumor vaccine has some defects, such as poor immunogenicity, low efficacy, and short half-life, which affect its therapeutic effect in clinical application. To address the very limited efficacy of peptide tumor vaccines, many studies have focused on screening highly specific neoantigen peptides, optimizing immune-stimulating adjuvants, developing more effective delivery systems, and exploring combination therapy strategies to enhance immune response and tumor suppression.

Personalized neoantigen vaccines have been regarded as an effective method for inducing, enhancing, and diversifying anti-tumor T-cell responses [270]. For example, a personalized neoantigen polypeptide vaccine demonstrated clinical feasibility, safety, and immunogenicity for the first time in a Phase I clinical trial in melanoma patients [271]. The vaccine can target up to 20 predicted individual tumor neoantigens, increasing the number of antigen-specific T cells, such as induced CD4^+^ and CD8^+^ T cells targeting 58 (60%) and 15 (16%) of 97 unique neoantigens, respectively [271]. It is well known that there is still no better treatment method for patients with glioblastoma. After standard treatment, there are often problems of recurrence, poor treatment effect, and limited survival period. In a study, through somatic mutation analysis of the tumors of 173 glioblastoma patients, personalized peptide vaccines targeting tumor-specific neoantigens were produced [272]. Among the blood samples of 97 (90%) monitored patients, vaccine-induced immune responses to at least one vaccination peptide were detected in 87 cases [272]. Most patients developed persistent specific T-cell responses, and the survival period (53 months) of patients with multiple vaccine-induced T-cell responses was significantly longer than that of patients with no or low induced responses (27 months) [272]. This study demonstrated the feasibility of individualized neoantigen-targeted peptide vaccines, which provide promising potential treatment options for the treatment of glioblastoma patients [272]. With advances in high-throughput sequencing technology, genomics, synthesis technology, and data science, rapid screening, optimization, and preparation of personalized antigens can be achieved. Based on the optimization of tumor neoantigen personalized vaccine design strategy, many related types of vaccines have been used in clinical trials to treat melanoma (NCT05098210, NCT01970358, NCT03929029), lung cancer (NCT04397926, NCT02897765, NCT04487093, NCT03380871), and other cancers (Table 5).

GM-CSF is a powerful immune adjuvant that can increase the maturation and function of dendritic cells, thereby enhancing antigen presentation [273]. In a preclinical study, local injection of GM-CSF, IL-2, and HPV16 E7 peptide enhanced vaccine-specific immune responses and induced higher CTL and cytokine release without increasing immunosuppressive Treg cells, more effectively inhibiting the growth of TC-1 tumor cells [274]. In a clinical study (Phase 2, NCT02636582), a peptide vaccine composed of HER2-derived MHC Class I peptide E75 (nelipepimut-S, NPS) combined with GM-CSF adjuvant in the treatment of patients with ductal carcinoma in situ (DCIS) showed good vaccine tolerance and relatively good safety [275]. Moreover, vaccination enhances the NPS-specific cytotoxic T lymphocyte (CTL) response, and the increase in the proportion of specific T cells produced in the NPS + GM-CSF group exceeds that in the NPS alone treatment group [275]. Cytosine-guanosine oligodeoxynucleotide (CpG) also is a strong adjuvant that promotes the production of pro-inflammatory cytokines, stimulates DC and B-cell activation, and induces and enhances Th1 type immune response [276,277,278,279,280,281,282,283]. In a study, all eight melanoma patients with HLA-A2^+^ showed rapid and intense antigen-specific T-cell responses after receiving treatment with a low-dose CpG 7909 combined with melanoma antigen A analog peptide and incomplete Freund’s adjuvant vaccine [284]. The number of antigen-specific T cells produced by patients in the CPG treatment group was significantly higher than that in the CpG treatment group [284]. The mechanism is achieved by the increased T cells recognizing and killing melanoma cells in an antigen-specific manner [284]. Other different antigen-peptide vaccines combined with adjuvants have also been used in clinical trials to treat melanoma (NCT00471471, NCT00112242, NCT00112229, NCT05098210) [284,285,286,287,288,289], breast cancer (NCT02593227, NCT05232916, NCT03012100, NCT05098210) [289,290,291,292], lung cancer (NCT02818426, NCT03380871, NCT01949701, NCT06472245) [293,294,295,296], glioma (NCT02193347) [297], pancreatic cancer (NCT03645148, NCT05013216) [291,298], and other cancers (Table 5).

Although neoantigen peptide vaccines have great potential in tumor immunotherapy, their progress in clinical trials has been hindered due to the limitations of antigen cell uptake and cross-presentation. Based on the development of delivery technology, nanovaccines co-delivered with neoantigens and adjuvants have been regarded as a very promising approach to personalized cancer immunotherapy, with encouraging results in several preclinical animal models [299,300,301,302,303]. For example, Moon et al. designed a high-density lipoprotein-mimicking nanodiscs delivery strategy that co-delivered neo-epitopes and the adjuvant CPG, significantly improved the delivery efficiency of antigen in vivo, improving delivery efficiency and enhancing the frequency of neoantigen-specific CD8α+ cytotoxic T lymphocytes (47 times higher), and effectively inhibiting the tumor growth of B16F10 and MC38 tumor-bearing mice [300]. In addition, some nanovaccines based on co-delivery antigens and adjuvants have also been used to treat melanoma [302,304], breast cancer, colon cancer [302,303,305,306], liver cancer [307], lung cancer [308], gliomas [309], etc. However, neoantigen and adjuvant tumor vaccines loaded based on new delivery technologies are still mainly preclinical studies.

In addition to strategies such as optimizing adjuvants and developing new delivery systems to enable peptide tumor vaccines, combination with other therapies is also an important approach. In a Phase 2 clinical trial (NCT02455557), the peptide vaccine SurVaxM plus temozolomide in glioblastoma patients showed a good safety profile, a strong antigen-specific CD8^+^ T cells response, and 95.2% of patients remained progression-free six months after diagnosis [310]. Glioblastoma is a very-high-mortality tumor, and in clinical trials evaluating standard radiation and chemotherapy, the median survival of most patients was only 14.6 to 16.0 months. It is exciting to see that SurVaxM plus temozolomide treatment significantly improved the median overall survival of patients (25.9 months) [310,311,312]. For patients with metastatic melanoma, improving overall survival has been a formidable challenge to overcome. In a Phase 3 clinical trial (NCT00094653), the median overall survival of patients with metastatic melanoma treated with glycoprotein 100 (gp100) peptide vaccine alone was 6.4 months. To improve survival, the combination of the gp100 peptide vaccine and ipilimumab (an anti-CTLA-4 antibody) showed good clinical expectations, extending survival to 10.0 months [313]. In another Phase 1b clinical study (NCT02897765), NEO-PV-01, a neoantigen vaccine tailored to a patient’s tumor gene mutation, was shown to be effective in combination with PD-1 antibodies in patients with advanced melanoma, non-small-cell lung cancer, and bladder cancer [314]. In addition, some other clinical studies related to peptide tumor vaccines combined with other therapies in recent years are summarized in Table 5.

At present, the research progress of peptide tumor vaccines mainly revolves around the research of personalized peptide vaccines, tumor-associated antigens, and adjuvants (such as TLR agonists, STING agonists, cytokines) and delivery systems (such as nanoparticles, liposomes, and other novel delivery systems) to enhance immune response. With the development of sequencing technology and bioinformatics, new adjuvants, new delivery systems, and other technologies, the trend of personalized and combination therapy of peptide vaccines is developing. However, peptide tumor vaccines also face many challenges, such as poor immunogenicity, tumor immunosuppressive microenvironment, individual differences, and antigen escape. It is believed that with the innovation and development of technology, peptide tumor vaccines will definitely achieve accurate vaccine design by combining multiple omics and exploring multi-mode combined treatment schemes to improve the clinical effect of vaccines.

**Table 5:** Clinical study of peptide tumor vaccines updated in recent 5 years.

Target Antigen	Adjuvant	Cancer	RoA	Combination Therapy	NCI Number	Phase	Ref
GP96 heat shock protein–peptide complex	/	Liver cancer	/	/	NCT04206254	2/3	[315]
Tumor antigen peptides	/	Liver cancer	s.c.	/	NCT05059821	1	[316]
ELI-002 7P	/	Solid tumors	s.c.	/	NCT05726864	1/2	[317]
ELI-002 2P (Amph modified KRAS peptides, Amph-G12D and Amph-G12R admixed with admixed Amph-CpG-7909)	/	Kirsten rat sarcoma (KRAS) mutated pancreatic ductal adenocarcinoma and other solid tumors	s.c.	/	NCT04853017	1	[318]
Neoantigen peptides vaccine	/	Non-small-cell lung cancer	s.c.	/	NCT04397926	1	[319]
ARG1 peptides	Montanide ISA-51	Solid tumors	s.c.	/	NCT03689192	1	[320]
HLA-A*2402 or A*0201 restricted peptides	Montanide ISA 51	Solid tumors	s.c.	/	NCT01949688	1/2	[321]
HLA-A*0201restricted URLC10 peptides	Montanide ISA 51	Non-small-cell lung cancer	s.c.	/	NCT01949701	1/2	[295]
Two peptides called UCP2 and UCP4 derived from telomerase	Montanide ISA 51	Non-small-cell lung cancer	/	/	NCT02818426	1/2	[293]
OSE2101	Montanide ISA 51	Non-small-cell lung cancer	s.c.	/	NCT06472245	3	[296]
Melan-A-ELA + NY-ESO-1b + MAGE-A10 peptide + Montanide + CpG	Montanide ISA 51	Melanoma	/	/	NCT00112242	1	[287]
PD-L1 peptide	Montanide ISA 51	Multiple myeloma	s.c.	/	NCT03042793	1	[322]
IDH1 peptide vaccine	GM-CSF	Glioma	i.d.	/	NCT02193347	1	[297]
FRα peptide	GM-CSF	Breast cancer	i.d.	/	NCT02593227	2	[290]
HER2/neu peptide GLSI-100 (GP2 + GM-CSF)	GM-CSF	Breast cancer	i.d.	/	NCT05232916	3	[291]
Multi-epitope folate receptor alpha peptide	GM-CSF	Breast cancer	i.d.	/	NCT03012100	2	[292]
Neoantigen peptides	GM-CSF	Solid tumors	/	/	NCT03662815	1	[323]
Neoantigen peptides	GM-CSF	Pancreatic cancer	/	/	NCT03645148	1	[324]
Mutant Kirsten rat sarcoma (KRAS)-targeted long peptide	Poly-ICLC	Pancreatic cancer	/	/	NCT05013216	1	[298]
NEO-PV-01 (personalized neoantigen)	Poly-ICLC	Melanoma Non-small-cell lung cancer	s.c.	/	NCT02897765	1	[314,325]
Neoantigen peptides	Poly-ICLC	Breast cancer Melanoma	i.m.	/	NCT05098210	1	[289]
Neoantigen peptides	Poly-ICLC	Melanoma	/	/	NCT01970358	1	[326]
AE37 peptide vaccine	/	Breast cancer	i.d.	Anti-PD-1	NCT04024800	2	[327]
OTSGC-A24	/	Gastric cancer	s.c.	Anti-PD-1 + Anti-CTLA4	NCT03784040	1	[328]
Synthetic tumor-associated peptide	/	Pancreatic cancer Colorectal cancer	s.c.	Anti-PD-1 Anti-PD-1 + APX005M	NCT02600949	1	[329]
Neoantigen peptide	/	Non-small-cell lung cancer	s.c.	EGFR-TKI Anti-angiogenic	NCT04487093	1	[330]
Liposomal HPV-16 E6/E7 multi-peptide vaccine PDS0101	/	HPV-oropharyngeal squamous cell carcinoma	s.c.	Anti-PD-1	NCT05232851	1/2	[331]
Neoantigen heat shock protein vaccine (rHSC-DIPGVax)	/	Glioma	/	Anti-PD-1 + Anti-CTLA4	NCT04943848	1	[332]
Survivin long peptide (SurVaxM)	Montanide ISA 51	Neuroendocrine tumors	s.c.	Octreotide acetate	NCT03879694	1	[333]
UCP2 and UCP4 derived from telomerase (UCPVax)	Montanide ISA 51	Papillomavirus-positive cancers	s.c.	Anti-PD-L1	NCT03946358	2	[334]
NPMW-peptide vaccine	Montanide ISA 51	Myelodysplastic syndrome Acute myeloid leukemia	/	Anti-PD-L1	NCT02750995	1	[335]
Personalized multi-peptide vaccine cocktails	XS15, Montanide ISA 51	Cancer	s.c.	TLR1/2 ligand XS15	NCT05014607		[336]
MVF-HER-2 (597–626) and MVF-HER-2 (266–296)	Montanide ISA 720	Advanced solid tumors	i.m.	/	NCT06414733	1	[337]
Neoantigen peptides vaccine	Montanide ISA 51 + Poly-ICLC	Melanoma	/	Anti-PD-1+ Anti-CTLA4	NCT03929029	1	[338]
PVX-410 (contains four synthetic peptides)	Poly- ICLC	Smoldering multiple myeloma	s.c.	Citarinostat + Lenalidomide	NCT02886065	1	[339]
NEO-PV-01	Poly-ICLC	Non-small-cell lung cancer	s.c.	Anti-PD-1 + Chemotherapy	NCT03380871	1	[294]
Pooled mutant KRAS-targeted long peptide vaccine	Poly-ICLC	Colorectal cancer Pancreatic cancer	/	Anti-PD-1 + Anti-CTLA4	NCT04117087	1	[340]
DNAJB1-PRKACA fusion kinase peptide	Poly-ICLC	Liver cancer	/	Anti-PD-1 + Anti-CTLA4	NCT04248569	1	[341]
Personalized multi-peptide	Poly-ICLC	Prostate cancer	/	CDX-301	NCT05010200	1	[342]
KRAS peptide vaccine	Poly-ICLC	Non-small-cell lung cancer	/	Anti-PD-1+ Anti-CTLA4	NCT05254184	1	[343]
MUC1 peptide vaccine	Poly-ICLC	Ductal carcinoma in situ	s.c.	Aromatase inhibitor	NCT06218303	1	[344]
Galinpepimut-S	GM-CSF	Acute myelogenous leukemia Ovarian cancer Colorectal cancer Breast cancer Small-cell lung cancer	/	Anti-PD-1	NCT03761914	1/2	[345]
Neoantigen peptide	GM-CFS	Solid tumors	i.v.	Anti-PD-1	NCT05269381	1/2	[346]

**Abbreviation:** Intradermal injection (i.d.); subcutaneous injection (s.c.); intramuscular injection (i.m.); intravenous injection (i.v.).

### 2.4. Nucleic Acid Vaccines

#### 2.4.1. DNA Tumor Vaccine

In cancer therapy, DNA cancer vaccines are considered to be a very attractive and promising means, with advantages such as low cost, cell-independent production, durable immune response, and potential to target multiple neoantigens [151,347]. Of course, there are also defects of host gene integration risk, autoimmune reaction risk, and low transfection efficiency [151]. In order to improve efficacy and safety, different strategies are being used to optimize and improve DNA vaccines. To improve efficacy and safety, efforts have been made to optimize and improve DNA vaccines through different strategies, such as inserting optimized optimal antigens.

Previous studies have shown that selecting and inserting the optimal antigen for plasmid DNA is an ideal way to enhance vaccine immunogenicity and induce a broad immune response, which can overcome problems associated with antigen loss, modification, and tolerance [347]. DNA vaccine construction based on enhanced immunogenicity strategy mainly includes chimeric DNA vaccine, neoantigen DNA vaccine, and polypeptide DNA vaccine. Chimeric DNA vaccines are heterologous antigenic vaccines that encode proteins or peptides from different species, and their sequences have significant homology with the self-ortholog [348,349]. Since the homologous and natural protein sequences are only similar but not identical, this helps to circumvent immune tolerance while maintaining homology that can be recognized by T cells to enhance the potential immunogenic response [348,349,350]. Previous studies have shown that xenoantigens are more effective than autoantigens [350,351]. For example, xenogeneic DNA vaccines targeting human tyrosinase were approved to treat canine melanoma [349], Xenovaccines designed with rhesus CEA (rhCEA) as the immunogen against human carcinoembryonic antigen (hCEACAM-5 or commonly hCEA) can activate CD4^+^ T cells and autoreactive CD8^+^ T cells, and produce high-titer antibodies against hCEA and have significant anti-tumor effects. Furthermore, codon-optimized RhCEA cDNA (rhCEAopt) was demonstrated to have higher immune reactivity than hCEAopt in mice [352], Chimeric rat/human HER2 efficiently circumvents HER2 tolerance in cancer patients [353]. DNA vaccines encoding mouse/human chimeric proteins induce a better immune response against Erbb-2 tumors in mice [354]. DNA xenovaccines have shown encouraging results in a clinical trial for melanoma [355,356]. Neoantigen vaccines are selected to express antigens specifically in tumor tissue, which overcomes the problem of immune tolerance deficiencies and side effects [357,358]. For example, Li et al.’s optimized polypeptide neoantigen DNA vaccine induced strong neoantigen-specific T-cell responses in preclinical mouse breast cancer models E0771 and 4T1 and combined with anti-PD-L1 antibody effectively inhibited the growth of E0771 tumors and maintained anti-tumor immunity [359].

In clinical trials, DNA vaccines are being used to treat liver cancer (NCT04251117) [360], melanoma (NCT03655756) [361], breast cancer (NCT05455658, NCT04246671, NCT02780401) [362,363,364], non-melanoma skin cancers (NCT04160065) [365], glioblastoma (NCT04015700, NCT05743595) [366,367], prostate cancer (NCT03532217, NCT03600350, NCT04090528) [368,369,370], and other cancers (Table 6), most of which were in the Phase 1–2 clinical research stage. Despite efforts to improve the delivery efficiency of DNA vaccines, their immunogenicity in clinical trials remains limited. Therefore, people still need to continue exploring more strategies to enhance the immunogenicity of DNA vaccines, such as optimizing DNA vaccine vectors, combining cytokine adjuvants, and exploring innovative delivery methods, etc. [371].

#### 2.4.2. RNA Vaccine

With the outbreak of COVID-19, the urgent use of two mRNA vaccines has brought mRNA vaccines back into the spotlight. Like DNA, mRNA can encode an unlimited number of proteins and peptides. However, mRNA vaccines have several irreplaceable advantages, such as no risk of gene integration, repeatability, coding flexibility and versatility, short production cycle, and low cost [383,384,385]. Based on the editable flexibility of mRNA vaccines, they can encode tumor antigens as tumor antigen vaccines, cytokines for immunotherapy, tumor suppressors to inhibit tumor development, chimeric antigen receptors for engineered T-cell therapy, and genomic proteins for gene therapy. In this section, we will focus on describing the progress of mRNA therapeutic cancer vaccines in clinical studies (Table 7).

Because mRNA is easily degraded by RNases, there is little research on naked mRNA vaccines, and the main focus is on the application of delivery systems to deliver mRNA into the body. Currently, the strategies for delivering mRNA mainly include protamine, cationic liposomes, and LNP. Protamin-coated mRNA vaccines use the positive charge of protamine to form a complex with negatively charged mRNA to avoid mRNA degradation [386]. For example, in a Phase 1/2 clinical trial (NCT00204607), subcutaneous injection of protamine-stabilized mRNAs encoding Melan-A, Tyrosinase, gp100, Mage-A1, Mage-A3, and survivin in 21 patients with metastatic melanoma demonstrated that the vaccine was safe with no grade II adverse events and activated the immune response. The frequency of Foxp3^+^/CD4^+^ immunosuppressive cells was significantly decreased, and some patient-specific T cells were increased [387]. The strategy of delivering mRNA into the body by means of an mRNA-lipoplex complex formed by cationic liposomes with negatively charged mRNA is currently studied and paid more attention. For example, BNT-111, developed by BioNtech Company, is a mRNA-lipoplex vaccine designed for melanoma antigen (MAGE-A3, NY-ESO-1, TPTE, Tyrosinase). In a Phase II clinical study (NCT02410733), BNT-111 demonstrated good clinical benefits, with 75% of patients producing an anti-tumor immune response [388]. Lipid nanoparticles are currently very mature mRNA delivery platforms, mainly composed of lipids, phospholipids, and cholesterol [389,390]. mRNA-4157, developed by Moderna, is an mRNA vaccine encoding 34 tumor neoantigens and wrapped with LNP. It is also the fastest-growing mRNA therapeutic cancer vaccine (Phase 3, NCT06077760, NCT05933577) [391,392]. In a 2b clinical trial (NCT03897881), the recurrence-free survival of melanoma patients treated with mRNA-4157 combined with pembrolizumab was longer than that of pembrolizumab monotherapy (79% versus 62%). And it has relatively good safety, with no mRNA-4157-related grade 4/5 events [393]. Furthermore, another Phase 1 clinical study (NCT03313778) on non-small-cell lung cancer or melanoma evaluated the safety, tolerability, and immunogenicity of mRNA-4157 [394]. The results showed that no patient had grade 4/5 adverse events or dose-limiting toxicity [394]. mRNA-4157 alone can induce consistent new generation and enhance the pre-existing T-cell response to targeted neoantigens, and the combination therapy induces sustained neoantigen-specific T-cell responses and the expansion of cytotoxic CD8 and CD4 T cells [394]. The relevant clinical studies of mRNA-4157 have demonstrated the great potential and significance of mRNA-4157 as an adjuvant monotherapy or in combination with other therapies.

There are also many other mRNA therapeutic cancer vaccines in the clinical stage, which are used to treat melanoma (NCT04526899, NCT03897881) [395,396], liver cancer (NCT05981066, NCT05738447, NCT05761717) [397,398,399], lung cancer (NCT03164772, NCT06735508) [400,401], pancreatic cancer (NCT06326736, NCT06577532, NCT06496373, NCT06156267, NCT06353646, NCT04161755) [402,403,404,405,406,407], and other cancers (Table 7). In addition, many studies are exploring the design and application of novel mRNA, such as self-amplified mRNA (saRNA), trans-amplified mRNA (taRNA), and circular mRNA (circRNA), as well as the long-term preservation means of mRNA nanoparticles, drug delivery routes, and organ-selective precision translation [383]. These explorations are expected to enable mRNA-based anti-cancer therapies to further cover various types of cancer and benefit a broad population of patients.

**Table 7:** Clinical study of mRNA vaccines updated in recent 5 years.

Name	Cancer	ROA	Combination Therapy	NCI Number	Phase	Ref
NY-ESO-1, MAGE-A3, tyrosinase, and TPTE	Melanoma	i.v.	Anti-PD-1	NCT04526899	2	[395]
mRNA-4157	Melanoma	/	Anti-PD-1	NCT03897881	2	[396]
mRNA-4157	Melanoma	i.m.	Anti-PD-1	NCT05933577	3	[392]
mRNA-4157	Cutaneous squamous cell carcinoma	i.m.	Anti-PD-1	NCT06295809	2/3	[408]
mRNA-4157	Renal cell carcinoma	i.m.	Anti-PD-1	NCT06307431	2	[409]
HBV mRNA vaccine	Liver cancer	i.m.	/	NCT05738447	1	[398]
Neoantigen mRNA vaccine (ABOR2014/IPM511)	Liver cancer	i.m.	/	NCT05981066	Not applicable	[397]
Neoantigen mRNA personalized cancer vaccine	Liver cancer	s.c.	Anti-PD-1	NCT05761717	Not applicable	[399]
mRNA-4157	Non-small-cell lung cancer	i.m.	Anti-PD-1	NCT06077760	3	[391]
BI 1361849 mRNA vaccine comprises 6 drug product components (MUC1, survivin, NY-ESO-1, 5T4, MAGE-C2, MAGE-C1)	Non-small-cell lung cancer	i.d.	Anti-PD-L1 Anti-CTLA4	NCT03164772	1/2	[400]
BI 1361849 mRNA vaccine comprises 6 drug product components	Non-small-cell lung cancer	i.d.	Anti-PD-L1 Anti-CTLA4	NCT03164772	1/2	[400]
Neoantigen mRNA vaccines	Non-small-cell lung cancer	/	Anti-PD-L1	NCT06735508	1	[401]
Fixed combination of shared cancer antigens	Head and neck cancer	i.v.	Anti-PD-L1	NCT04534205	2	[410]
EBV mRNA vaccine	Malignant tumors	i.m.	/	NCT05714748	1	[411]
Personalized neoantigen mRNA vaccine iNeo-Vac-R01	Digestive system neoplasms	s.c.	/	NCT06019702	1	[412]
mRNA neoantigen vaccine iNeo-Vac-R01	Digestive system neoplasms	s.c.	/	NCT06026774	1	[413]
Neoantigen mRNA vaccines	Digestive system neoplasms	s.c.	/	NCT03468244	Not applicable	[414]
Neoantigen mRNA vaccines iNeo-Vac-R01	Neoantigen mRNA vaccines	s.c.	/	NCT06026800	1	[415]
Neoantigen mRNA	Esophageal cancer Non-small-cell lung cancer	s.c.	/	NCT03908671	Not applicable	[416]
mRNA neoantigen vaccine (mRNA-0523-L001)	Endocrine tumor	i.m.	/	NCT06141369	Not applicable	[417]
Neoantigen mRNA vaccines	Pancreatic cancer	/	Gemcitabine + Abraxane	NCT06326736	1	[402]
KRAS neoantigen mRNA vaccine (ABO2102)	Pancreatic cancer	i.m.	Anti-PD-1	NCT06577532	1	[403]
Neoantigen mRNA vaccines	Pancreatic cancer	/	Anti-PD-1	NCT06496373	1	[404]
Neoantigen mRNA vaccines	Pancreatic cancer	/	Anti-PD-L1	NCT06156267	1	[405]
XH001 (neoantigen cancer vaccine)	Pancreatic cancer	/	Anti-CTLA4 + Chemotherapy	NCT06353646	Not applicable	[406]
Personalized neoantigen tumor vaccines	Pancreatic cancer	/	Anti-PD-L1	NCT04161755	1	[407]
mRNA 2752	Carcinoma	Intralesional (IL)	Anti-PD-1	NCT02872025	1	[418]
mRNA-4157	Solid tumors	i.m.	Anti-PD-1	NCT03313778	1	[419]
Neoantigen mRNA vaccine	Solid tumors	i.t.	/	NCT06195384	1	[420]
Neoantigen mRNA vaccine SW1115C3	Solid tumors	s.c.	/	NCT05198752	1	[421]
Neoantigen mRNA personalized cancer vaccine	Solid tumors	s.c.	Anti-PD-1	NCT05949775	Not applicable	[422]
Neoantigen mRNA vaccines	Solid tumors	i.m.	Anti-PD-1	NCT06497010	1	[423]
XH001 (neoantigen cancer vaccine)	Solid tumors	/	Anti-PD-1	NCT05940181	Not applicable	[424]
Individualized neoantigen vaccine mRNA-4157	Solid tumors	i.m.	Anti-PD-1	NCT03313778	1	[419]
IL-7, IL-12 BNT152 + 153	Solid tumors	i.v.	/	NCT04710043	1	[425]
mRNA-2752, a lipid nanoparticle encapsulating mRNAs encoding human OX40L, IL-23, and IL-36γ	Solid tumors	i.m.	Anti-PD-1	NCT03739931	1	[426]
IL-12 MEDI1191	Solid tumors	i.t.	/	NCT03946800	1	[427]

**Abbreviation:** Intradermal injection (i.d.); subcutaneous injection (s.c.); intramuscular injection (i.m.); intravenous injection (i.v.); intertumoral injection (i.t.).

## 3. Challenges and Trends in Therapeutic Vaccines

Immunotherapy is an effective means of treatment following drug, surgery, and radiotherapy, and its clinical role is increasingly prominent. Therapeutic cancer vaccines, as one of the main methods of immunotherapy, have become a new growth point of biomedicine with broad industrial prospects in the post-COVID-19 era. Major international vaccine companies [such as BioNTech SE, CureVac AG, Moderna TX, Merck Sharp & Dohme Corp] have laid out research and development pipelines to promote their clinical transformation.

For immunotherapy strategies, the anti-tumor process mainly consists of three links: effective antigen release, immune activation, and tumor killing. These links complement each other, and none can be missing. Vaccines developed in the past often had many deficiencies, resulting in slow development and limited therapeutic effects. Tumor antigens are the key factors that initiate the anti-tumor immune response and also the crucial link that tumor therapeutic vaccines need to address. For immune checkpoint inhibitors (PD-1/PD-L1 antibodies) and CAR-T cell therapy, they address the aspect of “tumor killing”, and the treatment process faces the problem of immune tolerance. Recently, the development of gene sequencing technology and bioinformatics has enabled more precise identification of specific gene mutations and neoantigens in patients’ tumor cells, thereby promoting the increasing precision of antigens. Therapeutic cancer vaccines will be highly customized based on the individual tumor antigen characteristics of each patient to enhance the vaccine’s specificity and efficacy. However, individualized vaccines based on tumor neoantigens still face many challenges, such as tumor heterogeneity, immunogenicity, and how to scientifically and reasonably design and validate clinical trial protocols, etc. Tumor cells are highly heterogeneous, so the antigen expression of tumor cells in different patients may vary. In practical applications, it is difficult to find a universal tumor antigen for vaccine design, which also increases the difficulty for vaccines to cover all tumor cells. Furthermore, tumors progress rapidly and are prone to mutation, which requires a fast process from antigen sequencing and screening to design, undoubtedly putting pressure on vaccine production. The issue of immunogenicity is that tumor antigens usually have weak immunogenicity and are difficult to stimulate a strong immune response, and the immune system in the body may develop immune tolerance to tumor antigens, resulting in poor vaccine efficacy. For instance, a personalized neoantigen cancer vaccine based on mRNA was terminated due to its clinical efficacy failing to meet expectations (Phase 1/2, NCT03480152) [428]. Therefore, in order to solve the problems in the immune activation part, many studies have been dedicated to developing more effective adjuvants and antigen presentation techniques to enhance the immunogenicity of tumor antigens and break immune tolerance. In particular, peptide vaccines are greatly affected by adjuvants. Many studies have proved the favorable effects of adjuvants on vaccines, such as adjuvants GM-CSF, CpG, etc. Moreover, adjuvants are also developing towards compound adjuvants, taking advantage of their respective strengths and complementing each other’s weaknesses. It is believed that with the advantages of new adjuvants and compound adjuvants, the efficacy of vaccines is expected to be continuously improved in the future. In addition to adjuvants, new delivery technologies have also been a key focus area in recent years. So far, delivery carriers include viruses, bacteria, cells, lnp, etc. For viral vector vaccines, the development of genetic engineering technology has made the modification of viral vectors safer, more precise, and more efficient, which can improve the targeting, immunogenicity, and safety of the vectors. For instance, by designing and optimizing the structure and function of viruses through gene editing, viral vectors can infect tumor cells more specifically while reducing their impact on normal cells. There are already many therapeutic cancer vaccines based on viral vectors in the clinical research stage (Table 1). However, such vaccines still face many challenges, mainly the accompanying issues related to immunogenicity and safety. For example, the safety issues brought about by potential inserted gene mutations, the neutralizing antibodies produced by antiviral responses reduce the therapeutic effect and the production complexity problems such as the high cost of large-scale production and quality control of viral vectors. Delivery technologies such as cell and LNP are all aimed at improving the delivery efficiency of antigens into tumors, increasing the effective concentration of antigens within the tumor to enhance the activation of immune responses, and simultaneously reducing off-target effects outside the tumor to improve safety. In addition to improving delivery technology, combination therapy is also a mainstream trend in overcoming cancer. Whether based on cells or LNP or other carriers, therapeutic cancer vaccines combined with immune checkpoint inhibitors, chemotherapy, radiotherapy, etc., have demonstrated significant advantages in preclinical and clinical studies to exert a synergistic effect and improve therapeutic outcomes.

Nowadays, research is focused on technological breakthroughs in various therapeutic cancer vaccines. By integrating the characteristics of multiple technologies and the continuously accumulated clinical experience, therapeutic cancer vaccine therapy has great potential and application space in the field of cancer treatment. However, future research still requires further improvement and optimization in aspects such as antigen screening, vector design, and production and preparation processes. Strive to reduce production costs, enhance the accuracy of antigens, improve the efficiency and targeting of delivery systems, and verify their long-term efficacy and good safety through large-scale clinical trials.

Therapeutic cancer vaccines, particularly highly personalized neoantigen vaccines, represent a pivotal advancement in cancer immunotherapy. However, they also encounter significant economic and regulatory hurdles that must be overcome to achieve widespread clinical adoption and long-term sustainability. Economically, the primary challenge for personalized vaccines lies in their bespoke nature. The production of autologous vaccines necessitates tumor sequencing for each individual patient, predictive screening of neoantigens, personalized vaccine design, and manufacturing. This intricate process involves compliance with Good Manufacturing Practice (GMP) standards, costly single-lot production, extended cycle times that may delay treatment initiation, and stringent quality-control protocols, all contributing to prohibitively high unit costs (potentially reaching hundreds of thousands or even millions of dollars per patient). Even with allogeneic or shared neoantigen vaccines, which can reduce costs through economies of scale, challenges persist in ensuring universal efficacy across diverse patient populations and minimizing “off-target” toxicity. High development expenses, encompassing complex clinical trials and substantial infrastructure investments, such as decentralized manufacturing facilities or cold-chain logistics networks, ultimately translate into elevated treatment costs, posing critical challenges to patient accessibility and the financial sustainability of healthcare systems.

In the regulatory dimension, the traditional drug approval paradigm is mainly based on uniform, replicable product characteristics and phase III trial data from large-scale homogeneous populations. However, the essence of personalized vaccines—that each patient’s product is different—poses a fundamental challenge to the current regulatory framework. Regulatory agencies (such as the FDA, EMA, etc.) need to address a key issue: defining the “consistent” quality, safety, and efficacy of products based on “individual customization rather than standardization”. This requires an innovative transformation of the regulatory paradigm: developing alternative endpoints based on biomarkers or immune responses to accelerate the approval path; building a new real-world evidence (RWE) collection and evaluation system to accumulate efficacy evidence using individualized data; formulating CMC (Chemistry, Manufacturing, and Controls) guidelines for personalized therapeutic products, emphasizing process control and platform validation rather than the physical consistency of individual products; exploring adaptive licensing pathways, allowing for more flexible initial approval and subsequent data confirmation within a strict framework. Additionally, mechanisms for generating post-market evidence and data sharing after accelerated approval are also crucial.

## 4. Conclusions and Future Directions

Over the past few decades, with the continuous breakthroughs in immunology and precision medicine technologies, people’s understanding of how cancer cells evade immune system monitoring and their roles in the body has been greatly enhanced. This has led to significant progress in tumor immunotherapy, which is constantly developing in a favorable direction for defeating cancer. Previous immune checkpoint inhibitors and cell therapy methods have demonstrated their ability to regress tumors in some studies on hematological malignancies and solid tumors. These advancements have shown the feasibility of applying tumor immunotherapy and therapeutic cancer vaccines. With the continuous development of technology, humans will be able to more accurately identify highly immunogenic neoantigens in the future. Especially the personalized neoantigen vaccines that are currently regarded as having great potential, such vaccines have the advantages of unassailable high specificity and strong immune activation ability. Personalized neoantigen vaccines can avoid attacking normal tissues and significantly reduce off-target toxicity by targeting mutations specific to tumor cells (neoantigens) [271]. In a study, the feasibility, safety, and immunogenicity of a vaccine targeting up to 20 predicted personalized tumor neoantigens were demonstrated, and the vaccine-induced multifunctional CD4^+^ and CD8^+^ T cells targeted 60% (58) and 16% (15) of the 97 unique neoantigens, respectively [271]. This indicates that personalized vaccines have achieved the distinction between mutant antigens and wild-type antigens. Furthermore, neoantigens have not been cleared by the central immune tolerance mechanism, so they are more likely to activate the T-cell response, which is conducive to exerting anti-tumor effects. Personalized neoantigen vaccines are designed based on the gene mutation maps of cancer patients. They activate the CD8^+^ and CD4^+^ T cells of the patient’s own immune system by synthesizing mRNA or DNA to encode tumor-specific neoantigens, thereby achieving precise attacks on tumor cells. Take the mRNA-4157 vaccine as an example. Its fastest-growing personalized mRNA vaccine is delivered to the body through the LNP delivery platform to induce a strong T-cell immune response. In a Phase 2b trial, the combination therapy of mRNA-4157 and Keytruda reduced the risk of recurrence or death in patients with stage III/IV melanoma by 44% and enhanced T-cell-mediated tumor cell destruction [429]. To date, many vaccine platforms targeting personalized neoantigens have entered the clinical trial stage, mainly including vaccines based on peptides, DNA, RNA, adenoviruses, and DC cells. It is worth noting that these vaccines have triggered T-cell immune responses against cancer-related targets, but they face the challenge of low overall immunogenicity. It is believed that with the continuous improvement of various technologies and the development of new ones, combined with targeted and efficient delivery technologies, highly personalized and universal therapeutic vaccines can be developed for different situations. Moreover, through combined treatment, a synergistic effect can be achieved to maximize the therapeutic effect.

## Figures and Tables

**Table 2:** Clinical study of bacterial vector vaccines updated in recent 5 years.

Name	Cancer	ROA	Combination Therapy	NCI Number	Phase	Ref
NECVAX-NEO1	Solid tumors	Orally	Anti-PD-1/PD-L1	NCT06631079	1/2	[140]
NECVAX-NEO1	Triple-negative Breast cancer	Orally	Anti-PD-1 nab-paclitaxel chemotherapy	NCT06631092	1/2	[139]
ADXS11-001	Cervical cancer	i.v.	/	NCT01266460	2	[141]
ADXS11-001	Cervical cancer	i.v.	/	NCT02164461	1	[142]
ADXS11-001	Anal cancer Rectal cancer	i.v.	/	NCT02399813	2	[135]
CRS-207	Pancreatic cancer	i.v.	GVAX vaccine cyclophosphamide	NCT01417000	2	[137]
Saltikva	Pancreatic cancer	Orally	/	NCT04589234	2	[138]
Clostridium Novyi-NT	Solid tumors	i.v.	/	NCT01924689	1	[143]
Clostridium Novyi-NT	Solid tumors	i.v.	Anti-PD-1	NCT03435952	1	[144]
TXSVN	Multiple myeloma	Orally	/	NCT03762291	1	[145]
SGN1	Solid tumors	i.t.	/	NCT05038150	1/2	[146]

**Abbreviation:** Intravenous injection (i.v.); intertumoral injection (i.t.).

**Table 6:** Clinical study of DNA vaccines updated in recent 5 years.

Target	Cancer	ROA	Combination Therapy	NCI Number	Phase	Ref
Emm55 streptococcal antigen	Melanoma	i.t.	/	NCT03655756	1	[361]
TAEK-VAC-HerBy	Chordoma Breast cancer	i.v.	Anti-HER2	NCT04246671	1/2	[363]
pNGVL4aCRTE6E7L2 DNA vaccine	Cervical neoplasia	i.m.	/	NCT04131413	1	[372]
HPV	Cervical cancer Vulvar cancer Vaginal cancer	/	/	NCT02653118	Observational	[373]
HPV	Cervical cancer	/	/	NCT04588402	Observational	[374]
IGFBP-2, HER2, and IGF1R	Breast cancer	i.d.		NCT02780401	1	[364]
Neoantigen DNA vaccine	Prostate cancer	i.m.	Anti-PD-1 or Anti-CTLA4 + PROSTVAC	NCT03532217	1	[368]
Neoantigen DNA vaccine (GNOS-PV02)	Hepatocellular carcinoma	i.d.	Anti-PD-1	NCT04251117	1/2	[360]
Neoantigen DNA vaccine	Recurrent brain tumor	i.m.	/	NCT03988283	1	[375]
pAc/emm55 (pDNA)	Non-melanoma skin cancers	Intralesionally	/	NCT04160065	1	[365]
Prostatic acid phosphatase (pTVG-HP)	Prostate cancer	i.d.	Anti-PD-1	NCT03600350	2	[369]
pTVG-HP DNA vaccine	Prostate cancer	i.d.	Anti-PD-1	NCT04090528	2	[370]
DNA-PEI polyplex	Neuroblastoma	i.m.	/	NCT04049864	1	[376]
Personalized neoantigen DNA vaccine	Glioblastoma	/	/	NCT04015700	1	[366]
Personalized neoantigen DNA vaccine	Glioblastoma	i.m.	/	NCT05743595	1	[367]
TriAd vaccine	Head and neck cancer	i.v.	Anti-PD-L1/TGF-beta Trap (M7824)	NCT04247282	1/2	[377]
GX-188E HPV DNA vaccine	Head and neck cancer	i.m.	Anti-PD-1	NCT05286060	2	[378]
pING-hHER3FL	Advanced cancer	i.m.		NCT03832855	1	[379]
Neoantigen DNA vaccine	Small-cell lung cancer	i.m.	Anti-PD-L1	NCT04397003	2	[380]
CD105/Yb-1/SOX2/CDH3/MDM2-polyepitope plasmid DNA vaccine	Non-small-cell lung cancer	i.d.	/	NCT05242965	2	[381]
CD105/Yb-1/SOX2/CDH3/MDM2-polyepitope plasmid DNA vaccine	Breast cancer	i.v.	/	NCT05455658	2	[362]
Glypican3 (GPC3)-targeted DNA plasmid vaccine (NWRD06)	Hepatocellular carcinoma	i.m.	/	NCT06088459	1	[382]

**Abbreviation:** Intradermal injection (i.d.); intramuscular injection (i.m.); intravenous injection (i.v.); intertumoral injection (i.t.).

## References

[1] Bray F., Laversanne M., Sung H., Ferlay J., Siegel R.L., Soerjomataram I., Jemal A. (2024). Global cancer statistics 2022: GLOBOCAN estimates of incidence and mortality worldwide for 36 cancers in 185 countries. CA Cancer J. Clin..

[2] Yang Y. (2015). Cancer immunotherapy: Harnessing the immune system to battle cancer. J. Clin. Investig..

[3] Pardoll D.M. (2012). The blockade of immune checkpoints in cancer immunotherapy. Nat. Rev. Cancer.

[4] Oliveira G., Wu C.J. (2023). Dynamics and specificities of T cells in cancer immunotherapy. Nat. Rev. Cancer.

[5] Lin M.J., Svensson-Arvelund J., Lubitz G.S., Marabelle A., Melero I., Brown B.D., Brody J.D. (2022). Cancer vaccines: The next immunotherapy frontier. Nat. Cancer.

[6] Igarashi Y., Sasada T. (2020). Cancer Vaccines: Toward the Next Breakthrough in Cancer Immunotherapy. J. Immunol. Res..

[7] Liu N., Xiao X., Zhang Z., Mao C., Wan M., Shen J. (2023). Advances in Cancer Vaccine Research. ACS Biomater. Sci. Eng..

[8] Saxena M., van der Burg S.H., Melief C.J.M., Bhardwaj N. (2021). Therapeutic cancer vaccines. Nat. Rev. Cancer.

[9] Waxman A.G., Zsemlye M.M. (2008). Preventing cervical cancer: The Pap test and the HPV vaccine. Med. Clin. North. Am..

[10] Ahmad M., Asrar R., Ahmed I., Bule M.H. (2024). HPV vaccination: A key strategy for preventing cervical cancer. J. Infect. Public Health.

[11] Seegers S.L., Frasier C., Greene S., Nesmelova I.V., Grdzelishvili V.Z. (2020). Experimental Evolution Generates Novel Oncolytic Vesicular Stomatitis Viruses with Improved Replication in Virus-Resistant Pancreatic Cancer Cells. J. Virol..

[12] Altomonte J., Marozin S., Schmid R.M., Ebert O. (2010). Engineered newcastle disease virus as an improved oncolytic agent against hepatocellular carcinoma. Mol. Ther..

[13] Wollmann G., Rogulin V., Simon I., Rose J.K., van den Pol A.N. (2010). Some attenuated variants of vesicular stomatitis virus show enhanced oncolytic activity against human glioblastoma cells relative to normal brain cells. J. Virol..

[14] Liu T.C., Hwang T., Park B.H., Bell J., Kirn D.H. (2008). The targeted oncolytic poxvirus JX-594 demonstrates antitumoral, antivascular, and anti-HBV activities in patients with hepatocellular carcinoma. Mol. Ther..

[15] Breitbach C.J., Arulanandam R., De Silva N., Thorne S.H., Patt R., Daneshmand M., Moon A., Ilkow C., Burke J., Hwang T.H. (2013). Oncolytic vaccinia virus disrupts tumor-associated vasculature in humans. Cancer Res..

[16] Inoue T., Byrne T., Inoue M., Tait M.E., Wall P., Wang A., Dermyer M.R., Laklai H., Binder J.J., Lees C. (2021). Oncolytic Vaccinia Virus Gene Modification and Cytokine Expression Effects on Tumor Infection, Immune Response, and Killing. Mol. Cancer Ther..

[17] Xu B., Tian L., Chen J., Wang J., Ma R., Dong W., Li A., Zhang J., Antonio Chiocca E., Kaur B. (2021). An oncolytic virus expressing a full-length antibody enhances antitumor innate immune response to glioblastoma. Nat. Commun..

[18] Uche I.K., Kousoulas K.G., Rider P.J.F. (2021). The Effect of Herpes Simplex Virus-Type-1 (HSV-1) Oncolytic Immunotherapy on the Tumor Microenvironment. Viruses.

[19] Boagni D.A., Ravirala D., Zhang S.X. (2021). Current strategies in engaging oncolytic viruses with antitumor immunity. Mol. Ther. Oncolytics.

[20] Yang M., Zhong P., Wei P. (2025). Living Bacteria: A New Vehicle for Vaccine Delivery in Cancer Immunotherapy. Int. J. Mol. Sci..

[21] Pishesha N., Harmand T.J., Ploegh H.L. (2022). A guide to antigen processing and presentation. Nat. Rev. Immunol..

[22] Trinchieri G. (2003). Interleukin-12 and the regulation of innate resistance and adaptive immunity. Nat. Rev. Immunol..

[23] Rossjohn J., Gras S., Miles J.J., Turner S.J., Godfrey D.I., McCluskey J. (2015). T cell antigen receptor recognition of antigen-presenting molecules. Annu. Rev. Immunol..

[24] Gaud G., Lesourne R., Love P.E. (2018). Regulatory mechanisms in T cell receptor signalling. Nat. Rev. Immunol..

[25] Collin M., Bigley V. (2018). Human dendritic cell subsets: An update. Immunology.

[26] Obregon C., Kumar R., Pascual M.A., Vassalli G., Golshayan D. (2017). Update on Dendritic Cell-Induced Immunological and Clinical Tolerance. Front. Immunol..

[27] Eisenbarth S.C. (2019). Dendritic cell subsets in T cell programming: Location dictates function. Nat. Rev. Immunol..

[28] Wculek S.K., Amores-Iniesta J., Conde-Garrosa R., Khouili S.C., Melero I., Sancho D. (2019). Effective cancer immunotherapy by natural mouse conventional type-1 dendritic cells bearing dead tumor antigen. J. Immunother. Cancer.

[29] Zhou Y., Slone N., Chrisikos T.T., Kyrysyuk O., Babcock R.L., Medik Y.B., Li H.S., Kleinerman E.S., Watowich S.S. (2020). Vaccine efficacy against primary and metastatic cancer with in vitro-generated CD103^+^ conventional dendritic cells. J. Immunother. Cancer.

[30] Buteau C., Markovic S.N., Celis E. (2002). Challenges in the development of effective peptide vaccines for cancer. Mayo Clin. Proc..

[31] Kumai T., Lee S., Cho H.I., Sultan H., Kobayashi H., Harabuchi Y., Celis E. (2017). Optimization of Peptide Vaccines to Induce Robust Antitumor CD4 T-cell Responses. Cancer Immunol. Res..

[32] Parmiani G., Castelli C., Dalerba P., Mortarini R., Rivoltini L., Marincola F.M., Anichini A. (2002). Cancer immunotherapy with peptide-based vaccines: What have we achieved? Where are we going?. J. Natl. Cancer Inst..

[33] Harao M., Mittendorf E.A., Radvanyi L.G. (2015). Peptide-based vaccination and induction of CD8+ T-cell responses against tumor antigens in breast cancer. BioDrugs.

[34] Bai H., Lester G.M.S., Petishnok L.C., Dean D.A. (2017). Cytoplasmic transport and nuclear import of plasmid DNA. Biosci. Rep..

[35] Oh S., Kessler J.A. (2018). Design, Assembly, Production, and Transfection of Synthetic Modified mRNA. Methods.

[36] Hager S., Fittler F.J., Wagner E., Bros M. (2020). Nucleic Acid-Based Approaches for Tumor Therapy. Cells.

[37] Wheeler C.J., Black K.L. (2009). DCVax-Brain and DC vaccines in the treatment of GBM. Expert. Opin. Investig. Drugs.

[38] Sosman J.A., Sondak V.K. (2003). Melacine: An allogeneic melanoma tumor cell lysate vaccine. Expert. Rev. Vaccines.

[39] Gulley J.L., Mulders P., Albers P., Banchereau J., Bolla M., Pantel K., Powles T. (2016). Perspectives on sipuleucel-T: Its role in the prostate cancer treatment paradigm. Oncoimmunology.

[40] Saavedra D., Crombet T. (2017). CIMAvax-EGF: A New Therapeutic Vaccine for Advanced Non-Small Cell Lung Cancer Patients. Front. Immunol..

[41] García-Pardo M., Gorria T., Malenica I., Corgnac S., Teixidó C., Mezquita L. (2022). Vaccine Therapy in Non-Small Cell Lung Cancer. Vaccines.

[42] Liu Z., Liu X., Liang J., Liu Y., Hou X., Zhang M., Li Y., Jiang X. (2021). Immunotherapy for Hepatocellular Carcinoma: Current Status and Future Prospects. Front. Immunol..

[43] Bulcha J.T., Wang Y., Ma H., Tai P.W.L., Gao G. (2021). Viral vector platforms within the gene therapy landscape. Signal Transduct. Target. Ther..

[44] Wei D., Xu J., Liu X.Y., Chen Z.N., Bian H. (2018). Fighting Cancer with Viruses: Oncolytic Virus Therapy in China. Hum. Gene Ther..

[45] Xia Z.J., Chang J.H., Zhang L., Jiang W.Q., Guan Z.Z., Liu J.W., Zhang Y., Hu X.H., Wu G.H., Wang H.Q. (2004). [Phase III randomized clinical trial of intratumoral injection of E1B gene-deleted adenovirus (H101) combined with cisplatin-based chemotherapy in treating squamous cell cancer of head and neck or esophagus]. Ai Zheng.

[46] Ledford H. (2015). Cancer-fighting viruses win approval. Nature.

[47] Zeng J., Li X., Sander M., Zhang H., Yan G., Lin Y. (2021). Oncolytic Viro-Immunotherapy: An Emerging Option in the Treatment of Gliomas. Front. Immunol..

[48] Lee A. (2023). Nadofaragene Firadenovec: First Approval. Drugs.

[49] Bai K., Allen C. (2021). How Enhancing Immunity to Low-Risk HPV Could Cure Recurrent Respiratory Papillomatosis. Laryngoscope.

[50] Norberg S.M., Bai K., Sievers C., Robbins Y., Friedman J., Yang X., Kenyon M., Ward E., Schlom J., Gulley J. (2023). The tumor microenvironment state associates with response to HPV therapeutic vaccination in patients with respiratory papillomatosis. Sci. Transl. Med..

[51] Beijing Tsinghua Chang Gung Hospital (2024). Recombinant Human Adenovirus Type 5 Plus HAIC of FOLFOX for Intrahepatic Cholangiocarcinoma. https://clinicaltrials.gov/study/NCT05124002?cond=%20Adenovirus%20%20Cancer&lastUpdPost=2020-01-01_&page=7&rank=66.

[52] Kaufman H.L. (2003). The role of poxviruses in tumor immunotherapy. Surgery.

[53] Holgado M.P., Falivene J., Maeto C., Amigo M., Pascutti M.F., Vecchione M.B., Bruttomesso A., Calamante G., Del Médico-Zajac M.P., Gherardi M.M. (2016). Deletion of A44L, A46R and C12L Vaccinia Virus Genes from the MVA Genome Improved the Vector Immunogenicity by Modifying the Innate Immune Response Generating Enhanced and Optimized Specific T-Cell Responses. Viruses.

[54] Krupa M., Canamero M., Gomez C.E., Najera J.L., Gil J., Esteban M. (2011). Immunization with recombinant DNA and modified vaccinia virus Ankara (MVA) vectors delivering PSCA and STEAP1 antigens inhibits prostate cancer progression. Vaccine.

[55] Breitbach C.J., Burke J., Jonker D., Stephenson J., Haas A.R., Chow L.Q., Nieva J., Hwang T.H., Moon A., Patt R. (2011). Intravenous delivery of a multi-mechanistic cancer-targeted oncolytic poxvirus in humans. Nature.

[56] Park B.H., Hwang T., Liu T.C., Sze D.Y., Kim J.S., Kwon H.C., Oh S.Y., Han S.Y., Yoon J.H., Hong S.H. (2008). Use of a targeted oncolytic poxvirus, JX-594, in patients with refractory primary or metastatic liver cancer: A phase I trial. Lancet Oncol..

[57] Heo J., Reid T., Ruo L., Breitbach C.J., Rose S., Bloomston M., Cho M., Lim H.Y., Chung H.C., Kim C.W. (2013). Randomized dose-finding clinical trial of oncolytic immunotherapeutic vaccinia JX-594 in liver cancer. Nat. Med..

[58] Abou-Alfa G.K., Galle P.R., Chao Y., Erinjeri J., Heo J., Borad M.J., Luca A., Burke J., Pelusio A., Agathon D. (2024). PHOCUS: A Phase 3, Randomized, Open-Label Study of Sequential Treatment with Pexa-Vec (JX-594) and Sorafenib in Patients with Advanced Hepatocellular Carcinoma. Liver Cancer.

[59] Theravectys S.A. (2024). A Study to Evaluate Lenti-HPV-07 Immunotherapy Against HPV+ Cervical or Oropharyngeal Cancer. https://clinicaltrials.gov/study/NCT06319963?cond=NCT06319963&rank=1.

[60] University Medical Center Groningen (2023). Vvax001 Cancer Vaccine in Premalignant Cervical Lesions—Phase II (Vvax). https://clinicaltrials.gov/study/NCT06015854?cond=NCT06015854&rank=1.

[61] Gregory K., Friedman M., University of Alabama at Birmingham (Responsible Party) (2024). HSV G207 Alone or With a Single Radiation Dose in Children With Progressive or Recurrent Supratentorial Brain Tumors. https://clinicaltrials.gov/study/NCT02457845?cond=NCT02457845&rank=1.

[62] D’Alise A.M., Brasu N., De Intinis C., Leoni G., Russo V., Langone F., Baev D., Micarelli E., Petiti L., Picelli S. (2022). Adenoviral-based vaccine promotes neoantigen-specific CD8^+^ T cell stemness and tumor rejection. Sci. Transl. Med..

[63] Targovax ASA (Targovax Oy) (2021). A Pilot Study of Sequential ONCOS-102, an Engineered Oncolytic Adenovirus Expressing GMCSF, and Pembrolizumab in Patients With Advanced or Unresectable Melanoma Progressing After Programmed Cell Death Protein 1 (PD1) Blockade. https://clinicaltrials.gov/study/NCT03003676?cond=%20Adenovirus%20%20Cancer&lastUpdPost=2020-01-01_&page=8&rank=74.

[64] Hospital F.C. (2023). Recombinant Human Adenovirus Type 5 Injection Combined With PD-1 Monoclonal Antibody and Nab-paclitaxel in the Treatment of Patients With Liver Metastases From Malignant Melanoma. https://clinicaltrials.gov/study/NCT05664139?cond=%20Adenovirus%20%20Cancer&lastUpdPost=2020-01-01_&page=7&rank=69.

[65] TILT Biotherapeutics Ltd. (2024). Oncolytic Adenovirus TILT-123 and Avelumab for Treatment of Solid Tumors Refractory to or Progressing After Anti-PD(L)1 (AVENTIL). https://clinicaltrials.gov/study/NCT05222932?cond=%20Adenovirus%20%20Cancer&lastUpdPost=2020-01-01_&page=5&rank=48.

[66] Tim Greten M.D., National Cancer Institute (NCI) (2023). VB-111 in Combination With Nivolumab in People With Metastatic Colorectal Cancer (mCRC). https://clinicaltrials.gov/study/NCT04166383?cond=%20Adenovirus%20%20Cancer&lastUpdPost=2020-01-01_&page=9&rank=85.

[67] Liu F., China Medical University (2024). A Clinical Study of BioTTT001 in Combination With Toripalimab and Regorafenib in Patients With Colorectal Cancer. https://clinicaltrials.gov/study/NCT06283134?cond=%20Adenovirus%20%20Cancer&lastUpdPost=2020-01-01_&page=6&rank=58.

[68] DNAtrix, Inc. (2021). Combination Adenovirus + Pembrolizumab to Trigger Immune Virus Effects (CAPTIVE). https://clinicaltrials.gov/study/NCT02798406?cond=%20Adenovirus%20%20Cancer&lastUpdPost=2020-01-01_&rank=4.

[69] Therapeutics A. (2021). A Study of Ad-RTS-hIL-12 With Veledimex in Subjects With Glioblastoma or Malignant Glioma. https://clinicaltrials.gov/study/NCT02026271?cond=%20Adenovirus%20%20Cancer&lastUpdPost=2020-01-01_&page=10&rank=92.

[70] TILT Biotherapeutics Ltd. (2024). Oncolytic Adenovirus TILT-123 With Pembrolizumab as Treatment for Refractory Non-Small Cell Lung Cancer. https://clinicaltrials.gov/study/NCT06125197?cond=%20Adenovirus%20%20Cancer&lastUpdPost=2020-01-01_&page=3&rank=21.

[71] Wu H., Henan Cancer Hospital (2024). Local Injection and Systemic Therapy in the Treatment of NSCLC. https://clinicaltrials.gov/study/NCT06618391?cond=%20Adenovirus%20%20Cancer&lastUpdPost=2020-01-01_&page=8&rank=72.

[72] Turnstone Biologics, Corp (2020). Oncolytic MG1-MAGEA3 With Ad-MAGEA3 Vaccine in Combination With Pembrolizumab for Non-Small Cell Lung Cancer Patients. https://clinicaltrials.gov/study/NCT02879760?cond=%20Adenovirus%20%20Cancer&lastUpdPost=2020-01-01_&page=8&rank=80.

[73] Kwon D., Henry Ford Health System (2022). Phase 1 Trial of Interleukin 12 Gene Therapy for Metastatic Pancreatic Cancer. https://clinicaltrials.gov/study/NCT03281382?cond=NCT03281382&rank=1.

[74] Seoul National University Hospital (2019). Clinical Trial Phase I for Theragene in Combination With Chemotherapy for the Locally Advanced Pancreatic Cancer (Theragene). https://clinicaltrials.gov/study/NCT02894944?cond=NCT02894944&rank=1.

[75] Lokon Pharma AB (2025). LOAd703 Oncolytic Virus Therapy for Pancreatic Cancer. https://clinicaltrials.gov/study/NCT02705196?cond=%20Adenovirus%20%20Cancer&lastUpdPost=2020-01-01_&page=3&rank=27.

[76] Henry Ford Health System (2024). Adenovirus Mediated Suicide Gene Therapy With Radiotherapy in Progressive Astrocytoma. https://clinicaltrials.gov/study/NCT05686798?cond=%20Adenovirus%20%20Cancer&lastUpdPost=2020-01-01_&page=3&rank=23.

[77] Binhai Hospital of Fujian Medical University (2023). Safety and Tolerability Study of Recombinant L-IFN Adenovirus Injection in Patients With Recurrent Glioblastoma (YSCH-01). https://clinicaltrials.gov/study/NCT05914935?cond=%20Adenovirus%20%20Cancer&lastUpdPost=2020-01-01_&page=7&rank=64.

[78] M.D. Anderson Cancer Center (2025). MSC-DNX-2401 in Treating Patients With Recurrent High-Grade Glioma. https://clinicaltrials.gov/study/NCT03896568?cond=%20Adenovirus%20%20Cancer&lastUpdPost=2020-01-01_&page=9&rank=86.

[79] Uppsala University (2024). Study of Recombinant Adenovirus AdVince in Patients With Neuroendocrine Tumors; Safety and Efficacy (RADNET). https://clinicaltrials.gov/study/NCT02749331?cond=%20Adenovirus%20%20Cancer&lastUpdPost=2020-01-01_&rank=5.

[80] Akamis Bio (2024). First in Human Study With NG-641, a Tumour Selective Transgene Expressing Adenoviral Vector (STAR). https://clinicaltrials.gov/study/NCT04053283?cond=%20Adenovirus%20%20Cancer&lastUpdPost=2020-01-01_&page=2&rank=12.

[81] Akamis Bio (2022). First in Human Study of NG-350A (an Oncolytic Adenoviral Vector Which Expresses an Anti-CD40 Antibody) (FORTITUDE). https://clinicaltrials.gov/study/NCT03852511?cond=%20Adenovirus%20%20Cancer&lastUpdPost=2020-01-01_&page=2&rank=13.

[82] Siddiqui F., Henry Ford Health System (2024). Phase 1 Trial of Interleukin 12 Gene Therapy for Locally Recurrent Prostate Cancer. https://clinicaltrials.gov/study/NCT02555397?cond=NCT02555397&rank=1.

[83] Momotaro-Gene Inc. (2020). Use of Recombinant Adenovirus Therapy to Treat Localized Prostate Cancer. https://clinicaltrials.gov/study/NCT01931046?cond=%20Adenovirus%20%20Cancer&lastUpdPost=2020-01-01_&page=3&rank=22.

[84] Lubaroff D.M., University of Iowa (2023). Phase II Study of Adenovirus/PSA Vaccine in Men With Hormone—Refractory Prostate Cancer (APP22). https://clinicaltrials.gov/study/NCT00583024?cond=%20Adenovirus%20%20Cancer&lastUpdPost=2020-01-01_&page=4&rank=36.

[85] Orca Therapeutics B.V. (2023). First in Man Clinical Study to Evaluate Safety and Tolerability of an Oncolytic Adenovirus in Prostate Cancer Patients. https://clinicaltrials.gov/study/NCT04097002?cond=%20Adenovirus%20%20Cancer&lastUpdPost=2020-01-01_&page=8&rank=73.

[86] Lubaroff D.M., University of Iowa (2023). Phase II Study of Adenovirus/PSA Vaccine in Men With Recurrent Prostate Cancer After Local Therapy APP21 (APP21). https://clinicaltrials.gov/study/NCT00583752?cond=%20Adenovirus%20%20Cancer&lastUpdPost=2020-01-01_&page=4&rank=37.

[87] University of Birmingham (2021). A Clinical Trial of AdNRGM Plus CB1954 in Prostate Cancer (AdUP). https://clinicaltrials.gov/study/NCT04374240?cond=%20Adenovirus%20%20Cancer&lastUpdPost=2020-01-01_&page=5&rank=45.

[88] Gao Q., Tongji Hospital (2024). The Safety, Tolerability, and Efficacy of KD01 in Cervical Malignancies. https://clinicaltrials.gov/study/NCT06552598?cond=%20Adenovirus%20%20Cancer&lastUpdPost=2020-01-01_&page=3&rank=25.

[89] Fujian Cancer Hospital (2024). Efficacy and Safety of AK104 Combined With Chemotherapy and Recombinant Human Adenovirus 5 Injection in Cervical Cancer. https://clinicaltrials.gov/study/NCT06455046?cond=%20Adenovirus%20%20Cancer&lastUpdPost=2020-01-01_&page=5&rank=41.

[90] University of Pennsylvania (2020). Gene Therapy for Pleural Malignancies. https://clinicaltrials.gov/study/NCT00299962?cond=%20Adenovirus%20%20Cancer&lastUpdPost=2020-01-01_&page=4&rank=31.

[91] Abramson Cancer Center at Penn Medicine (2020). Intrapleural BG00001 in Treating Patients With Malignant Pleural Mesothelioma or Malignant Pleural Effusions. https://clinicaltrials.gov/study/NCT00066404?cond=%20Adenovirus%20%20Cancer&lastUpdPost=2020-01-01_&page=4&rank=38.

[92] Eastern Cooperative Oncology Group (2023). Gene Therapy in Treating Patients With Non-small Cell Lung Cancer That Cannot Be Surgically Removed. https://clinicaltrials.gov/study/NCT00003649?cond=%20Adenovirus%20%20Cancer&lastUpdPost=2020-01-01_&page=4&rank=40.

[93] Strauss J., National Cancer Institute (NCI) (2020). Multi-Targeted Recombinant Ad5 (CEA/MUC1/Brachyury) Based Immunotherapy Vaccine Regimen in People With Advanced Cancer. https://clinicaltrials.gov/study/NCT03384316?cond=%20Adenovirus%20%20Cancer&lastUpdPost=2020-01-01_&page=6&rank=55.

[94] Akamis Bio (2020). Mechanism of Action Trial of ColoAd1 (MOA). https://clinicaltrials.gov/study/NCT02053220?cond=%20Adenovirus%20%20Cancer&lastUpdPost=2020-01-01_&page=6&rank=56.

[95] Hodi F.S., Dana-Farber Cancer Institute (2021). Vaccine Trial for Clear Cell Sarcoma, Pediatric Renal Cell Carcinoma, Alveolar Soft Part Sarcoma and Children With Stage IV Melanoma. https://clinicaltrials.gov/study/NCT00258687?cond=GVAX&page=4&rank=33.

[96] GeoVax, Inc. (2024). Safety and Efficacy of Repeat Administration of Ad/PNP and Fludarabine Phosphate in Patients With Local Head/Neck Cancer. https://clinicaltrials.gov/study/NCT03754933?cond=%20Adenovirus%20%20Cancer&lastUpdPost=2020-01-01_&page=10&rank=93.

[97] University of Oxford (2025). Chemoradiation With Enadenotucirev as a Radiosensitiser in Locally Advanced Rectal Cancer (CEDAR). https://clinicaltrials.gov/study/NCT03916510?cond=%20Adenovirus%20%20Cancer&lastUpdPost=2020-01-01_&page=11&rank=101.

[98] Ferring Ventures Limited (2025). Efficacy & Safety of RAd-IFN Administered with Celecoxib & Gemcitabine in Patients with Malignant Pleural Mesothelioma (INFINITE). https://clinicaltrials.gov/study/NCT03710876?cond=%20Adenovirus%20%20Cancer&lastUpdPost=2020-01-01_&page=11&rank=103.

[99] Abramson Cancer Center at Penn Medicine (2020). Combination Gene Transfer and Chemotherapy. https://clinicaltrials.gov/study/NCT01119664?cond=%20Adenovirus%20%20Cancer&lastUpdPost=2020-01-01_&page=11&rank=102.

[100] Tianjin Medical University Cancer Institute and Hospital (2023). H101 Combined With TACE for Primary Hepatocellular Carcinoma With Portal Vein Thrombosis. https://clinicaltrials.gov/study/NCT05872841?cond=%20Adenovirus%20%20Cancer&lastUpdPost=2020-01-01_&page=10&rank=96.

[101] The First Affiliated Hospital of Bengbu Medical University (2024). Efficacy and Safety of Intratumoral Injection of Recombinant Human Adenovirus Type 5 Combined With Tislelizumab and Lenvatinib in the Treatment of Advanced Hepatocellular Carcinoma. https://clinicaltrials.gov/study/NCT06253598?cond=%20Adenovirus%20%20Cancer&lastUpdPost=2020-01-01_&page=10&rank=95.

[102] Henan Cancer Hospital (2024). Sequential T and I With H101 Via HAI for BCLC C Stage HCC: A Prospective Single-Center Single-Arm Pilot Study. https://clinicaltrials.gov/study/NCT06685354?cond=%20Adenovirus%20%20Cancer&lastUpdPost=2020-01-01_&page=10&rank=97.

[103] First Affiliated Hospital Xi’an Jiaotong University (2022). Effect and Safety of Recombinant Human Adenovirus Type 5 in Advanced HCC With Stable Disease After Sorafenib Treatment. https://clinicaltrials.gov/study/NCT05113290?cond=%20Adenovirus%20%20Cancer&lastUpdPost=2020-01-01_&page=9&rank=82.

[104] Ming S., Sun Yat-sen University (2020). HAIC Plus H101 vs HAIC Alone for Unresectable HCC at BCLC A-B. https://clinicaltrials.gov/study/NCT03780049?cond=%20Adenovirus%20%20Cancer&lastUpdPost=2020-01-01_&page=10&rank=91.

[105] Beijing Syngentech Co., Ltd. (2023). SynOV1.1 Intratumoral Injection Study. https://clinicaltrials.gov/study/NCT04612504?cond=%20Adenovirus%20%20Cancer&lastUpdPost=2020-01-01_&page=9&rank=84.

[106] Liu F., China Medical University (2024). A Clinical Study of BioTTT001 in Combination With SOX and Toripalimab in Patients With Gastric Cancer. https://clinicaltrials.gov/study/NCT06283121?cond=%20Adenovirus%20%20Cancer&lastUpdPost=2020-01-01_&page=6&rank=57.

[107] Fundació Sant Joan de Déu (2024). Evaluate Safety and the Oncolitic Adenovirus VCN-01 Activity in Patients With Refractory Retinoblastoma (RTB). https://clinicaltrials.gov/study/NCT03284268?cond=%20Adenovirus%20%20Cancer&lastUpdPost=2020-01-01_&page=7&rank=65.

[108] TILT Biotherapeutics Ltd. (2024). Oncolytic Adenovirus Coding for TNFa and IL2 (TILT-123) With Pembrolizumab or Pembrolizumab and Pegylated Liposomal Doxorubicin as Treatment for Ovarian Cancer. https://clinicaltrials.gov/study/NCT05271318?cond=%20Adenovirus%20%20Cancer&lastUpdPost=2020-01-01_&page=8&rank=71.

[109] University of Texas Southwestern Medical Center (2021). Gene Therapy in Treating Patients With Advanced Recurrent or Persistent Ovarian Cancer or Primary Peritoneal Cancer. https://clinicaltrials.gov/study/NCT00003450?cond=%20Adenovirus%20%20Cancer&lastUpdPost=2020-01-01_&page=5&rank=46.

[110] TILT Biotherapeutics Ltd. (2024). TNFalpha and Interleukin 2 Coding Oncolytic Adenovirus TILT-123 During TIL Treatment of Advanced Melanoma (TUNINTIL). https://clinicaltrials.gov/study/NCT04217473?cond=%20Adenovirus%20%20Cancer&lastUpdPost=2020-01-01_&page=4&rank=39.

[111] Akamis Bio (2024). Study of NG-641 in Combination With Nivolumab in Metastatic or Advanced Epithelial Tumours (NEBULA). https://clinicaltrials.gov/study/NCT05043714?cond=%20Adenovirus%20%20Cancer&lastUpdPost=2020-01-01_&page=3&rank=26.

[112] Akamis Bio (2024). Study of NG-350A Plus Pembrolizumab in Metastatic or Advanced Epithelial Tumours (FORTIFY) (FORTIFY). https://clinicaltrials.gov/study/NCT05165433?cond=%20Adenovirus%20%20Cancer&lastUpdPost=2020-01-01_&page=2&rank=19.

[113] Akamis Bio (2024). NG-350A Plus Chemoradiotherapy for Locally Advanced Rectal Cancer (FORTRESS). https://clinicaltrials.gov/study/NCT06459869?cond=%20Adenovirus%20%20Cancer&lastUpdPost=2020-01-01_&page=5&rank=43.

[114] Lokon Pharma AB (2024). Trial Investigating an Immunostimulatory Oncolytic Adenovirus for Cancer. https://clinicaltrials.gov/study/NCT03225989?cond=%20Adenovirus%20%20Cancer&lastUpdPost=2020-01-01_&page=2&rank=15.

[115] Seoul National University Bundang Hospital (2021). An Exploratory Trial to Evaluate Efficacy and Safety for Combination Treatment of Adenovirus Double Suicide Gene Therapy. https://clinicaltrials.gov/study/NCT04739046?cond=%20Adenovirus%20%20Cancer&lastUpdPost=2020-01-01_&page=3&rank=28.

[116] MultiVir, Inc. (2020). Safety and Efficacy of p53 Gene Therapy Combined With Immune Checkpoint Inhibitors in Solid Tumors. https://clinicaltrials.gov/study/NCT03544723?cond=%20Adenovirus%20%20Cancer&lastUpdPost=2020-01-01_&page=5&rank=44.

[117] Makawita S., Baylor College of Medicine (2025). Binary Oncolytic Adenovirus in Combination With HER2-Specific Autologous CAR VST, Advanced HER2 Positive Solid Tumors (VISTA). https://clinicaltrials.gov/study/NCT03740256?cond=%20Adenovirus%20%20Cancer&lastUpdPost=2020-01-01_&page=4&rank=32.

[118] Shanghai Fengxian District Central Hospital (2022). Safety and Efficacy of Recombinant Oncolytic Adenovirus L-IFN Injection in Relapsed/Refractory Solid Tumors Clinical Study (YSCH-01). https://clinicaltrials.gov/study/NCT05180851?cond=%20Adenovirus%20%20Cancer&lastUpdPost=2020-01-01_&page=3&rank=24.

[119] TILT Biotherapeutics Ltd. (2024). TNFα and IL-2 Coding Oncolytic Adenovirus TILT-123 Monotherapy (TUNIMO). https://clinicaltrials.gov/study/NCT04695327?cond=%20Adenovirus%20%20Cancer&lastUpdPost=2020-01-01_&rank=2.

[120] EpicentRx, Inc. (2024). A Study of AdAPT-001 in Subjects With Sarcoma and Refractory Solid Tumors (BETA-PRIME). https://clinicaltrials.gov/study/NCT04673942?cond=%20Adenovirus%20%20Cancer&lastUpdPost=2020-01-01_&page=8&rank=77.

[121] Bavarian Nordic (2019). A Randomized, Double-blind, Phase 3 Efficacy Trial of PROSTVAC-V/F +/- GM-CSF in Men With Asymptomatic or Minimally Symptomatic Metastatic Castrate-Resistant Prostate Cancer (Prospect). https://clinicaltrials.gov/study/NCT01322490?cond=PROSTVAC&rank=4.

[122] Gulley J.L., Borre M., Vogelzang N.J., Ng S., Agarwal N., Parker C.C., Pook D.W., Rathenborg P., Flaig T.W., Carles J. (2019). Phase III Trial of PROSTVAC in Asymptomatic or Minimally Symptomatic Metastatic Castration-Resistant Prostate Cancer. J. Clin. Oncol..

[123] Gulley J., National Cancer Institute (NCI) (Responsible Party) (2024). PROSTVAC in Combination With Nivolumab in Men With Prostate Cancer. https://clinicaltrials.gov/study/NCT02933255?cond=NCT02933255&rank=1.

[124] Transgene (2024). A Trial Evaluating TG4050 in Ovarian Carcinoma. https://clinicaltrials.gov/study/NCT03839524?cond=TG4050&rank=2.

[125] Transgene (2025). A Clinical Trial Evaluating TG4050 in Head and Neck Cancer. https://clinicaltrials.gov/study/NCT04183166?cond=TG4050&rank=1.

[126] Nouscom SRL (2024). Nous-209 Genetic Vaccine for the Treatment of Microsatellite Unstable Solid Tumors. https://clinicaltrials.gov/study/NCT04041310?cond=NCT04041310&rank=1.

[127] D’Alise A.M., Leoni G., Cotugno G., Siani L., Vitale R., Ruzza V., Garzia I., Antonucci L., Micarelli E., Venafra V. (2024). Phase I Trial of Viral Vector-Based Personalized Vaccination Elicits Robust Neoantigen-Specific Antitumor T-Cell Responses. Clin. Cancer Res..

[128] Nejman D., Livyatan I., Fuks G., Gavert N., Zwang Y., Geller L.T., Rotter-Maskowitz A., Weiser R., Mallel G., Gigi E. (2020). The human tumor microbiome is composed of tumor type-specific intracellular bacteria. Science.

[129] Pawelek J.M., Low K.B., Bermudes D. (1997). Tumor-targeted Salmonella as a novel anticancer vector. Cancer Res..

[130] Naghavian R., Faigle W., Oldrati P., Wang J., Toussaint N.C., Qiu Y., Medici G., Wacker M., Freudenmann L.K., Bonté P.E. (2023). Microbial peptides activate tumour-infiltrating lymphocytes in glioblastoma. Nature.

[131] Kalaora S., Nagler A., Nejman D., Alon M., Barbolin C., Barnea E., Ketelaars S.L.C., Cheng K., Vervier K., Shental N. (2021). Identification of bacteria-derived HLA-bound peptides in melanoma. Nature.

[132] Redenti A., Im J., Redenti B., Li F., Rouanne M., Sheng Z., Sun W., Gurbatri C.R., Huang S., Komaranchath M. (2024). Probiotic neoantigen delivery vectors for precision cancer immunotherapy. Nature.

[133] Chen G., Wei D.P., Jia L.J., Tang B., Shu L., Zhang K., Xu Y., Gao J., Huang X.F., Jiang W.H. (2009). Oral delivery of tumor-targeting Salmonella exhibits promising therapeutic efficacy and low toxicity. Cancer Sci..

[134] Basu P., Mehta A., Jain M., Gupta S., Nagarkar R.V., John S., Petit R. (2018). A Randomized Phase 2 Study of ADXS11-001 Listeria monocytogenes-Listeriolysin O Immunotherapy With or Without Cisplatin in Treatment of Advanced Cervical Cancer. Int. J. Gynecol. Cancer.

[135] Advaxis, Inc. (2023). A Phase 2 Study of Axalimogene Filolisbac (ADXS11-001) in Participants With Carcinoma of the Anorectal Canal. https://clinicaltrials.gov/study/NCT02399813?cond=ADXS11-001&rank=4.

[136] Advaxis, Inc. (2023). Study of ADXS11-001 in Participants With High Risk Locally Advanced Cervical Cancer (AIM2CERV). https://clinicaltrials.gov/study/NCT02853604?cond=ADXS11-001&rank=2.

[137] Aduro Biotech, Inc. (2018). Safety and Efficacy of Combination Listeria/GVAX Immunotherapy in Pancreatic Cancer. https://clinicaltrials.gov/study/NCT01417000?cond=GVAX&rank=4.

[138] Salspera LLC Saltikva for Metastatic Pancreatic Cancer. https://clinicaltrials.gov/study/NCT04589234?cond=NCT04589234&rank=1.

[139] NEC Bio B.V Personalised Neoantigen-targeting Cancer Vaccine NECVAX-NEO1 in Neoadjuvant Triple-negative Breast Cancer. https://clinicaltrials.gov/study/NCT06631092?cond=NCT06631092&rank=1.

[140] NEC Bio B.V An Open-label, Phase I/II Multicenter Clinical Trial of NECVAX-NEO1 in Addition to Anti-PD-1 or Anti-PD-L1 Monoclonal Antibody Therapy in Patients With Solid Tumors. https://clinicaltrials.gov/study/NCT06631079?cond=NCT06631079&rank=1.

[141] Gynecologic Oncology Group (2020). Vaccine Therapy in Treating Patients With Persistent or Recurrent Cervical Cancer. https://clinicaltrials.gov/study/NCT01266460?cond=ADXS11-001&rank=7.

[142] Advaxis, Inc. (2024). Axalimogene Filolisbac (ADXS11-001) High Dose in Women With Human Papillomavirus (HPV) + Cervical Cancer. https://clinicaltrials.gov/study/NCT02164461?cond=ADXS11-001&rank=6.

[143] BioMed Valley Discoveries, Inc. Safety Study of Intratumoral Injection of Clostridium Novyi-NT Spores to Treat Patients With Solid Tumors That Have Not Responded to Standard Therapies. https://clinicaltrials.gov/study/NCT01924689?cond=NCT01924689&rank=1.

[144] M.D. Anderson Cancer Center Pembrolizumab with Intratumoral Injection of Clostridium Novyi-NT. https://clinicaltrials.gov/study/NCT03435952?cond=NCT03435952&rank=1.

[145] Lulla P., Baylor College of Medicine Multiple Myeloma Trial of Orally Administered Salmonella Based Survivin Vaccine (MAPSS). https://clinicaltrials.gov/study/NCT03762291?cond=NCT03762291&rank=1.

[146] Guangzhou Sinogen Pharmaceutical Co. Ltd. Study of SGN1 in Patients With Advanced Solid Tumor. https://clinicaltrials.gov/study/NCT05038150?cond=NCT05038150&rank=1.

[147] Garg A.D., Coulie P.G., Van den Eynde B.J., Agostinis P. (2017). Integrating Next-Generation Dendritic Cell Vaccines into the Current Cancer Immunotherapy Landscape. Trends Immunol..

[148] Sabado R.L., Balan S., Bhardwaj N. (2017). Dendritic cell-based immunotherapy. Cell Res..

[149] Dudek A.M., Martin S., Garg A.D., Agostinis P. (2013). Immature, Semi-Mature, and Fully Mature Dendritic Cells: Toward a DC-Cancer Cells Interface That Augments Anticancer Immunity. Front. Immunol..

[150] Mempel T.R., Henrickson S.E., Von Andrian U.H. (2004). T-cell priming by dendritic cells in lymph nodes occurs in three distinct phases. Nature.

[151] Fan T., Zhang M., Yang J., Zhu Z., Cao W., Dong C. (2023). Therapeutic cancer vaccines: Advancements, challenges, and prospects. Signal Transduct. Target. Ther..

[152] Palucka K., Banchereau J. (2013). Dendritic-cell-based therapeutic cancer vaccines. Immunity.

[153] Northwest Biotherapeutics (2022). Study of a Drug [DCVax®-L] to Treat Newly Diagnosed GBM Brain Cancer (GBM). https://clinicaltrials.gov/study/NCT00045968?cond=NCT00045968&rank=1.

[154] Affiliated Hospital to Academy of Military Medical Sciences (2016). DC Vaccine Combined With CIK Cells in Patients With SCLC. https://clinicaltrials.gov/study/NCT02688673?cond=DC%20vaccine&page=3&rank=23.

[155] Bouchaab H., Centre Hospitalier Universitaire Vaudois (2023). Personalized DC Vaccines in Non Small Cell Lung Cancer. https://clinicaltrials.gov/study/NCT05195619?cond=DC%20vaccine&page=4&rank=39.

[156] H. Lee Moffitt Cancer Center and Research Institute (2024). HER-2 Pulsed DC Vaccine to Prevent Recurrence of Invasive Breast Cancer (Adjuvant). https://clinicaltrials.gov/study/NCT02063724?cond=DC%20vaccine&page=3&rank=22.

[157] H. Lee Moffitt Cancer Center and Research Institute (2024). HER-2 Pulsed DC Vaccine to Prevent Recurrence of Invasive Breast Cancer Post Neoadjuvant Chemotherapy (Neoadjuvant). https://clinicaltrials.gov/study/NCT02061423?cond=DC%20vaccine&page=3&rank=30.

[158] H. Lee Moffitt Cancer Center and Research Institute (2024). Personalized Dendritic Cell Vaccine Pilot for High Risk TNBC After Neoadjuvant Therapy. https://clinicaltrials.gov/study/NCT06435351?cond=DC%20vaccine&page=5&rank=47.

[159] Lopez C.A.P., Universidad Nacional de Colombia (2021). Personalized Vaccine for Cancer Immunotherapy. https://www.clinicaltrials.gov/study/NCT04879888?cond=NCT04879888&rank=1.

[160] Universidad Nacional de Colombia (2022). Breast Cancer Neoantigen Vaccination With Autologous Dendritic Cells. https://www.clinicaltrials.gov/study/NCT04105582?cond=NCT04105582&rank=1.

[161] Butterfield L.H., University of Pittsburgh (2017). Multiple Antigen-Engineered DC Vaccine for Melanoma. https://clinicaltrials.gov/study/NCT01622933?cond=DC%20vaccine&page=2&rank=14.

[162] Elios Therapeutics, LLC. (2022). Phase IIB TL + YCWP + DC in Melanoma. https://clinicaltrials.gov/study/NCT02301611?cond=DC%20vaccine&page=2&rank=19.

[163] Fuente M.D.L., University of Miami (2022). Dendritic Cell (DC) Vaccine for Malignant Glioma and Glioblastoma. https://clinicaltrials.gov/study/NCT01808820?cond=DC%20vaccine&page=3&rank=21.

[164] Elios Therapeutics, LLC (2024). Multi-center Phase I/IIa Trial of an Autologous Tumor Lysate (TL) + Yeast Cell Wall Particles (YCWP) + Dendritic Cells (DC) Vaccine in Addition to Standard of Care Checkpoint Inhibitor of Choice in Metastatic Melanoma Patients With Measurable Disease. https://clinicaltrials.gov/study/NCT02678741?cond=DC%20vaccine&page=8&rank=75.

[165] Storkus W.J., University of Pittsburgh (2019). Dendritic Cell Vaccines + Dasatinib for Metastatic Melanoma. https://clinicaltrials.gov/study/NCT01876212?cond=cancer%20%20cell%20vaccine&start=2014-01-01_2024-12-31&term=Cancer%20Vaccine&intr=cancer%20cell&page=5&rank=43.

[166] Radboud University Medical Center (2021). MiHA-loaded PD-L-silenced DC Vaccination After Allogeneic SCT (PSCT19). https://clinicaltrials.gov/study/NCT02528682?cond=NCT02528682&rank=1..

[167] Sarivalasis A., Centre Hospitalier Universitaire Vaudois (2023). PEP-DC and OC-DC Vaccine in High Grade Serous Ovarian Carcinoma (CHUV-OVACURE). https://clinicaltrials.gov/study/NCT05714306?cond=DC%20vaccine&rank=2.

[168] Ding Z., Sichuan University (2018). Personalized DC Vaccine for Lung Cancer (SKLB1608). https://clinicaltrials.gov/study/NCT02956551?cond=DC%20vaccine&rank=3.

[169] Ding Z., Sichuan University (2024). Personalized DC Vaccine for Postoperative Cancer. https://clinicaltrials.gov/study/NCT04147078?cond=DC%20vaccine&page=2&rank=11.

[170] Shenzhen People’s Hospital (2019). Neoantigen-primed DC Vaccines Therapy for Refractory Lung Cancer. https://clinicaltrials.gov/study/NCT03871205?cond=DC%20vaccine&page=5&rank=46.

[171] Cancer Research UK (2024). AST-VAC2 Vaccine in Patients With Non-small Cell Lung Cancer. https://clinicaltrials.gov/study/NCT03371485?cond=NCT03371485&rank=1.

[172] Shenzhen Geno-Immune Medical Institute (2020). Immune Modulatory DC Vaccine Against Brain Tumor. https://clinicaltrials.gov/study/NCT03914768?cond=DC%20vaccine&rank=4.

[173] Ji N., Beijing Tiantan Hospital (2016). Safety and Efficacy of IDH1R132H-DC Vaccine in Gliomas. https://clinicaltrials.gov/study/NCT02771301?cond=DC%20vaccine&rank=7.

[174] Yao Y., Huashan Hospital (2021). Study Details | Neoadjuvant PD-1 Antibody Alone or Combined With DC Vaccines for Recurrent Glioblastoma. https://clinicaltrials.gov/study/NCT04888611?cond=DC%20vaccine&rank=9.

[175] Archer G., Duke University (2020). Nivolumab With DC Vaccines for Recurrent Brain Tumors (AVERT). https://clinicaltrials.gov/study/NCT02529072?cond=DC%20vaccine&rank=10.

[176] Khasraw M., Duke University (2023). DC Migration Study for Newly-Diagnosed GBM (ELEVATE). https://clinicaltrials.gov/study/NCT02366728?cond=DC%20vaccine&page=5&rank=44.

[177] The Second Hospital of Shandong University (2020). Safety and Efficacy Study for MG-7-DC Vaccine in Gastric Cancer Treatment. https://clinicaltrials.gov/study/NCT04567069?cond=DC%20vaccine&rank=6.

[178] Qiu M., West China Hospital (2024). Tumor Antigen-sensitized DC Vaccine for Colorectal Cancer Liver Metastases. https://www.clinicaltrials.gov/study/NCT06545630?cond=NCT06545630&rank=1.

[179] University of Pennsylvania (2024). DC Vaccine in Colorectal Cancer. https://clinicaltrials.gov/study/NCT03730948?cond=DC%20vaccine&page=3&rank=27.

[180] Radboud University Medical Center (2024). Dendritic Cell Vaccination in Patients With Lynch Syndrome or Colorectal Cancer With MSI. https://www.clinicaltrials.gov/study/NCT01885702?cond=NCT01885702&rank=1.

[181] Carreno B.M., Magrini V., Becker-Hapak M., Kaabinejadian S., Hundal J., Petti A.A., Ly A., Lie W.R., Hildebrand W.H., Mardis E.R. (2015). Cancer immunotherapy. A dendritic cell vaccine increases the breadth and diversity of melanoma neoantigen-specific T cells. Science.

[182] Ding Z., Li Q., Zhang R., Xie L., Shu Y., Gao S., Wang P., Su X., Qin Y., Wang Y. (2021). Personalized neoantigen pulsed dendritic cell vaccine for advanced lung cancer. Signal Transduct. Target. Ther..

[183] Sarivalasis A., Boudousquié C., Balint K., Stevenson B.J., Gannon P.O., Iancu E.M., Rossier L., Martin Lluesma S., Mathevet P., Sempoux C. (2019). A Phase I/II trial comparing autologous dendritic cell vaccine pulsed either with personalized peptides (PEP-DC) or with tumor lysate (OC-DC) in patients with advanced high-grade ovarian serous carcinoma. J. Transl. Med..

[184] University Hospital, Ghent (2024). MIDRIXNEO-LUNG Dendritic Cell Vaccine in Patients With Non-small Cell Lung Cancer (MIDRIXNEO). https://www.clinicaltrials.gov/study/NCT04078269?cond=NCT04078269&rank=1.

[185] The First People’s Hospital of Lianyungang (2017). Neo-MASCT Immunotherapy for Advanced NSCLC. https://www.clinicaltrials.gov/study/NCT03205930?cond=NCT03205930&rank=1.

[186] Liang P., Chinese PLA General Hospital (2018). A Study Combining Personalized Neoantigen-based Dendritic Cell Vaccine With Microwave Ablation for the Treatment of Hepatocellular Carcinoma. https://www.clinicaltrials.gov/study/NCT03674073?cond=NCT03674073&rank=1.

[187] Batich K.A., Reap E.A., Archer G.E., Sanchez-Perez L., Nair S.K., Schmittling R.J., Norberg P., Xie W., Herndon J.E., Healy P. (2017). Long-term Survival in Glioblastoma with Cytomegalovirus pp65-Targeted Vaccination. Clin. Cancer Res..

[188] Han W., Chinese PLA General Hospital (2023). Combination of CAR-DC Vaccine and ICIs in Malignant Tumors. https://www.clinicaltrials.gov/study/NCT05631886?cond=NCT05631886&rank=1.

[189] Mao K., Tan H., Cong X., Liu J., Xin Y., Wang J., Guan M., Li J., Zhu G., Meng X. (2025). Optimized lipid nanoparticles enable effective CRISPR/Cas9-mediated gene editing in dendritic cells for enhanced immunotherapy. Acta Pharm. Sin. B.

[190] Cai Z., Wuri Q., Song Y., Qu X., Hu H., Cao S., Wu H., Wu J., Wang C., Yu X. (2025). CircRNA-loaded DC vaccine in combination with low-dose gemcitabine induced potent anti-tumor immunity in pancreatic cancer model. Cancer Immunol. Immunother..

[191] Maeng H.M., National Cancer Institute (NCI) (2022). Ad/HER2/Neu Dendritic Cell Cancer Vaccine Testing. https://clinicaltrials.gov/study/NCT01730118?cond=%20Adenovirus%20%20Cancer&lastUpdPost=2020-01-01_&page=5&rank=49.

[192] Second Affiliated Hospital of Guangzhou Medical University (2024). Anti-cancer DC Cell Vaccination to Treat Solid Tumors. https://www.clinicaltrials.gov/study/NCT06477614?cond=NCT06477614&rank=1.

[193] Han W., Chinese PLA General Hospital (2024). CAR-DC Vaccine and ICIs in Local Advanced/Metastatic Solid Tumors. https://www.clinicaltrials.gov/study/NCT05631899?cond=NCT05631899&rank=1.

[194] Shenzhen Geno-Immune Medical Institute (2024). Engineered Dendritic Cell Vaccines for Multiple Myeloma. https://www.clinicaltrials.gov/study/NCT06435910?cond=NCT06435910&rank=1.

[195] Ding Z., Sichuan University (2022). A Translational Study of Tumor Antigen-pulsed DC Vaccine for ESCC. https://www.clinicaltrials.gov/study/NCT05317325?cond=NCT05317325&rank=1.

[196] Istituto Romagnolo per lo Studio dei Tumori Dino Amadori IRST S.r.l. IRCCS (2024). Vaccination with Autologous Dendritic Cells Loaded with Autologous Tumour Homogenate in Glioblastoma (Combi G-Vax). https://www.clinicaltrials.gov/study/NCT04523688?cond=NCT04523688&rank=1.

[197] Zhang Y., Beijing Tiantan Hospital (2024). Safety and Efficacy Study for DC Vaccine in Recurrent or Progressive High-grade Gliomas. https://www.clinicaltrials.gov/study/NCT06253234?cond=NCT06253234&rank=1.

[198] Ding Z., Sichuan University (2022). Personalized Immune Cell Therapy Targeting Neoantigen of Malignant Solid Tumors. https://www.clinicaltrials.gov/study/NCT05235607?cond=NCT05235607&rank=1.

[199] The First Affiliated Hospital of Nanchang University (2023). Using Neoantigen Peptide Vaccine/Neoantigen-based DC to Treat Advanced Malignant Solid Tumors. https://www.clinicaltrials.gov/study/NCT05749627?cond=NCT05749627&rank=1.

[200] Yin R., West China Second University Hospital (2022). Dendritic Cell Vaccination With Standard Postoperative Chemotherapy for the Treatment of Adult Ovarian Cancer. https://www.clinicaltrials.gov/study/NCT05270720?cond=NCT05270720&rank=1.

[201] Shenzhen People’s Hospital (2019). DC Vaccines Targeting HPV16/18 E6/E7 Protein to Regress CINI/CIN2. https://www.clinicaltrials.gov/study/NCT03870113?cond=NCT03870113&rank=1.

[202] Roswell Park Cancer Institute (2024). Dendritic Cell Vaccines Against Her2/Her3 and Pembrolizumab for the Treatment of Brain Metastasis From Triple Negative Breast Cancer or HER2+ Breast Cancer. https://www.clinicaltrials.gov/study/NCT04348747?cond=NCT04348747&rank=1.

[203] University of Nebraska (2023). Neoadjuvant/Adjuvant Chemotherapy, Vaccine & Adjuvant Radiation Therapy in p53-Overexpressing Stage III Breast Cancer. https://clinicaltrials.gov/study/NCT00082641?cond=%20Adenovirus%20%20Cancer&lastUpdPost=2020-01-01_&page=4&rank=33.

[204] University of Florida (2025). Adoptive T Cell Therapy, DC Vaccines, and Hematopoietic Stem Cells Combined With Immune checkPOINT Blockade in Patients With Medulloblastoma (MATCHPOINT). https://www.clinicaltrials.gov/study/NCT06514898?cond=NCT06514898&rank=1.

[205] Shenzhen Geno-Immune Medical Institute (2022). NGS-MRD Assessment of Combination Immunotherapies Targeting B-ALL. https://www.clinicaltrials.gov/study/NCT05262673?cond=NCT05262673&rank=1.

[206] Shanghai Humantech Biotechnology Co. Ltd. (2023). Safety of Prodencel in the Treatment of Metastatic Castration-resistant Prostate Cancer (mCRPC). https://www.clinicaltrials.gov/study/NCT05533203?cond=NCT05533203&rank=1.

[207] Shenzhen Geno-Immune Medical Institute (2022). NGS-MRD Assessment of Combination Immunotherapies Targeting T-ALL. https://www.clinicaltrials.gov/study/NCT05277753?cond=NCT05277753&rank=1.

[208] Universidad Nacional de Colombia (2024). Personalized Vaccine for TNBC Immunotherapy (TEBICA003 TNBC). https://www.clinicaltrials.gov/study/NCT06195618?cond=NCT06195618&rank=1.

[209] H. Lee Moffitt Cancer Center and Research Institute (2025). Adoptive T Cell Therapy Following HER2-Pulsed Dendritic Cell Vaccine & Pepinemab /Trastuzumab in Patients w/ Metastatic HER2+ Breast Cancer. https://www.clinicaltrials.gov/study/NCT05378464?cond=NCT05378464&rank=1.

[210] Song E., Sun Yat-Sen Memorial Hospital of Sun Yat-Sen University (2024). A Single Arm Clinical Study of Dendritic Cell Vaccine Loaded With Circular RNA Encoding Cryptic Peptide for Patients With HER2-negative Advanced Breast Cancer. https://www.clinicaltrials.gov/study/NCT06530082?cond=NCT06530082&rank=1.

[211] Joos G., University Hospital, Ghent (2021). MIDRIX4-LUNG Dendritic Cell Vaccine in Patients With Metastatic Non-small Cell Lung Cancer (MIDRIX4-LUNG). https://www.clinicaltrials.gov/study/NCT04082182?cond=NCT04082182&rank=1.

[212] Instituto Oncológico Dr Rosell (2024). Combination of Atezolizumab With Dendritic Cell Vaccine in Patients With Lung Cancer (VENEZO-LUNG). https://www.clinicaltrials.gov/study/NCT04487756?cond=NCT04487756&rank=1.

[213] Istituto Romagnolo per lo Studio dei Tumori Dino Amadori IRST S.r.l. IRCCS (2025). Sequential Immunochemotherapy Treatment with Pembrolizumab Plus Dendritic Cell (DC) Vaccine Followed by Trifluridine/Tipiracil Plus Bevacizumab in Refractory Mismatch-repair-proficient (pMMR) or Microsatellite-stable (MSS) Metastatic Colorectal Cancer (CombiCoR-Vax). https://www.clinicaltrials.gov/study/NCT06522919?cond=NCT06522919&rank=1.

[214] Digklia A., Centre Hospitalier Universitaire Vaudois (2022). Personalized Vaccine With SOC Chemo Followed by Nivo in Pancreatic Cancer. https://www.clinicaltrials.gov/study/NCT04627246?cond=NCT04627246&rank=1.

[215] H. Lee Moffitt Cancer Center and Research Institute (2024). Study of HER2 Directed Dendritic Cell (DC1) Vaccine + Weekly Paclitaxel, Trastuzumab & Pertuzumab. https://www.clinicaltrials.gov/study/NCT05325632?cond=NCT05325632&rank=1.

[216] Nijman H.W., University Medical Center Groningen (2024). Phase 1 Study to Evaluate the Safety, Feasibility and Immunogenicity of an Allogeneic, Cell-based Vaccine (DCP-001) in High Grade Serous Ovarian Cancer Patients After Primary Treatment (ALISON). https://www.clinicaltrials.gov/study/NCT04739527?cond=NCT04739527&rank=1.

[217] Istituto Romagnolo per lo Studio dei Tumori Dino Amadori IRST S.r.l. IRCCS (2024). Pembrolizumab Plus Autologous Dendritic Cell Vaccine in Patients with PD-L1 Negative Advanced Mesothelioma Who Have Failed Prior Therapies. https://clinicaltrials.gov/study/NCT03546426?cond=NCT03546426&rank=1.

[218] National Institutes of Health Clinical Center (CC) (National Cancer Institute (NCI)) (2025). Pembrolizumab, Lenvatinib and IL-15 Superagonist N-803 in Combination With HER2 Targeting Autologous Dendritic Cell (AdHER2DC) Vaccine in Participants With Advanced or Metastatic Endometrial Cancer. https://clinicaltrials.gov/study/NCT06253494?cond=NCT06253494&rank=1.

[219] Mayo Clinic (2024). Modified Immune Cells (Autologous Dendritic Cells) and a Vaccine (Prevnar) After High-Dose External Beam Radiation Therapy in Treating Patients With Unresectable Liver Cancer. https://clinicaltrials.gov/study/NCT03942328?cond=NCT03942328&rank=1.

[220] Huang Y., Third Affiliated Hospital, Sun Yat-Sen University (2020). “Cocktail” Therapy for Hepatitis B Related Hepatocellular Carcinoma. https://clinicaltrials.gov/study/NCT04317248?cond=NCT04317248&rank=1.

[221] Maranchie J., University of Pittsburgh (2024). Autologous Dendritic Cell Vaccine in Kidney Cancer. https://clinicaltrials.gov/study/NCT05127824?cond=NCT05127824&rank=1.

[222] Jonsson Comprehensive Cancer Center (2025). CCL21-Gene Modified Dendritic Cell Vaccine and Pembrolizumab in Treating Patients With Stage IV Non-small Cell Lung Cancer. https://clinicaltrials.gov/study/NCT03546361?cond=NCT03546361&rank=1.

[223] H. Lee Moffitt Cancer Center and Research Institute (2022). Immune Response and Potential Booster for Patients Who Have Received HER2-pulsed DC1. https://clinicaltrials.gov/study/NCT03630809?cond=NCT03630809&rank=1.

[224] Rosenblatt J., Beth Israel Deaconess Medical Center (2023). Dendritic Cell (DC)/Myeloma Fusions in Combination With Nivolumab in Patients With Relapsed Multiple Myeloma. https://clinicaltrials.gov/study/NCT03782064?cond=NCT03782064&rank=1.

[225] PDC*line Pharma SAS (2024). Safety, Immunogenicity and Preliminary Clinical Activity Study of PDC*lung01 Cancer Vaccine in NSCLC. https://clinicaltrials.gov/study/NCT03970746?cond=NCT03970746&rank=1.

[226] Jonsson Comprehensive Cancer Center (2024). Pembrolizumab and a Vaccine (ATL-DC) for the Treatment of Surgically Accessible Recurrent Glioblastoma. https://clinicaltrials.gov/study/NCT04201873?cond=NCT04201873&rank=1.

[227] Ding Z., Sichuan University (2025). Tumor Antigen-sensitized DC Vaccine As an Adjuvant Therapy for Esophagus Cancer. https://clinicaltrials.gov/study/NCT05023928?cond=NCT05023928&rank=1.

[228] Affiliated Hospital to Academy of Military Medical Sciences (2021). Clinical Study of DC-AML Cells in the Treatment of Acute Myeloid Leukemia. https://clinicaltrials.gov/study/NCT05000801?cond=NCT05000801&rank=1.

[229] Ding Z., Sichuan University (2024). DC Combined With ICIs in the Treatment of Advanced Lung Cancer Resistant to ICIs. https://clinicaltrials.gov/study/NCT06329908?cond=NCT06329908&rank=1.

[230] Zhang Y., Beijing Tiantan Hospital (2025). Safety & Efficacy of DC Vaccine and TMZ for the Treatment of Newly-diagnosed Glioblastoma After Surgery. https://clinicaltrials.gov/study/NCT04968366?cond=NCT04968366&rank=1.

[231] National Cancer Centre, Singapore (2024). Neoantigen Dendritic Cell Vaccine and Nivolumab in HCC and Liver Metastases From CRC. https://clinicaltrials.gov/study/NCT04912765?cond=NCT04912765&rank=1.

[232] National Health Research Institutes, Taiwan (2023). Neoantigen Derived DCs as Cancer Treatment. https://clinicaltrials.gov/study/NCT05767684?cond=NCT05767684&rank=1.

[233] Frame Pharmaceuticals B.V. (2021). FRAME-001 Personalized Vaccine in NSCLC. https://clinicaltrials.gov/study/NCT04998474?cond=NCT04998474&rank=1.

[234] Keenan B.P., Jaffee E.M. (2012). Whole cell vaccines--past progress and future strategies. Semin. Oncol..

[235] Chang M.C., Chen Y.L., Chiang Y.C., Chen T.C., Tang Y.C., Chen C.A., Sun W.Z., Cheng W.F. (2016). Mesothelin-specific cell-based vaccine generates antigen-specific immunity and potent antitumor effects by combining with IL-12 immunomodulator. Gene Ther..

[236] Sakamoto C., Kohara H., Inoue H., Narusawa M., Ogawa Y., Hirose-Yotsuya L., Miyamoto S., Matsumura Y., Yamada K., Takahashi A. (2017). Therapeutic vaccination based on side population cells transduced by the granulocyte-macrophage colony-stimulating factor gene elicits potent antitumor immunity. Cancer Gene Ther..

[237] Kayaga J., Souberbielle B.E., Sheikh N., Morrow W.J., Scott-Taylor T., Vile R., Chong H., Dalgleish A.G. (1999). Anti-tumour activity against B16-F10 melanoma with a GM-CSF secreting allogeneic tumour cell vaccine. Gene Ther..

[238] Dranoff G., Jaffee E., Lazenby A., Golumbek P., Levitsky H., Brose K., Jackson V., Hamada H., Pardoll D., Mulligan R.C. (1993). Vaccination with irradiated tumor cells engineered to secrete murine granulocyte-macrophage colony-stimulating factor stimulates potent, specific, and long-lasting anti-tumor immunity. Proc. Natl. Acad. Sci. USA.

[239] Sidney Kimmel Comprehensive Cancer Center at Johns Hopkins (2021). GVAX Pancreas Vaccine (With CY) and CRS-207 With or Without Nivolumab. https://clinicaltrials.gov/study/NCT02243371?cond=GVAX&rank=5.

[240] Sidney Kimmel Comprehensive Cancer Center at Johns Hopkins (2025). Pilot Study With CY, Pembrolizumab, GVAX, and IMC-CS4 (LY3022855) in Patients With Borderline Resectable Adenocarcinoma of the Pancreas. https://clinicaltrials.gov/study/NCT03153410?cond=GVAX&rank=7.

[241] Sidney Kimmel Comprehensive Cancer Center at Johns Hopkins (2023). Adjuvant GVAX Vaccine Therapy in Patients With Pancreatic Cancer. https://clinicaltrials.gov/study/NCT00389610?cond=GVAX&page=2&rank=14.

[242] Cell Genesys (2005). Vaccination Priming and Vaccine Boosting Trial of Allogeneic Human GM-CSF Gene Transduced Irradiated Prostate Cancer Cell Vaccines (GVAX® Vaccine for Prostate Cancer). https://clinicaltrials.gov/study/NCT00140374?cond=GVAX&page=3&rank=24.

[243] Collins N., Dana-Farber Cancer Institute (2024). GVAX Plus Checkpoint Blockade in Neuroblastoma. https://clinicaltrials.gov/study/NCT04239040?cond=GVAX&rank=1.

[244] Sidney Kimmel Comprehensive Cancer Center at Johns Hopkins (2021). Study of GVAX (With CY) and Pembrolizumab in MMR-p Advanced Colorectal Cancer. https://clinicaltrials.gov/study/NCT02981524?cond=GVAX&page=2&rank=19.

[245] Guo C., Manjili M.H., Subjeck J.R., Sarkar D., Fisher P.B., Wang X.Y. (2013). Therapeutic cancer vaccines: Past, present, and future. Adv. Cancer Res..

[246] Carri I., Schwab E., Trivino J.C., von Euw E.M., Nielsen M., Mordoh J., Barrio M.M. (2024). VACCIMEL, an allogeneic melanoma vaccine, efficiently triggers T cell immune responses against neoantigens and alloantigens, as well as against tumor-associated antigens. Front. Immunol..

[247] Mordoh J., Pampena M.B., Aris M., Blanco P.A., Lombardo M., von Euw E.M., Mac Keon S., Yépez Crow M., Bravo A.I., O’Connor J.M. (2017). Phase II Study of Adjuvant Immunotherapy with the CSF-470 Vaccine Plus Bacillus Calmette-Guerin Plus Recombinant Human Granulocyte Macrophage-Colony Stimulating Factor vs Medium-Dose Interferon Alpha 2B in Stages IIB, IIC, and III Cutaneous Melanoma Patients: A Single Institution, Randomized Study. Front. Immunol..

[248] Mordoh A., Aris M., Carri I., Bravo A.I., Podaza E., Pardo J.C.T., Cueto G.R., Barrio M.M., Mordoh J. (2022). An Update of Cutaneous Melanoma Patients Treated in Adjuvancy With the Allogeneic Melanoma Vaccine VACCIMEL and Presentation of a Selected Case Report With In-Transit Metastases. Front. Immunol..

[249] Podaza E., Carri I., Aris M., von Euw E., Bravo A.I., Blanco P., Ortiz Wilczyñski J.M., Koile D., Yankilevich P., Nielsen M. (2020). Evaluation of T-Cell Responses Against Shared Melanoma Associated Antigens and Predicted Neoantigens in Cutaneous Melanoma Patients Treated With the CSF-470 Allogeneic Cell Vaccine Plus BCG and GM-CSF. Front. Immunol..

[250] Pampena M.B., Cartar H.C., Cueto G.R., Levy E.M., Blanco P.A., Barrio M.M., Mordoh J. (2018). Dissecting the Immune Stimulation Promoted by CSF-470 Vaccine Plus Adjuvants in Cutaneous Melanoma Patients: Long Term Antitumor Immunity and Short Term Release of Acute Inflammatory Reactants. Front. Immunol..

[251] Mayo Clinic Vaccine Therapy in Treating Patients With Recurrent Glioblastoma. https://clinicaltrials.gov/study/NCT03360708?cond=NCT03360708&rank=1.

[252] OX2 Therapeutics Study of CD200 Activation Receptor Ligand (CD200AR-L) and Allogeneic Tumor Lysate Vaccine Immunotherapy for Recurrent Glioblastoma. https://clinicaltrials.gov/study/NCT04642937?cond=NCT04642937&rank=1.

[253] OX2 Therapeutics CD200AR-L and Allogeneic Tumor Lysate Vaccine Immunotherapy for Recurrent HGG and Newly Diagnosed DMG/DIPG in Children and Young Adults. https://clinicaltrials.gov/study/NCT06305910?cond=NCT06305910&rank=1.

[254] Second Affiliated Hospital, School of Medicine, Zhejiang University Clinical Study of an Dendritic and Glioma Cells Fusion Vaccine With IL-12 for Treatment-naïve GBM Patients. https://clinicaltrials.gov/study/NCT04388033?cond=NCT04388033&rank=1.

[255] Sidney Kimmel Comprehensive Cancer Center at Johns Hopkins (2025). GVAX Pancreas Vaccine (With CY) in Combination With Nivolumab and SBRT for Patients With Borderline Resectable Pancreatic Cancer. https://clinicaltrials.gov/study/NCT03161379?cond=GVAX&rank=8.

[256] Sidney Kimmel Comprehensive Cancer Center at Johns Hopkins (2024). Study of CRS-207, Nivolumab, and Ipilimumab With or Without GVAX Pancreas Vaccine (With Cy) in Patients With Pancreatic Cancer. https://clinicaltrials.gov/study/NCT03190265?cond=GVAX&rank=9.

[257] Sidney Kimmel Comprehensive Cancer Center at Johns Hopkins (2024). Pancreatic Tumor Cell Vaccine (GVAX), Cyclophosphamide, SBRT, and FOLFIRINOX in Patients With Resected Adenocarcinoma of the Pancreas. https://clinicaltrials.gov/study/NCT01595321?cond=GVAX&page=2&rank=11.

[258] Sidney Kimmel Comprehensive Cancer Center at Johns Hopkins (2024). Study With CY, Pembrolizumab, GVAX Pancreas Vaccine, and SBRT in Patients With Locally Advanced Pancreatic Cancer. https://clinicaltrials.gov/study/NCT02648282?cond=GVAX&page=3&rank=28.

[259] Ho V.T., Dana-Farber Cancer Institute (2022). GVAX vs. Placebo for MDS/AML After Allo HSCT. https://clinicaltrials.gov/study/NCT01773395?cond=GVAX&page=2&rank=12.

[260] Ho V.T., Dana-Farber Cancer Institute (2022). GM-CSF Vaccinations After Allogeneic Blood Stem Cell Transplantation in Patients With Advanced Myeloid Malignancies. https://clinicaltrials.gov/study/NCT00426205?cond=GVAX&page=4&rank=32.

[261] Sidney Kimmel Comprehensive Cancer Center at Johns Hopkins (2024). Allogeneic Myeloma GM-CSF Vaccine With Lenalidomide in Multiple Myeloma Patients in Complete or Near Complete Remission. https://clinicaltrials.gov/study/NCT03376477?cond=GVAX&page=4&rank=34.

[262] Sidney Kimmel Comprehensive Cancer Center at Johns Hopkins (2021). SGI-110 in Combination With an Allogeneic Colon Cancer Cell Vaccine (GVAX) and Cyclophosphamide (CY) in Metastatic Colorectal Cancer (mCRC). https://clinicaltrials.gov/study/NCT01966289?cond=GVAX&page=3&rank=23.

[263] Sidney Kimmel Comprehensive Cancer Center at Johns Hopkins (2019). Study of Colon GVAX and Cyclophosphamide in Patients With Metastatic Colorectal Cancer. https://clinicaltrials.gov/study/NCT00656123?cond=GVAX&page=3&rank=26.

[264] Sidney Kimmel Comprehensive Cancer Center at Johns Hopkins (2019). A Neoadjuvant Study of Androgen Ablation Combined With Cyclophosphamide and GVAX Vaccine for Localized Prostate Cancer. https://clinicaltrials.gov/study/NCT01696877?cond=GVAX&page=3&rank=29.

[265] Garrido-Castro A.C., Dana-Farber Cancer Institute (2022). Vaccination With Autologous Breast Cancer Cells Engineered to Secrete Granulocyte-Macrophage Colony-Stimulating Factor (GM-CSF) in Metastatic Breast Cancer Patients. https://clinicaltrials.gov/study/NCT00317603?cond=%20Adenovirus%20%20Cancer&lastUpdPost=2020-01-01_&page=7&rank=63.

[266] Garrido-Castro A.C., Dana-Farber Cancer Institute (2022). Autologous Vaccination With Lethally Irradiated, Autologous Breast Cancer Cells Engineered to Secrete GM-CSF in Women With Operable Breast Cancer. https://clinicaltrials.gov/study/NCT00880464?cond=%20Adenovirus%20%20Cancer&lastUpdPost=2020-01-01_&page=7&rank=61.

[267] Gritstone bio, Inc. A Study of a Personalized Neoantigen Cancer Vaccine. https://clinicaltrials.gov/study/NCT03639714?cond=NCT03639714&rank=1.

[268] Oxford Vacmedix UK Ltd. First-in-human Study of OVM-200 As a Therapeutic Cancer Vaccine. https://clinicaltrials.gov/study/NCT05104515?cond=NCT05104515&rank=1.

[269] Cancer Vaccines Limited Investigation of a Therapeutic Vaccine (ACIT-1) in Cancer. https://clinicaltrials.gov/study/NCT03096093?cond=NCT03096093&rank=1.

[270] Hu Z., Leet D.E., Allesøe R.L., Oliveira G., Li S., Luoma A.M., Liu J., Forman J., Huang T., Iorgulescu J.B. (2021). Personal neoantigen vaccines induce persistent memory T cell responses and epitope spreading in patients with melanoma. Nat. Med..

[271] Ott P.A., Hu Z., Keskin D.B., Shukla S.A., Sun J., Bozym D.J., Zhang W., Luoma A., Giobbie-Hurder A., Peter L. (2017). An immunogenic personal neoantigen vaccine for patients with melanoma. Nature.

[272] Latzer P., Zelba H., Battke F., Reinhardt A., Shao B., Bartsch O., Rabsteyn A., Harter J., Schulze M., Okech T. (2024). A real-world observation of patients with glioblastoma treated with a personalized peptide vaccine. Nat. Commun..

[273] Shi Y., Liu C.H., Roberts A.I., Das J., Xu G., Ren G., Zhang Y., Zhang L., Yuan Z.R., Tan H.S. (2006). Granulocyte-macrophage colony-stimulating factor (GM-CSF) and T-cell responses: What we do and don’t know. Cell Res..

[274] Toubaji A., Hill S., Terabe M., Qian J., Floyd T., Simpson R.M., Berzofsky J.A., Khleif S.N. (2007). The combination of GM-CSF and IL-2 as local adjuvant shows synergy in enhancing peptide vaccines and provides long term tumor protection. Vaccine.

[275] O’Shea A.E., Clifton G.T., Qiao N., Heckman-Stoddard B.M., Wojtowicz M., Dimond E., Bedrosian I., Weber D., Garber J.E., Husband A. (2023). Phase II Trial of Nelipepimut-S Peptide Vaccine in Women with Ductal Carcinoma In Situ. Cancer Prev. Res..

[276] Klinman D.M. (2004). Immunotherapeutic uses of CpG oligodeoxynucleotides. Nat. Rev. Immunol..

[277] Hemmi H., Takeuchi O., Kawai T., Kaisho T., Sato S., Sanjo H., Matsumoto M., Hoshino K., Wagner H., Takeda K. (2000). A Toll-like receptor recognizes bacterial DNA. Nature.

[278] Takeshita F., Leifer C.A., Gursel I., Ishii K.J., Takeshita S., Gursel M., Klinman D.M. (2001). Cutting edge: Role of Toll-like receptor 9 in CpG DNA-induced activation of human cells. J. Immunol..

[279] Weiner G.J., Liu H.M., Wooldridge J.E., Dahle C.E., Krieg A.M. (1997). Immunostimulatory oligodeoxynucleotides containing the CpG motif are effective as immune adjuvants in tumor antigen immunization. Proc. Natl. Acad. Sci. USA.

[280] Lipford G.B., Bauer M., Blank C., Reiter R., Wagner H., Heeg K. (1997). CpG-containing synthetic oligonucleotides promote B and cytotoxic T cell responses to protein antigen: A new class of vaccine adjuvants. Eur. J. Immunol..

[281] Oxenius A., Martinic M.M., Hengartner H., Klenerman P. (1999). CpG-containing oligonucleotides are efficient adjuvants for induction of protective antiviral immune responses with T-cell peptide vaccines. J. Virol..

[282] Vabulas R.M., Pircher H., Lipford G.B., Häcker H., Wagner H. (2000). CpG-DNA activates in vivo T cell epitope presenting dendritic cells to trigger protective antiviral cytotoxic T cell responses. J. Immunol..

[283] Davila E., Celis E. (2000). Repeated administration of cytosine-phosphorothiolated guanine-containing oligonucleotides together with peptide/protein immunization results in enhanced CTL responses with anti-tumor activity. J. Immunol..

[284] Speiser D.E., Liénard D., Rufer N., Rubio-Godoy V., Rimoldi D., Lejeune F., Krieg A.M., Cerottini J.C., Romero P. (2005). Rapid and strong human CD8+ T cell responses to vaccination with peptide, IFA, and CpG oligodeoxynucleotide 7909. J. Clin. Investig..

[285] Tarhini A., University of Pittsburgh (2017). Vaccine Therapy in Treating Patients With Recurrent Stage III or Stage IV Melanoma That Cannot Be Removed by Surgery. https://clinicaltrials.gov/study/NCT00471471?cond=cpg%20PLUS%20peptide%20vaccination&rank=3.

[286] Fourcade J., Kudela P., Andrade Filho P.A., Janjic B., Land S.R., Sander C., Krieg A., Donnenberg A., Shen H., Kirkwood J.M. (2008). Immunization with analog peptide in combination with CpG and montanide expands tumor antigen-specific CD8+ T cells in melanoma patients. J. Immunother..

[287] Michielin O., Centre Hospitalier Universitaire Vaudois (2020). Immunotherapy of Stage III/IV Melanoma Patients. https://clinicaltrials.gov/study/NCT00112242?cond=cpg%20PLUS%20peptide%20vaccination&rank=5.

[288] Michielin O., Centre Hospitalier Universitaire Vaudois (2013). Immunotherapy of HLA-A2 Positive Stage III/IV Melanoma Patients. https://clinicaltrials.gov/study/NCT00112229?cond=cpg%20PLUS%20peptide%20vaccination&rank=6.

[289] Fred Hutchinson Cancer Center (2024). Personalized Neo-Antigen Peptide Vaccine for the Treatment of Stage IIIC-IV Melanoma or Hormone Receptor Positive Her2 Negative Metastatic Refractory Breast Cancer. https://clinicaltrials.gov/study/NCT05098210?cond=NCT05098210&rank=1.

[290] Marker Therapeutics, Inc. (2021). Folate Receptor Alpha Peptide Vaccine With GM-CSF in Patients With Triple Negative Breast Cancer. https://clinicaltrials.gov/study/NCT02593227?cond=NCT02593227&rank=1.

[291] Greenwich LifeSciences, Inc. (2025). Phase 3 Study to Evaluate the Efficacy and Safety of HER2/neu Peptide GLSI-100 (GP2 + GM-CSF) in HER2/neu Positive Subjects (FLAMINGO-01). https://clinicaltrials.gov/study/NCT05232916?cond=GP2&rank=1#study-plan.

[292] Academic and Community Cancer Research United (2023). Multi-epitope Folate Receptor Alpha Peptide Vaccine, GM-CSF, and Cyclophosphamide in Treating Patients With Triple Negative Breast Cancer. https://clinicaltrials.gov/study/NCT03012100?cond=NCT03012100&rank=1.

[293] Centre Hospitalier Universitaire de Besancon (2025). Universal Cancer Peptide-based Vaccination in Metastatic NSCLC (UCPVax). https://clinicaltrials.gov/study/NCT02818426?cond=NCT02818426&rank=1.

[294] BioNTech SE (BioNTech US Inc.) (2021). A Personal Cancer Vaccine (NEO-PV-01) With Pembrolizumab and Chemotherapy for Patients With Lung Cancer. https://clinicaltrials.gov/study/NCT03380871?cond=NCT03380871&rank=1.

[295] Daigo Y., Shiga University (2019). Safety and Efficacy Study of Epitope Peptide To Treat HLA-A*02 Disease Controlled Advanced Non-small Cell Lung Cancer. https://clinicaltrials.gov/study/NCT01949701?cond=HLA-A*24:02&rank=2.

[296] OSE Immunotherapeutics (2025). Trial of Therapeutic Cancer Vaccine OSE2101 in Patients With Non-Small Cell Lung Cancer and Secondary Resistance to Immune Checkpoint Inhibitor (ARTEMIA). https://clinicaltrials.gov/study/NCT06472245?cond=NCT06472245&rank=1.

[297] Peters K., Duke University (2023). IDH1 Peptide Vaccine for Recurrent Grade II Glioma (RESIST). https://clinicaltrials.gov/study/NCT02193347?cond=NCT02193347&rank=1.

[298] Sidney Kimmel Comprehensive Cancer Center at Johns Hopkins (2024). Mutant KRAS -Targeted Long Peptide Vaccine for Patients at High Risk of Developing Pancreatic Cancer. https://clinicaltrials.gov/study/NCT05013216?cond=NCT05013216&rank=1.

[299] Lynn G.M., Sedlik C., Baharom F., Zhu Y., Ramirez-Valdez R.A., Coble V.L., Tobin K., Nichols S.R., Itzkowitz Y., Zaidi N. (2020). Peptide-TLR-7/8a conjugate vaccines chemically programmed for nanoparticle self-assembly enhance CD8 T-cell immunity to tumor antigens. Nat. Biotechnol..

[300] Kuai R., Ochyl L.J., Bahjat K.S., Schwendeman A., Moon J.J. (2017). Designer vaccine nanodiscs for personalized cancer immunotherapy. Nat. Mater..

[301] Ni Q., Zhang F., Liu Y., Wang Z., Yu G., Liang B., Niu G., Su T., Zhu G., Lu G. (2020). A bi-adjuvant nanovaccine that potentiates immunogenicity of neoantigen for combination immunotherapy of colorectal cancer. Sci. Adv..

[302] Zhu G., Lynn G.M., Jacobson O., Chen K., Liu Y., Zhang H., Ma Y., Zhang F., Tian R., Ni Q. (2017). Albumin/vaccine nanocomplexes that assemble in vivo for combination cancer immunotherapy. Nat. Commun..

[303] Zhang Y., Jiang L., Huang S., Lian C., Liang H., Xing Y., Liu J., Tian X., Liu Z., Wang R. (2024). Sulfonium-Stapled Peptides-Based Neoantigen Delivery System for Personalized Tumor Immunotherapy and Prevention. Adv. Sci..

[304] Lai C., Duan S., Ye F., Hou X., Li X., Zhao J., Yu X., Hu Z., Tang Z., Mo F. (2018). The enhanced antitumor-specific immune response with mannose- and CpG-ODN-coated liposomes delivering TRP2 peptide. Theranostics.

[305] Liang Z., Cui X., Yang L., Hu Q., Li D., Zhang X., Han L., Shi S., Shen Y., Zhao W. (2021). Co-assembled nanocomplexes of peptide neoantigen Adpgk and Toll-like receptor 9 agonist CpG ODN for efficient colorectal cancer immunotherapy. Int. J. Pharm..

[306] Zhu G., Mei L., Vishwasrao H.D., Jacobson O., Wang Z., Liu Y., Yung B.C., Fu X., Jin A., Niu G. (2017). Intertwining DNA-RNA nanocapsules loaded with tumor neoantigens as synergistic nanovaccines for cancer immunotherapy. Nat. Commun..

[307] Cai Z., Xin F., Wei Z., Wu M., Lin X., Du X., Chen G., Zhang D., Zhang Z., Liu X. (2020). Photodynamic Therapy Combined with Antihypoxic Signaling and CpG Adjuvant as an In Situ Tumor Vaccine Based on Metal-Organic Framework Nanoparticles to Boost Cancer Immunotherapy. Adv. Healthc. Mater..

[308] Liu H., Chen H., Liu Z., Le Z., Nie T., Qiao D., Su Y., Mai H., Chen Y., Liu L. (2020). Therapeutic nanovaccines sensitize EBV-associated tumors to checkpoint blockade therapy. Biomaterials.

[309] Scheetz L., Kadiyala P., Sun X., Son S., Hassani Najafabadi A., Aikins M., Lowenstein P.R., Schwendeman A., Castro M.G., Moon J.J. (2020). Synthetic High-density Lipoprotein Nanodiscs for Personalized Immunotherapy Against Gliomas. Clin. Cancer Res..

[310] Ahluwalia M.S., Reardon D.A., Abad A.P., Curry W.T., Wong E.T., Figel S.A., Mechtler L.L., Peereboom D.M., Hutson A.D., Withers H.G. (2023). Phase IIa Study of SurVaxM Plus Adjuvant Temozolomide for Newly Diagnosed Glioblastoma. J. Clin. Oncol..

[311] Stupp R., Mason W.P., van den Bent M.J., Weller M., Fisher B., Taphoorn M.J., Belanger K., Brandes A.A., Marosi C., Bogdahn U. (2005). Radiotherapy plus concomitant and adjuvant temozolomide for glioblastoma. N. Engl. J. Med..

[312] Stupp R., Taillibert S., Kanner A., Read W., Steinberg D., Lhermitte B., Toms S., Idbaih A., Ahluwalia M.S., Fink K. (2017). Effect of Tumor-Treating Fields Plus Maintenance Temozolomide vs Maintenance Temozolomide Alone on Survival in Patients With Glioblastoma: A Randomized Clinical Trial. Jama.

[313] Hodi F.S., O’Day S.J., McDermott D.F., Weber R.W., Sosman J.A., Haanen J.B., Gonzalez R., Robert C., Schadendorf D., Hassel J.C. (2010). Improved survival with ipilimumab in patients with metastatic melanoma. N. Engl. J. Med..

[314] Ott P.A., Hu-Lieskovan S., Chmielowski B., Govindan R., Naing A., Bhardwaj N., Margolin K., Awad M.M., Hellmann M.D., Lin J.J. (2020). A Phase Ib Trial of Personalized Neoantigen Therapy Plus Anti-PD-1 in Patients with Advanced Melanoma, Non-small Cell Lung Cancer, or Bladder Cancer. Cell.

[315] Cure&Sure Biotech Co., LTD. (2019). GP96 Heat Shock Protein-Peptide Complex Vaccine in Treating Patients With Liver Cancer. https://clinicaltrials.gov/study/NCT04206254?cond=NCT04206254&rank=1.

[316] Ashour Z.A.Y.H., Ain Shams University (2022). Personalized Cancer Vaccine in Egyptian Cancer Patients (PROVE). https://clinicaltrials.gov/study/NCT05059821?cond=NCT05059821&rank=1.

[317] Elicio Therapeutics (2024). A Study of ELI-002 7P in Subjects With KRAS/NRAS Mutated Solid Tumors (AMPLIFY-7P). https://clinicaltrials.gov/study/NCT05726864?cond=NCT05726864&rank=1.

[318] Elicio Therapeutics (2025). A Study of ELI-002 in Subjects With KRAS Mutated Pancreatic Ductal Adenocarcinoma (PDAC) and Other Solid Tumors (AMPLIFY-201). https://clinicaltrials.gov/study/NCT04853017?cond=NCT04853017&rank=1.

[319] Zhang L., Sun Yat-sen University (2020). Phase I Study of Individualized Neoantigen Peptides in the Treatment of EGFR Mutant Non-small Cell Lung Cancer. https://clinicaltrials.gov/study/NCT04397926?cond=NCT04397926&rank=1.

[320] Svane I.M., Herlev Hospital (2023). Arginase-1 Peptide Vaccine in Patients With Metastatic Solid Tumors. https://clinicaltrials.gov/study/NCT03689192?cond=NCT03689192&rank=1.

[321] Daigo Y., Shiga University (2019). Safety and Efficacy Study of Epitope Peptide To Treat HLA-A*24 or A*02-positive Advanced Solid Tumors. https://clinicaltrials.gov/study/NCT01949688?cond=HLA-A*24:02&rank=1.

[322] Knudsen L.M., Herlev Hospital (2020). Vaccination With PD-L1 Peptide Against Multiple Myeloma. https://clinicaltrials.gov/study/NCT03042793?cond=NCT03042793&rank=1.

[323] Fang Y., Sir Run Run Shaw Hospital (2021). Clinical Study of a Personalized Neoantigen Cancer Vaccine in Treating Patients With Advanced Malignant Tumor. https://clinicaltrials.gov/study/NCT03662815?cond=NCT03662815&rank=1.

[324] Yang L., Zhejiang Provincial People’s Hospital (2021). Clinical Study of a Personalized Neoantigen Cancer Vaccine in Treating Patients With Advanced Pancreatic Cancer. https://clinicaltrials.gov/study/NCT03645148?cond=NCT03645148&rank=1.

[325] BioNTech SE (BioNTech US Inc.) (2021). A Personal Cancer Vaccine (NEO-PV-01) w/ Nivolumab for Patients With Melanoma, Lung Cancer or Bladder Cancer. https://clinicaltrials.gov/study/NCT02897765?cond=NCT02897765&rank=1.

[326] Ott P., Dana-Farber Cancer Institute (2024). A Phase I Study with a Personalized NeoAntigen Cancer Vaccine in Melanoma. https://clinicaltrials.gov/study/NCT01970358?cond=NCT01970358&rank=1.

[327] NuGenerex Immuno-Oncology (2021). Establishing the Recommended Biological Dose for AE37 Peptide Vaccine in Combination With Pembrolizumab That Will Enhance the Tumor-specific Immune Response and Demonstrate Efficacy in Patients With Advanced Triple-negative Breast Cancer (NSABP FB-14). https://clinicaltrials.gov/study/NCT04024800?cond=NCT04024800&rank=1.

[328] National University Hospital, Singapore (2019). Nivolumab, Ipilimumab and OTSGC-A24 Therapeutic Peptide Vaccine in Gastric Cancer—A Combination Immunotherapy Phase Ib Study. (da VINci). https://clinicaltrials.gov/study/NCT03784040?cond=NCT03784040&rank=1.

[329] M.D. Anderson Cancer Center (2025). Personalized Peptide Vaccine in Treating Patients With Advanced Pancreatic Cancer or Colorectal Cancer. https://clinicaltrials.gov/study/NCT02600949?cond=NCT02600949&rank=1.

[330] Zhang Y., First Hospital of Shijiazhuang City (2020). Clinical Study of Neoantigen Vaccine Combined With Targeted Drugs in the Treatment of Non-small Cell Lung Cancer. https://clinicaltrials.gov/study/NCT04487093?cond=NCT04487093&rank=1.

[331] Mayo Clinic (2024). A Vaccine (PDS0101) Alone or in Combination With Pembrolizumab for the Treatment of Locally Advanced Human Papillomavirus-Associated Oropharynx Cancer. https://clinicaltrials.gov/study/NCT05232851?cond=NCT05232851&rank=1.

[332] Ann & Robert H Lurie Children’s Hospital of Chicago (2024). rHSC-DIPGVax Plus Checkpoint Blockade for the Treatment of Newly Diagnosed DIPG and DMG. https://clinicaltrials.gov/study/NCT04943848?cond=NCT04943848&rank=1.

[333] Roswell Park Cancer Institute (2024). Survivin Long Peptide Vaccine in Treating Patients With Metastatic Neuroendocrine Tumors. https://clinicaltrials.gov/study/NCT03879694?cond=NCT03879694&rank=1.

[334] Centre Hospitalier Universitaire de Besancon (2024). Combination of UCPVax Vaccine and Atezolizumab for the Treatment of Human Papillomavirus Positive Cancers (VolATIL) (VolATIL). https://clinicaltrials.gov/study/NCT03946358?cond=NCT03946358&rank=1.

[335] Fassi D.E., Herlev Hospital (2020). Peptide Vaccination in Combination With Azacitidine for Patients With MDS and AML (AZACTA). https://clinicaltrials.gov/study/NCT02750995?cond=NCT02750995&rank=1.

[336] University Hospital Tuebingen (2024). Personalized Multi-peptide Vaccination in Combination With the TLR1/2 Ligand XS15 in Cancer Patients (InHeVac01). https://clinicaltrials.gov/study/NCT05014607?cond=NCT05014607&rank=1.

[337] Kaumaya P.T.P., Indiana University (2025). HER-2 B Cell Peptide Vaccine. https://clinicaltrials.gov/study/NCT06414733?cond=NCT06414733&rank=1.

[338] Ott P., Dana-Farber Cancer Institute (2024). Neoantigen Vaccine Plus Locally Administered Ipilimumab and Systemic Nivolumab in Advanced Melanoma. https://clinicaltrials.gov/study/NCT03929029?cond=NCT03929029&rank=1.

[339] Raje N., Massachusetts General Hospital (2024). A Study of PVX-410, a Cancer Vaccine, and Citarinostat +/- Lenalidomide for Smoldering MM. https://clinicaltrials.gov/study/NCT02886065?cond=NCT02886065&rank=1.

[340] Sidney Kimmel Comprehensive Cancer Center at Johns Hopkins (2025). Pooled Mutant KRAS-Targeted Long Peptide Vaccine Combined With Nivolumab and Ipilimumab for Patients With Resected Mismatch Repair Protein (MMR-p) Colorectal and Pancreatic Cancer. https://clinicaltrials.gov/study/NCT04117087?cond=NCT04117087&rank=1.

[341] Sidney Kimmel Comprehensive Cancer Center at Johns Hopkins (2024). DNAJB1-PRKACA Fusion Kinase Peptide Vaccine Combined With Nivolumab and Ipilimumab for Patients With Fibrolamellar Hepatocellular Carcinoma. https://clinicaltrials.gov/study/NCT04248569?cond=NCT04248569&rank=1.

[342] Tewari A.K., Icahn School of Medicine at Mount Sinai (2024). The Safety and Tolerability of PGV001-based Personalized Multi-peptide Vaccines in the Adjuvant Setting. https://clinicaltrials.gov/study/NCT05010200?cond=NCT05010200&rank=1.

[343] Sidney Kimmel Comprehensive Cancer Center at Johns Hopkins (2025). KRAS-Targeted Vaccine With Nivolumab and Ipilimumab for Patients With NSCLC. https://clinicaltrials.gov/study/NCT05254184?cond=NCT05254184&rank=1.

[344] Diego E.J., University of Pittsburgh (2024). Prototype DAA/TAA Vaccine Targeting MUC1 for Immune Interception and Prevention in Ductal Carcinoma In Situ. https://clinicaltrials.gov/study/NCT06218303?cond=NCT06218303&rank=1.

[345] Sellas Life Sciences Group (2024). Galinpepimut-S in Combination With Pembrolizumab in Patients With Selected Advanced Cancers. https://clinicaltrials.gov/study/NCT03761914?cond=NCT03761914&rank=1.

[346] Mayo Clinic (2024). Personalized Neoantigen Peptide-Based Vaccine in Combination With Pembrolizumab for Treatment of Advanced Solid Tumors (PNeoVCA). https://clinicaltrials.gov/study/NCT05269381?cond=NCT05269381&rank=1.

[347] Lopes A., Vandermeulen G., Préat V. (2019). Cancer DNA vaccines: Current preclinical and clinical developments and future perspectives. J. Exp. Clin. Cancer Res..

[348] Strioga M.M., Darinskas A., Pasukoniene V., Mlynska A., Ostapenko V., Schijns V. (2014). Xenogeneic therapeutic cancer vaccines as breakers of immune tolerance for clinical application: To use or not to use?. Vaccine.

[349] Riccardo F., Bolli E., Macagno M., Arigoni M., Cavallo F., Quaglino E. (2017). Chimeric DNA Vaccines: An Effective Way to Overcome Immune Tolerance. Curr. Top. Microbiol. Immunol..

[350] Soong R.S., Trieu J., Lee S.Y., He L., Tsai Y.C., Wu T.C., Hung C.F. (2013). Xenogeneic human p53 DNA vaccination by electroporation breaks immune tolerance to control murine tumors expressing mouse p53. PLoS ONE.

[351] Sioud M., Sørensen D. (2003). Generation of an effective anti-tumor immunity after immunization with xenogeneic antigens. Eur. J. Immunol..

[352] Aurisicchio L., Roscilli G., Marra E., Luberto L., Mancini R., La Monica N., Ciliberto G. (2015). Superior Immunologic and Therapeutic Efficacy of a Xenogeneic Genetic Cancer Vaccine Targeting Carcinoembryonic Human Antigen. Hum. Gene Ther..

[353] Occhipinti S., Sponton L., Rolla S., Caorsi C., Novarino A., Donadio M., Bustreo S., Satolli M.A., Pecchioni C., Marchini C. (2014). Chimeric rat/human HER2 efficiently circumvents HER2 tolerance in cancer patients. Clin. Cancer Res..

[354] Quaglino E., Mastini C., Amici A., Marchini C., Iezzi M., Lanzardo S., De Giovanni C., Montani M., Lollini P.L., Masucci G. (2010). A better immune reaction to Erbb-2 tumors is elicited in mice by DNA vaccines encoding rat/human chimeric proteins. Cancer Res..

[355] Yuan J., Ku G.Y., Gallardo H.F., Orlandi F., Manukian G., Rasalan T.S., Xu Y., Li H., Vyas S., Mu Z. (2009). Safety and immunogenicity of a human and mouse gp100 DNA vaccine in a phase I trial of patients with melanoma. Cancer Immun..

[356] Yuan J., Ku G.Y., Adamow M., Mu Z., Tandon S., Hannaman D., Chapman P., Schwartz G., Carvajal R., Panageas K.S. (2013). Immunologic responses to xenogeneic tyrosinase DNA vaccine administered by electroporation in patients with malignant melanoma. J. Immunother. Cancer.

[357] Aurisicchio L., Pallocca M., Ciliberto G., Palombo F. (2018). The perfect personalized cancer therapy: Cancer vaccines against neoantigens. J. Exp. Clin. Cancer Res..

[358] Keskin D.B., Anandappa A.J., Sun J., Tirosh I., Mathewson N.D., Li S., Oliveira G., Giobbie-Hurder A., Felt K., Gjini E. (2019). Neoantigen vaccine generates intratumoral T cell responses in phase Ib glioblastoma trial. Nature.

[359] Li L., Zhang X., Wang X., Kim S.W., Herndon J.M., Becker-Hapak M.K., Carreno B.M., Myers N.B., Sturmoski M.A., McLellan M.D. (2021). Optimized polyepitope neoantigen DNA vaccines elicit neoantigen-specific immune responses in preclinical models and in clinical translation. Genome Med..

[360] Geneos Therapeutics (2024). GNOS-PV02 Personalized Neoantigen Vaccine, INO-9012 and Pembrolizumab in Subjects With Advanced HCC. https://clinicaltrials.gov/study/NCT04251117?cond=DNA%20Vaccine&term=Cancer%20Vaccine&start=2019-01-01_2024-12-31&aggFilters=status:com%20act%20not%20rec%20unk&page=3&rank=26.

[361] TuHURA Biosciences, Inc (2022). pDNA Intralesional Cancer Vaccine for Cutaneous Melanoma. https://clinicaltrials.gov/study/NCT03655756?cond=NCT03655756&rank=1.

[362] University of Washington (2024). STEMVAC in Patients With Early Stage Triple Negative Breast Cancer. https://clinicaltrials.gov/study/NCT05455658?cond=NCT05455658&rank=1.

[363] Bavarian Nordic (2025). TAEK-VAC-HerBy Vaccine for Brachyury and HER2 Expressing Cancer. https://clinicaltrials.gov/study/NCT04246671?cond=NCT04246671&rank=1.

[364] Disis M., National Cancer Institute (NCI) (2024). Vaccine Therapy in Preventing Cancer Recurrence in Patients With Non-Metastatic, Node Positive, HER2 Negative Breast Cancer That is in Remission (WOKVAC). https://clinicaltrials.gov/study/NCT02780401?cond=NCT02780401&rank=1.

[365] TuHURA Biosciences, Inc (2024). Immunotherapy With IFx-Hu2.0 Vaccine for Advanced Non-melanoma Skin Cancers. https://clinicaltrials.gov/study/NCT04160065?cond=cancer%20%20cell%20vaccine&start=2014-01-01_2024-12-31&term=Cancer%20Vaccine&intr=cancer%20cell&page=3&rank=28.

[366] Washington University School of Medicine (2025). Neoantigen-based Personalized DNA Vaccine in Patients With Newly Diagnosed, Unmethylated Glioblastoma. https://clinicaltrials.gov/study/NCT04015700?cond=NCT04015700&rank=1.

[367] Washington University School of Medicine (2024). Neoantigen-based Personalized DNA Vaccine With Retifanlimab PD-1 Blockade Therapy in Patients With Newly Diagnosed, Unmethylated Glioblastoma. https://clinicaltrials.gov/study/NCT05743595?cond=DNA%20Vaccine&term=Cancer%20Vaccine&start=2019-01-01_2024-12-31&aggFilters=status:com%20act%20not%20rec%20unk&page=2&rank=19.

[368] Washington University School of Medicine (2022). Neoantigen DNA Vaccine in Combination With Nivolumab/Ipilimumab and PROSTVAC in Metastatic Hormone-Sensitive Prostate Cancer. https://clinicaltrials.gov/study/NCT03532217?cond=NCT03532217&rank=1.

[369] University of Wisconsin, Madison (2024). pTVG-HP and Nivolumab in Patients With Non-Metastatic PSA-Recurrent Prostate Cancer. https://clinicaltrials.gov/study/NCT03600350?cond=NCT03600350&rank=1.

[370] University of Wisconsin, Madison (2025). pTVG-HP DNA Vaccine With or Without pTVG-AR DNA Vaccine and Pembrolizumab in Patients With Castration-Resistant, Metastatic Prostate Cancer. https://clinicaltrials.gov/study/NCT04090528?cond=NCT04090528&rank=1.

[371] Suschak J.J., Williams J.A., Schmaljohn C.S. (2017). Advancements in DNA vaccine vectors, non-mechanical delivery methods, and molecular adjuvants to increase immunogenicity. Hum. Vaccin. Immunother..

[372] Sidney Kimmel Comprehensive Cancer Center at Johns Hopkins (2025). HPV DNA Vaccine Via Electroporation for HPV16 Positive Cervical Neoplasia. https://clinicaltrials.gov/study/NCT04131413?cond=NCT04131413&rank=1.

[373] Merck Sharp & Dohme LLC (2024). Long-term Follow-up of Broad Spectrum Human Papillomavirus (HPV) Vaccine Study in Women (V503-021). https://clinicaltrials.gov/study/NCT02653118?cond=NCT02653118&rank=1.

[374] International Agency for Research on Cancer (2023). Impact of HPV Vaccination on Prevention of Cervical HPV Infection in Sikkim, India (HPV-Vac-S). https://clinicaltrials.gov/study/NCT04588402?cond=NCT04588402&rank=1.

[375] Washington University School of Medicine (2024). Neoepitope-based Personalized DNA Vaccine Approach in Pediatric Patients With Recurrent Brain Tumors. https://clinicaltrials.gov/study/NCT03988283?cond=NCT03988283&rank=1.

[376] Meleshko A., Belarusian Research Center for Pediatric Oncology, Hematology and Immunology (2022). DNA Vaccination Against Neuroblastoma. https://clinicaltrials.gov/study/NCT04049864?cond=NCT04049864&rank=1.

[377] Maeng H.M., National Cancer Institute (2024). Anti-PD-L1/TGF-beta Trap (M7824) Alone and in Combination With TriAd Vaccine and N-803 for Resectable Head and Neck Squamous Cell Carcinoma Not Associated With Human Papillomavirus Infection. https://clinicaltrials.gov/study/NCT04247282?cond=NCT04247282&rank=1.

[378] Yonsei University (2024). Trial of the Combination of GX-188E Vaccination, GX-I7 and Pembrolizumab in Patients With Advanced, Resectable HPV Type 16 and/or 18 Positive Head and Neck Cancer (GENUINE). https://clinicaltrials.gov/study/NCT05286060?cond=NCT05286060&rank=1.

[379] Lyerly H., Duke University (2025). Assessing the Immunogenicity of pING-hHER3FL. https://clinicaltrials.gov/study/NCT03832855?cond=NCT03832855&rank=1.

[380] Washington University School of Medicine (2025). Personalized Neoantigen Vaccine in Combination With Durvalumab (MEDI4736) in Extensive Stage Small Cell Lung Cancer. https://clinicaltrials.gov/study/NCT04397003?cond=NCT04397003&rank=1.

[381] University of Washington (2024). A Multiple Antigen Vaccine (STEMVAC) for the Treatment of Patients With Stage IV Non-Small Cell Lung Cancer. https://clinicaltrials.gov/study/NCT05242965?cond=NCT05242965&rank=1.

[382] Newish Technology (Beijing) Co., Ltd. (2023). NWRD06 DNA Plasmid for HCC After Radical Resection. https://clinicaltrials.gov/study/NCT06088459?cond=NCT06088459&rank=1.

[383] Liu C., Shi Q., Huang X., Koo S., Kong N., Tao W. (2023). mRNA-based cancer therapeutics. Nat. Rev. Cancer.

[384] Pardi N., Hogan M.J., Porter F.W., Weissman D. (2018). mRNA vaccines—A new era in vaccinology. Nat. Rev. Drug Discov..

[385] He Q., Gao H., Tan D., Zhang H., Wang J.Z. (2022). mRNA cancer vaccines: Advances, trends and challenges. Acta Pharm. Sin. B.

[386] Mai Y., Guo J., Zhao Y., Ma S., Hou Y., Yang J. (2020). Intranasal delivery of cationic liposome-protamine complex mRNA vaccine elicits effective anti-tumor immunity. Cell Immunol..

[387] Weide B., Pascolo S., Scheel B., Derhovanessian E., Pflugfelder A., Eigentler T.K., Pawelec G., Hoerr I., Rammensee H.G., Garbe C. (2009). Direct injection of protamine-protected mRNA: Results of a phase 1/2 vaccination trial in metastatic melanoma patients. J. Immunother..

[388] Sahin U., Oehm P., Derhovanessian E., Jabulowsky R.A., Vormehr M., Gold M., Maurus D., Schwarck-Kokarakis D., Kuhn A.N., Omokoko T. (2020). An RNA vaccine drives immunity in checkpoint-inhibitor-treated melanoma. Nature.

[389] Estapé Senti M., García Del Valle L., Schiffelers R.M. (2024). mRNA delivery systems for cancer immunotherapy: Lipid nanoparticles and beyond. Adv. Drug Deliv. Rev..

[390] Tenchov R., Bird R., Curtze A.E., Zhou Q. (2021). Lipid Nanoparticles─From Liposomes to mRNA Vaccine Delivery, a Landscape of Research Diversity and Advancement. ACS Nano.

[391] Merck Sharp & Dohme LLC (2025). A Study of V940 Plus Pembrolizumab (MK-3475) Versus Placebo Plus Pembrolizumab in Participants With Non-small Cell Lung Cancer (V940-002) (INTerpath-002). https://clinicaltrials.gov/study/NCT06077760?cond=mRNA-4157&rank=5.

[392] Merck Sharp & Dohme LLC (2024). A Clinical Study of V940 Plus Pembrolizumab in People With High-Risk Melanoma (V940-001). https://clinicaltrials.gov/study/NCT05933577?cond=mRNA-4157&rank=3.

[393] Weber J.S., Carlino M.S., Khattak A., Meniawy T., Ansstas G., Taylor M.H., Kim K.B., McKean M., Long G.V., Sullivan R.J. (2024). Individualised neoantigen therapy mRNA-4157 (V940) plus pembrolizumab versus pembrolizumab monotherapy in resected melanoma (KEYNOTE-942): A randomised, phase 2b study. Lancet.

[394] Gainor J.F., Patel M.R., Weber J.S., Gutierrez M., Bauman J.E., Clarke J.M., Julian R., Scott A.J., Geiger J.L., Kirtane K. (2024). T-cell Responses to Individualized Neoantigen Therapy mRNA-4157 (V940) Alone or in Combination with Pembrolizumab in the Phase 1 KEYNOTE-603 Study. Cancer Discov..

[395] BioNTech SE (2025). Trial With BNT111 and Cemiplimab in Combination or as Single Agents in Patients With Anti-PD-1-refractory/Relapsed, Unresectable Stage III or IV Melanoma. https://clinicaltrials.gov/study/NCT04526899?cond=NCT04526899&rank=1.

[396] ModernaTX, Inc. (2024). An Efficacy Study of Adjuvant Treatment With the Personalized Cancer Vaccine mRNA-4157 and Pembrolizumab in Participants With High-Risk Melanoma (KEYNOTE-942). https://clinicaltrials.gov/study/NCT03897881?cond=NCT03897881&rank=1.

[397] Peking Union Medical College Hospital (2023). A Clinical Study of mRNA Vaccine (ABOR2014/IPM511) in Patients With Advanced Hepatocellular Carcinoma. https://clinicaltrials.gov/study/NCT05981066?cond=mRNA%20vaccine&lastUpdPost=2019-01-01_&aggFilters=status:act%20com%20not%20rec%20unk%20sus%20wit%20enr&page=12&rank=116.

[398] Peng X., West China Hospital (2023). Application of mRNA Immunotherapy Technology in Hepatitis B Virus-related Refractory Hepatocellular Carcinoma. https://clinicaltrials.gov/study/NCT05738447?cond=mRNA%20vaccine&lastUpdPost=2019-01-01_&aggFilters=status:act%20com%20not%20rec%20unk%20sus%20wit%20enr&page=4&rank=40.

[399] Shanghai Zhongshan Hospital (2023). Clinical Study of mRNA Vaccine in Patients With Liver Cancer After Operation. https://clinicaltrials.gov/study/NCT05761717?cond=mRNA%20vaccine&lastUpdPost=2019-01-01_&aggFilters=status:act%20com%20not%20rec%20unk%20sus%20wit%20enr&page=6&rank=59.

[400] Ludwig Institute for Cancer Research (2022). Phase 1/2 Study of Combination Immunotherapy and Messenger Ribonucleic Acid (mRNA) Vaccine in Subjects With NSCLC. https://clinicaltrials.gov/study/NCT03164772?cond=mRNA%20vaccine&lastUpdPost=2019-01-01_&aggFilters=status:act%20com%20not%20rec%20unk%20sus%20wit%20enr&page=6&rank=51.

[401] Guangdong Provincial People’s Hospital (2024). MRNA Neoantigen Vaccine in Non-Small Cell Lung Cancer. https://clinicaltrials.gov/study/NCT06735508?cond=mRNA%20vaccine&lastUpdPost=2019-01-01_&aggFilters=status:act%20com%20not%20rec%20unk%20sus%20wit%20enr&page=26&rank=252.

[402] Wang S., Jinling Hospital, China (2024). Study of Neoantigen mRNA Vaccines in Patients With Resectable Pancreatic Cancer. https://clinicaltrials.gov/study/NCT06326736?cond=mRNA%20vaccine&lastUpdPost=2019-01-01_&aggFilters=status:act%20com%20not%20rec%20unk%20sus%20wit%20enr&page=2&rank=16.

[403] Ruijin Hospital (2024). Study of KRAS Neoantigen mRNA Vaccine (ABO2102) in Patients With KRAS -Mutated Advanced Pancreatic Cancer. https://clinicaltrials.gov/study/NCT06577532?cond=mRNA%20vaccine&lastUpdPost=2019-01-01_&aggFilters=status:act%20com%20not%20rec%20unk%20sus%20wit%20enr&page=10&rank=93.

[404] Ruijin Hospital (2024). Clinical Study of XP-004 Personalized mRNA Tumor Vaccine Combined With PD-1 Inhibitor for Postoperative Adjuvant Therapy for Pancreatic Cancer in Patients With Advanced Solid Tumors. https://clinicaltrials.gov/study/NCT06496373?cond=mRNA%20vaccine&lastUpdPost=2019-01-01_&aggFilters=status:act%20com%20not%20rec%20unk%20sus%20wit%20enr&page=15&rank=143.

[405] Yu X., Fudan University (2023). Study of Personalized Tumour Vaccines and a PD-L1 Blocker in Patients With Surgically Resected Pancreatic Adenocarcino. https://clinicaltrials.gov/study/NCT06156267?cond=mRNA%20vaccine&lastUpdPost=2019-01-01_&aggFilters=status:act%20com%20not%20rec%20unk%20sus%20wit%20enr&page=14&rank=139.

[406] Wu W., Peking Union Medical College Hospital (2024). XH001 Combination With Ipilimumab and Chemotherapy for Patients With Resected Pancreatic Cancer. https://clinicaltrials.gov/study/NCT06353646?cond=mRNA%20vaccine&lastUpdPost=2019-01-01_&aggFilters=status:act%20com%20not%20rec%20unk%20sus%20wit%20enr&page=21&rank=210.

[407] Memorial Sloan Kettering Cancer Center (2025). Study of Personalized Tumor Vaccines (PCVs) and a PD-L1 Blocker in Patients With Pancreatic Cancer That Can be Treated With Surgery. https://clinicaltrials.gov/study/NCT04161755?cond=NCT04161755&rank=1.

[408] Merck Sharp & Dohme LLC (2025). A Study of (Neo)Adjuvant V940 and Pembrolizumab in Cutaneous Squamous Cell Carcinoma (V940-007). https://clinicaltrials.gov/study/NCT06295809?cond=mRNA-4157&rank=4.

[409] Merck Sharp & Dohme LLC (2025). A Study of Adjuvant V940 and Pembrolizumab in Renal Cell Carcinoma (V940-004). (INTerpath-004). https://clinicaltrials.gov/study/NCT06307431?cond=mRNA-4157&rank=6.

[410] BioNTech SE (2025). A Clinical Trial Investigating the Safety, Tolerability, and Therapeutic Effects of BNT113 in Combination With Pembrolizumab Versus Pembrolizumab Alone for Patients With a Form of Head and Neck Cancer Positive for Human Papilloma Virus 16 and Expressing the Protein PD-L1 (AHEAD-MERIT). https://clinicaltrials.gov/study/NCT04534205?cond=mRNA%20vaccine&lastUpdPost=2019-01-01_&aggFilters=status:act%20com%20not%20rec%20unk%20sus%20wit%20enr&page=26&rank=255.

[411] Peng X., West China Hospital (2023). Application of mRNA Immunotherapy Technology in Epstein-Barr Virus-related Refractory Malignant Tumors. https://clinicaltrials.gov/study/NCT05714748?cond=mRNA%20vaccine&lastUpdPost=2019-01-01_&aggFilters=status:act%20com%20not%20rec%20unk%20sus%20wit%20enr&page=3&rank=23.

[412] Cai X., Sir Run Run Shaw Hospital (2023). Clinical Study of Personalized mRNA Vaccine Encoding Neoantigen Alone in Subjects With Advanced Digestive System Neoplasms. https://clinicaltrials.gov/study/NCT06019702?cond=mRNA%20vaccine&lastUpdPost=2019-01-01_&aggFilters=status:act%20com%20not%20rec%20unk%20sus%20wit%20enr&page=6&rank=52.

[413] Cai X., Sir Run Run Shaw Hospital (2023). Clinical Study of Personalized mRNA Vaccine Encoding Neoantigen in Subjects With Resected Digestive System Neoplasms. https://clinicaltrials.gov/study/NCT06026774?cond=mRNA%20vaccine&lastUpdPost=2019-01-01_&aggFilters=status:act%20com%20not%20rec%20unk%20sus%20wit%20enr&page=9&rank=89.

[414] Wang B., Changhai Hospital (2019). Clinical Study of Personalized mRNA Vaccine Encoding Neoantigen in Patients With Advanced Digestive System Neoplasms. https://clinicaltrials.gov/study/NCT03468244?cond=mRNA%20vaccine&lastUpdPost=2019-01-01_&aggFilters=status:act%20com%20not%20rec%20unk%20sus%20wit%20enr&page=11&rank=106.

[415] Cai X., Sir Run Run Shaw Hospital (2023). Clinical Study of Personalized mRNA Vaccine Encoding Neoantigen in Combination With Standard First-line Treatment in Subjects With Advanced Digestive System Neoplasms. https://clinicaltrials.gov/study/NCT06026800?cond=mRNA%20vaccine&lastUpdPost=2019-01-01_&aggFilters=status:act%20com%20not%20rec%20unk%20sus%20wit%20enr&page=11&rank=102.

[416] Stemirna Therapeutics (2023). Clinical Study of Personalized mRNA Vaccine Encoding Neoantigen in Patients With Advanced Esophageal Cancer and Non-small Cell Lung Cancer. https://clinicaltrials.gov/study/NCT03908671?cond=mRNA%20vaccine&lastUpdPost=2019-01-01_&aggFilters=status:act%20com%20not%20rec%20unk%20sus%20wit%20enr&page=13&rank=124.

[417] Ning G., Shanghai Jiao Tong University School of Medicine (2024). Treatment of Advanced Endocrine Tumor With Iindividualized mRNA Neoantigen Vaccine (mRNA-0523-L001). https://clinicaltrials.gov/study/NCT06141369?cond=mRNA%20vaccine&lastUpdPost=2019-01-01_&aggFilters=status:act%20com%20not%20rec%20unk%20sus%20wit%20enr&page=9&rank=87.

[418] Esserman L., University of California, San Francisco (2025). Immunotherapy in High-risk Ductal Carcinoma in Situ (DCIS). https://clinicaltrials.gov/study/NCT02872025?cond=NCT02872025&rank=1.

[419] ModernaTX, Inc. (2025). Safety, Tolerability, and Immunogenicity of mRNA-4157 Alone and in Combination in Participants With Solid Tumors (KEYNOTE-603). https://clinicaltrials.gov/study/NCT03313778?cond=mRNA-4157&rank=1.

[420] Second Affiliated Hospital of Guangzhou Medical University (2024). Anti-cancer Neoantigen mRNA Vaccine to Treat Solid Tumors. https://clinicaltrials.gov/study/NCT06195384?cond=mRNA%20vaccine&lastUpdPost=2019-01-01_&aggFilters=status:act%20com%20not%20rec%20unk%20sus%20wit%20enr&page=2&rank=11.

[421] Stemirna Therapeutics (2022). A Study of Neoantigen mRNA Personalised Cancer in Patients With Advanced Solid Tumors. https://clinicaltrials.gov/study/NCT05198752?cond=mRNA%20vaccine&lastUpdPost=2019-01-01_&aggFilters=status:act%20com%20not%20rec%20unk%20sus%20wit%20enr&page=8&rank=76.

[422] Stemirna Therapeutics (2023). Clinical Study of mRNA Vaccine in Patients With Advanced Malignant Solid Tumors. https://clinicaltrials.gov/study/NCT05949775?cond=mRNA%20vaccine&lastUpdPost=2019-01-01_&aggFilters=status:act%20com%20not%20rec%20unk%20sus%20wit%20enr&page=4&rank=38.

[423] Su S., The Affiliated Hospital Of Guizhou Medical University (2024). An Exploratory Study of Individualized Neo-antigen MRNA Cancer Vaccine InnoPCV in Advanced Solid Tumor Treatment. https://clinicaltrials.gov/study/NCT06497010?cond=mRNA%20vaccine&lastUpdPost=2019-01-01_&aggFilters=status:act%20com%20not%20rec%20unk%20sus%20wit%20enr&page=26&rank=258.

[424] Xu J., The Affiliated Hospital of the Chinese Academy of Military Medical Sciences (2023). A Safety and Efficacy Study of XH001 Combined With Sintilimab Injection in Advanced Solid Tumors. https://clinicaltrials.gov/study/NCT05940181?cond=mRNA%20vaccine&lastUpdPost=2019-01-01_&aggFilters=status:act%20com%20not%20rec%20unk%20sus%20wit%20enr&page=18&rank=171.

[425] BioNTech SE (2024). Dose Escalation Trial of BNT152+153 in Patients With Cancer. https://clinicaltrials.gov/study/NCT04710043?cond=NCT04710043&rank=1.

[426] ModernaTX, Inc. (2024). Dose Escalation Study of mRNA-2752 for Intratumoral Injection to Participants in Advanced Malignancies. https://clinicaltrials.gov/study/NCT03739931?cond=NCT03739931&rank=1.

[427] MedImmune LLC (2024). A Study of MEDI1191 in Sequential and Concurrent Combination With Durvalumab in Subjects With Advanced Solid Tumors. https://clinicaltrials.gov/study/NCT03946800?cond=NCT03946800&rank=1.

[428] Rosenberg S., National Cancer Institute (NCI) (Responsible Party) (2020). Messenger RNA (mRNA)-Based, Personalized Cancer Vaccine Against Neoantigens Expressed by the Autologous Cancer. https://clinicaltrials.gov/study/NCT03480152?cond=NCT03480152&rank=1.

[429] Merck (2022). Moderna and Merck Announce mRNA-4157/V940, an Investigational Personalized mRNA Cancer Vaccine, in Combination With KEYTRUDA® (pembrolizumab), Met Primary Efficacy Endpoint in Phase 2b KEYNOTE-942 Trial. https://www.merck.com/news/moderna-and-merck-announce-mrna-4157-v940-an-investigational-personalized-mrna-cancer-vaccine-in-combination-with-keytruda-pembrolizumab-met-primary-efficacy-endpoint-in-phase-2b-keynote-94/.

